# MycoNews 2020: President’s message, news, reports, awards, personalia, book news, and correspondence

**DOI:** 10.1186/s43008-020-00049-5

**Published:** 2020-12-31

**Authors:** David L. Hawksworth

**Affiliations:** 1 Comparative Plant and Fungal Biology, Royal Botanic Gardens, Kew, Surrey TW9 3DS UK; 2 Department of Life Sciences, The Natural History Museum, Cromwell Road, London, SW7 5BD UK; 3 Jilin Agricultural University, Changchun, 130118 Jilin Province China

**Keywords:** Book reviews, COVID-19, Funga, Fungi, International mycological congress, Meeting reports, Obituaries, Birthday tributes

## Abstract

This second annual edition of *MycoNews* starts with a message from IMA President Wieland Meyer regarding the steps being taken to legally incorporate the Association in Switzerland. News is provided on progress in the arrangements for IMC12 (Amsterdam 2022), release of the *State of World’s Plants and Fungi* report, mycology under the Coronavirus pandemic, and two new biodiversity initiatives in The Netherlands. Reports are presented from the: 1st International Symposium on Tropical African Mycology (Benin 2019); Recent Advances in the Biodiversity, Biology, and Biotechnology of Fungi (Pondicherry 2019); the 4th International *Malassezia* Workshop (virtual 2020); Dutch Design Week (2020), and UK Fungus Day (2020). An honour awarded to Francis Martin is recorded. Birthday greetings from IMA go to John Walker, José Dianese, Richard Harris, Tomasz Majewski, David Malloch, and John Sheard. Tributes are paid to the passing of John Peberdy, Anthony Trinci, and Balamuri Vittal. This contribution continues with news of 10 new mycological books published in 2019 or 2020 and concludes with a letter on the appropriateness of adopting the term ‘Funga’.

STOP PRESS! Just the day after the copy for *MycoNews 2020* went to Press, this long-awaited monograph of *Orbiliomycetes* landed on my desk; two A4 size volumes together weighing 6.9 kg. This is such an important world monograph and stunning book that it merited being noted right away. An amazing 470 species are accepted, of which 331 are newly described; a further 90 are introduced, but without valid names. The class has but a single order and family, but includes seven genera, with *Orbilia* by far the largest with around 415 species. The generic concept for *Orbilia* is especially broad, polyphyletic, and embraces several genera with types that are asexual morphs as synonyms. The taxonomic concepts and methods of examination are described in detail, as is each species, and with superb colour photomicrographs and line drawings. All who struggle to identify these fascinating little discomycetes will want a copy, which is modestly priced as a hard copy for order from the museum in Luxembourg (http://www.mnhn.lu/ferrantia), but pleasingly it is also available for download free of charge in PDF format (https://www.mnhn.lu/science/monograph-of-orbiliomycetes/?lang=en)! It is something all major mycological collections will want to secure for their shelves while stocks of hard copies are available.

**Baral H-O, Weber E, Marson G (2020) Monograph of** ***Orbiliomycetes*** **(*Ascomycota*) based on vital taxonomy. 2 vols. 1752 pp., 1028 figs (many col.). Luxembourg: National Museum of Natural History. ISBN 978-2-91877-24-9. Price: 150 € (hbk) or free (PDF).**

## PRESIDENT’S MESSAGE 2020

(Figs. 1 and 2)

**Fig. 1:**
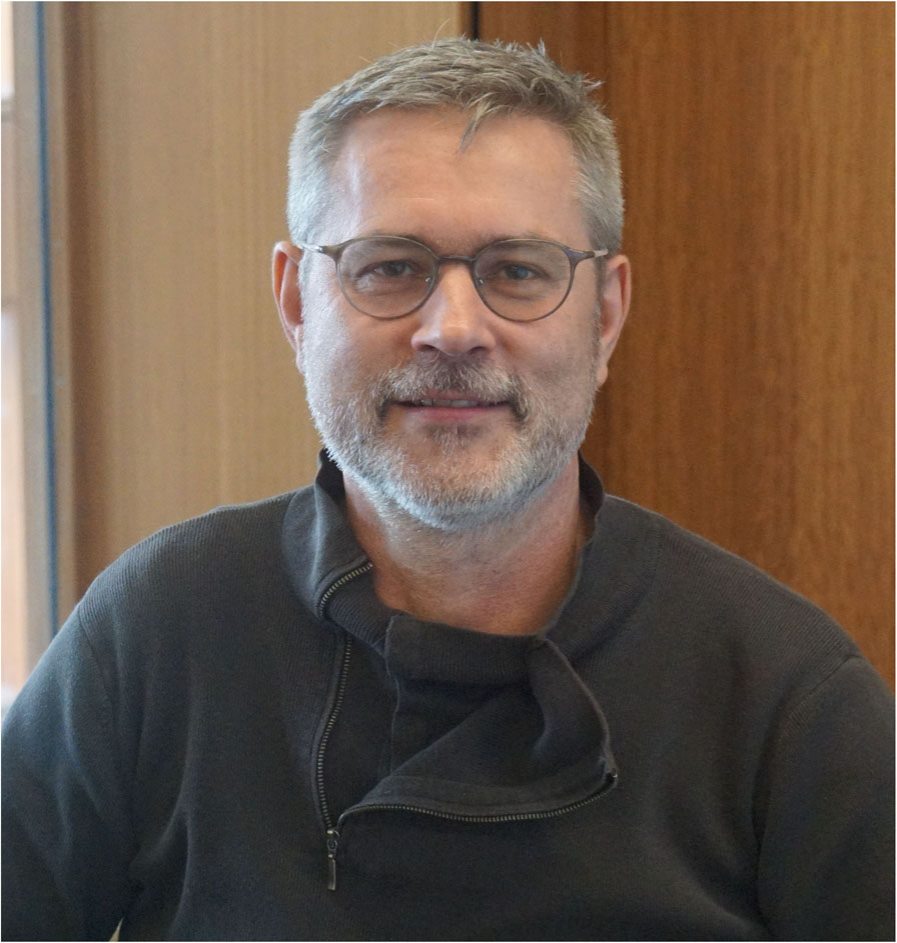
Wieland Meyer

**Fig. 2:**
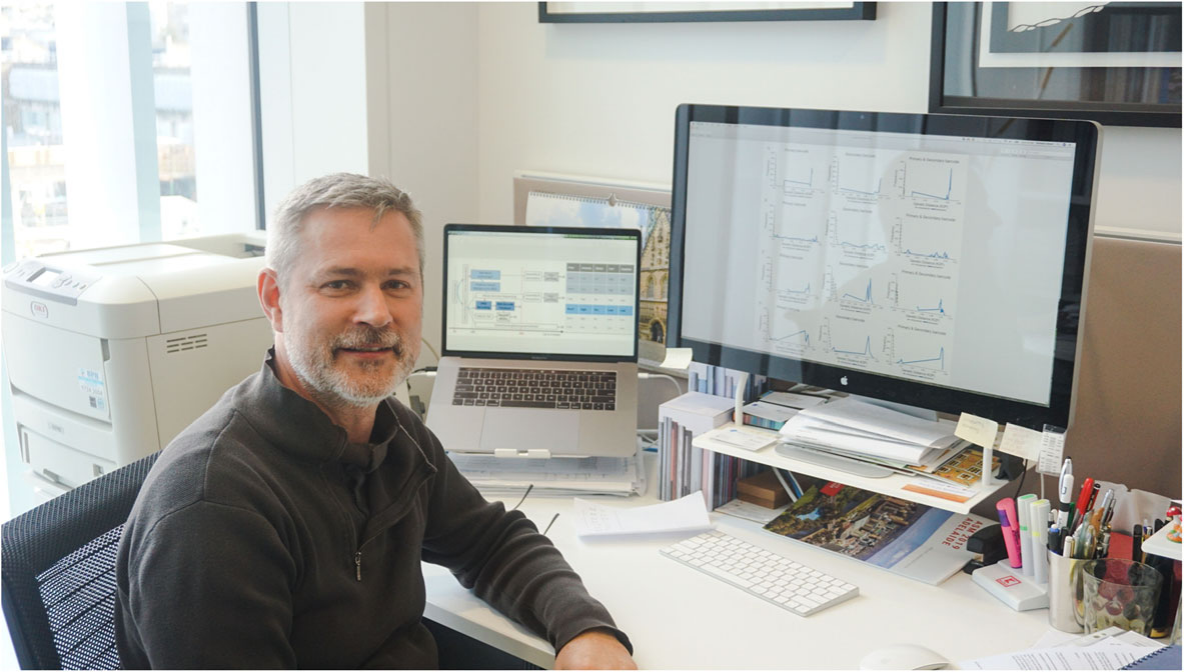
Wieland Meyer in his office

Fellow Mycologists, a challenging year is coming to a close, in which we all had to adjust to new ways of life in the shadow of COVID-19. We learned to work increasingly online and got used to alternative ways the scientific community is exchanging their research results, discusses new findings and set up new collaborations *via* virtual conferences. The use of online meeting platforms, although challenging, opened up new opportunities to communicate, which we, as the IMA Executive Committee, have actively used to progress major issues on the IMA agenda.

When the new IMA officers took over the business of IMA at the 11th International Mycological Congress (IMC11) in Puerto Rico in 2018, there were two major projects ahead of us: (1) to finalize the process of finding a professional publisher for our flagship journal *IMA Fungus*; and (2) to officially incorporate the IMA. Since then we made great progress by appointing BMC/Springer as the professional publisher for *IMA Fungus*, which has led to a significant saving, as the publication is now realized without additional costs to the IMA, and this year for the first time has started to generate a moderate income! *IMA Fungus,* under the leadership of David Hawksworth as Editor-in-Chief, had an Impact Factor of 3.636 for 2019. We then took on the challenge to incorporate IMA.

***Why does IMA need to be incorporated?*** The IMA was started on an initiative of the British Mycological Society, in collaboration with many global mycologists and societies, in order to establish an independent International Mycological Congress (IMC). As a result, the first IMC was held in Exeter in 1971 and the IMA was established as an international society fostering all aspects of mycology. Thanks in 1971 and the IMA was established as an international society fostering all aspects of mycology. Since 1971 it has become the leading platform to represent mycology globally, now organizing very successful IMCs every 4 years, tackling taxonomic and nomenclature issues of fungi, and supporting MycoBank as one of the centralized fungal name repositories. With the founders and the subsequent leaderships being focused on the scientific side of mycology, they and their member organizations never thought the legal incorporation of IMA as a non-for-profit organization was a necessity. However, over time national and international laws have changed, especially in the financial context, and in order to enable the holding and transfer of funds to further scientific research and raise public and political awareness about all mycological matters, incorporation became essential and increasingly urgent.

***What is the approach for the incorporation?*** After exploring a number of options, a process started under the presidency of Keith Seifert, the new IMA Executive Committee then investigated a number of options, including the possibility of seeking incorporation in Australia, China, or Germany – but without success. Based on my experience from the International Society for Human and Animal Mycology (ISHAM), which had already undergone a similar process, the IMA Executive Committee decided to seek incorporation in Switzerland. For this process, it was decided to work with the same Swiss law firm which had been used by ISHAM, Ludwig & Partners in Basel, being aware that this comes at a cost for the IMA.

***Updating the IMA statutes.*** As part of the incorporation process in Switzerland it was necessary to bring the IMA Statutes into line with Swiss law and to officially found IMA with a base in Switzerland. A small working group, consisting of Jennifer Luangsa-ard (General Secretary), Keith Seifert (Past-President), Orlando Petrini (Executive Committee member and contact for the Swiss lawyers) and myself (President), has worked on preparing the required new IMA Statutes, maintaining the principles of the IMA and combining them with the requirements of Swiss law for non-for-profit organizations incorporated in Switzerland. The resulting draft was then circulated to all IMA Executive Committee members, including representatives of all member mycological organizations (MMOs, SMMOs, and RMMOs). Changes and corrections received by 26 August 2020 are being incorporated, and the revised Statutes will be published in *IMA Fungus* and also will be made available on the IMA webpage as soon as they have been verified by the Swiss layers.

***What are the major changes in the new IMA statutes?*** **(A)** The IMA will have a general membership based beyond the attendees of an IMC, something hardly done in its history. The following member categories will now exist: (1) Individual Members, (2) Student Members, (3) Regional Member Mycological Organizations (RMMOs), (3) Sustaining Member Mycological Organizations (SMMOs), (4) Member Mycological Organizations (MMOs) and (5) Honorary Members. **(B)** The official organizational bodies of the IMA will be: (1) the General Assembly; (2) the IMA Council (the Managing Body of IMA), consisting of the President, President-elect, General Secretary, Treasurer, a Vice-president (Chair of the past IMC), a Vice-president (Chair of the next IMC), the Past-President (as an ex-officio member), and Editor(s)-in-Chief of the association’s journal(s) (as ex-officio member(s); (3) the IMA Executive Committee (the Strategic Body of IMA), consisting of 12 elected members nominated by the IMA members, a representative of each of the RMMOs and SMMOs, and Honorary Presidents (ex-officio members); and (4) the Auditors. **(C)** There will be a four-year individual membership fee, and annual fees for the SMMOs and MMOs, to be set by the IMA Council in correspondence with the IMA Executive Committee, to be ratified by the next IMA General Assembly.

***What are the next steps?*** Following the verification of the new statutes by the Swiss law firm, we will then call for an online foundation meeting, comprising of all current IMA Officers, Executive Committee members, and a representative of each MMO, SMMO and RMMO, to set up the IMA as a legal entity in Switzerland and to bring it in line with the legal requirements according to Swiss law.

We are aiming to complete the official incorporation of IMA in the next few months, well in advance of the IMC12 in Amsterdam in 2022, enabling the then incoming IMA Council and Executive Committee to fully focus on the scientific, political, and public aspects of mycology.

I thank all of you, and especially the IMA Officers, who have actively contributed to the incorporation process and the restructuring of the new Statutes, and all of you, who have confirmed their individual mycological societies’ support of the process. The IMA Executive Committee is looking forward to your continuing support of IMA, and over the next months we will continue to focus on the successful organisation of IMC12.

As President of the IMA, I wish you all a safe upcoming festive season and a good start in the new year.

**Wieland Meyer**

*President, IMA*

(wieland.meyer@sydney.edu.au)

## NEWS

### IMC12–2022: a life-changing experience

#### (www.imc12.org)

(Fig. 3)

**Fig. 3:**
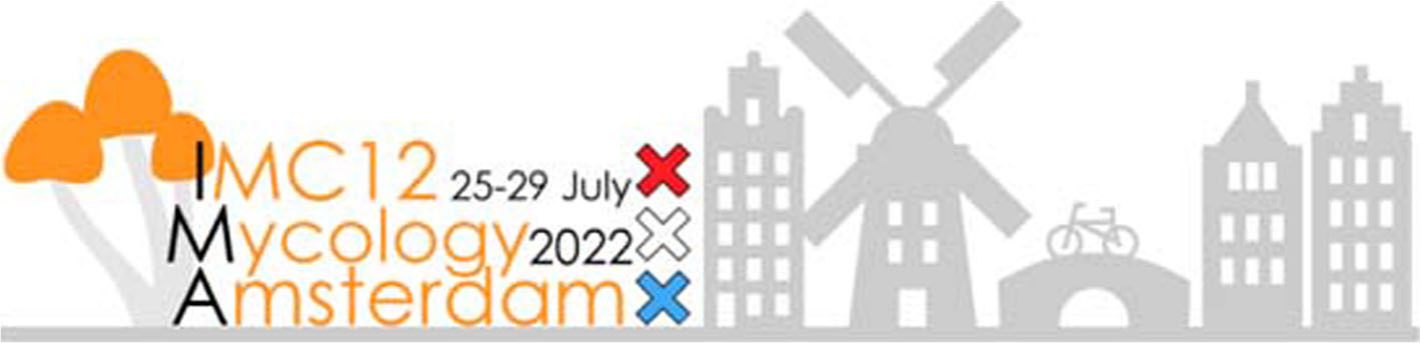
IMC12 2020 Banner

The Dutch Mycological Society, in collaboration with the Westerdijk Fungal Biodiversity Institute, is pleased to invite you to attend the 12th International Mycological Congress (IMC12) in Amsterdam on 25–29 July 2022. We are excited for the return of this incredible mycological experience to Europe. For the first time, an IMC will be hosted in The Netherlands, home to famous mycologists like Antonie van Leeuwenhoek, Hendrik Persoon, Johanna Westerdijk, J. A.(‘Dolf’) von Arx, Cornelius Bas, Joop van Brummelen, Walter Gams, Jos Wessels, Kreger-van Rij, and many, many more. In January 2021, there will be a call for **Symposia**, and **Workshops**, and we will keep you posted!

The **Scientific Themes** have now been established, and the Chairs appointed. Each theme also has its own scientific committee.

*General chair*: Pedro Crous

*Scientific Chair*: Teun Boekhout

**Theme1: Cell biology, biochemistry, and physiology**

- Chair 1, Ida van der Klei, NL
- Chair 2, Gregory Jedd, Singapore
- Chair 3, Marcio Rodriques, Brazil

**Theme 2: Environment, ecology and interactions**

- Chair 1, Lynne Boddy, UK
- Chair 2, Duur Aanen, NL
- Chair 3, Yu Fukasawa, Japan

**Theme 3: Evolution, biodiversity, and systematics**

- Chair 1, Jos Houbraken, NL
- Chair 2, Ester Gaya, UK
- Chair 3, Lei Cai, China

**Theme 4: Fungal pathogenesis and disease control**

- Chair 1, Dee Carter, Australia
- Chair 2, Martijn Rep, NL
- Chair 3, Juan McEwen, Colombia

**Theme 5: Genomics, genetics, and molecular biology**

- Chair 1, Miia Mäkelä, Finland
- Chair 2, Ronald de Vries, NL
- Chair 3: Brenda Wingfield, South Africa

**Theme 6: Nomenclature**

- Chair 1, Tom May, Australia
- Chair 2, Konstanze Bensch, Germany
- Chair 3, Bevan Weir, New Zealand

**Theme 7: Applied mycology**

- Chair 1, Lene Jesperson, Denmark
- Chair 2, Richard Bélanger, Canada
- Chair 3, Nancy Keller, USA

The first **Keynote Speakers** can now be announced. Each keynote session will have two speakers, and starting in 2021 new keynotes will be announced in IMC12 newsletters as they are confirmed.

### Nomenclature

#### M. Catherine Aime, USA 

(Fig. 4)

Cathie earned her doctorate in Biology from Virginia Polytechnic Institute and State University in 2001 under the tutelage of Orson K. Miller and completed her postdoctoral research at the University of Oxford in the United Kingdom in the lab of Lorna Casselton. She worked for 4 years as a research molecular biologist with the USDA-ARS, Systematic Botany and Mycology Lab in Beltsville, MD, and for 4 years as an Assistant and Associate Professor at Louisiana State University. Currently she is Professor of Mycology in the Department of Botany and Plant Pathology and Director of the Arthur and Kriebel Herbaria at Purdue University in West Lafayette, IN. The Aime Lab researches the systematics and evolution of early diverging basidiomycetes and of tropical fungi. Of particular interest are rust fungi and fungal diseases of tropical tree crops such as coffee and cacao and their socio-economic impacts on rural farmers in developing economies. Cathie is a Fellow of the Mycological Society of America (MSA), the Explorer’s Club, and the Linnean Society of London, a former officer of the MSA, past Managing Editor of *Mycologia*, and a Purdue University Faculty Scholar.

**Fig. 4:**
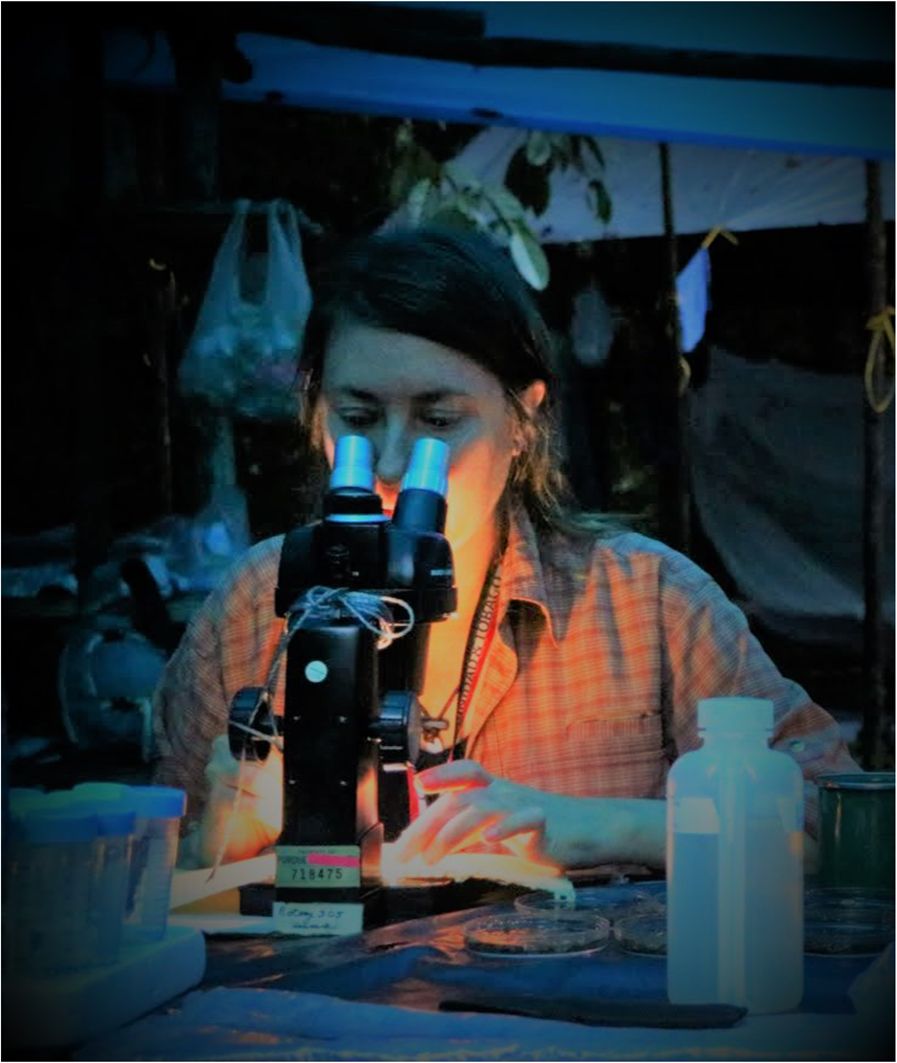
Cathie Aime

#### Robert Lücking, Germany 

(Fig. 5)

Robert obtained his PhD in Natural Sciences from the University of Ulm (Germany) in 1994. After a visiting professorship at the Federal University of Pernambuco, Recife (Brazil) in 1998 and a postdoc to obtain his habilitation in Botany at the University of Bayreuth (Germany) in 2001, he became Collections Manager & Adjunct Curator (Lichens & Fungi) at the Field Museum, Chicago, where he worked until 2015. Since 2015, Robert has been Curator (Lichens, Fungi, Bryophytes) at the Botanic Garden and Botanical Museum of the Freie Universität Berlin, Germany. Robert’s research focuses on tropical lichens (taxonomy, systematics, evolution, ecology, biogeography, uses) and more recently on fungal evolution, systematics, and nomenclature.

**Fig. 5:**
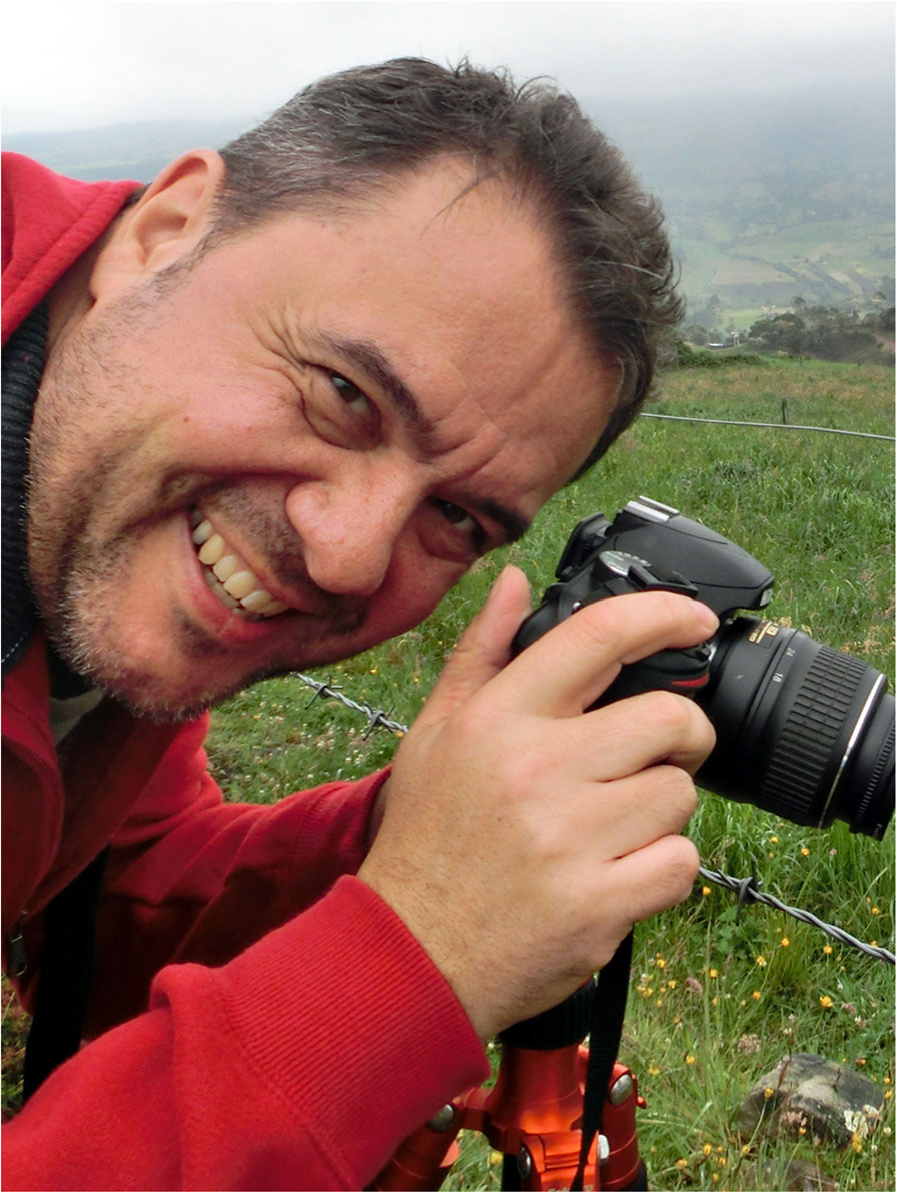
Robert Lücking

### Evolution, biodiversity and systematics

#### Toni Gabaldón, Spain

(Fig. 6)

Toni is a research professor, and group leader at the Institute for Research in Biomedicine (IRB) and the Barcelona Supercomputing Centre (BSC), and associate professor at the UPF. He leads the comparative genomics groups. His main research interests are in the fields of genomics and evolution with significant contributions to the understanding of how genes, pathways, organisms, and communities evolve and function.

**Fig. 6:**
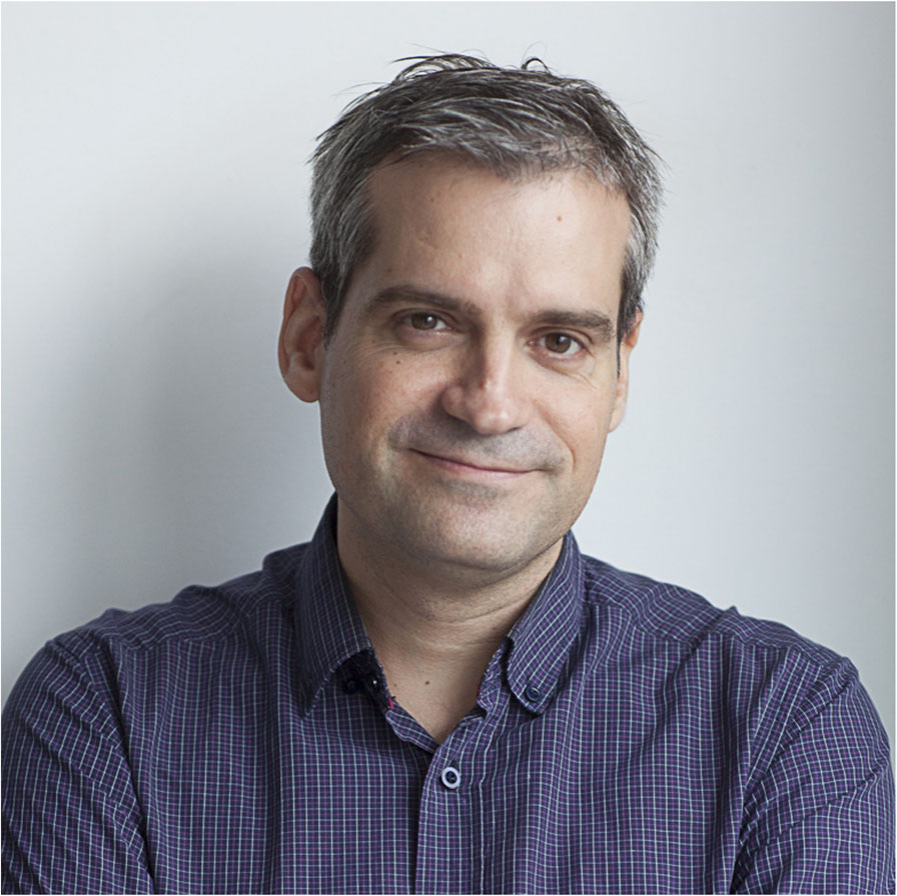
Toni Gabaldón

#### Jolanta Maria Miadłikowska, USA

(Fig. 7)

Jolanta a senior tesearcher in Duke University’s Department of Biology, Durham, NC. She received her PhD in Biology from the University of Gdansk in Poland in 1999 and completed 5 years of postdoctoral research at Duke. She is a systematist interested in biodiversity, taxonomy, phylogenetics, and evolution of lichenized *Ascomycota*, with a special emphasis on cyanolichens from the genus *Peltigera* and their cyanobacterial photobiont *Nostoc*. She also studies the eco-evolutionary mechanisms and factors shaping interactions among lichen symbionts using peltigerous lichens as a model system. Her research integrates traditional specimen-based revisionary methods (morphology-, anatomy-, and chemistry-based approaches) and molecular phylogenetic tools, including genomic data, focusing on both the fungal and *Nostoc* partners. Jolanta is also part of the NSF-funded GoLife project exploring biodiversity, ecological factors, and biogeographical patterns in endolichenic and endophytic fungal communities associated with lichens and plants.

**Fig. 7:**
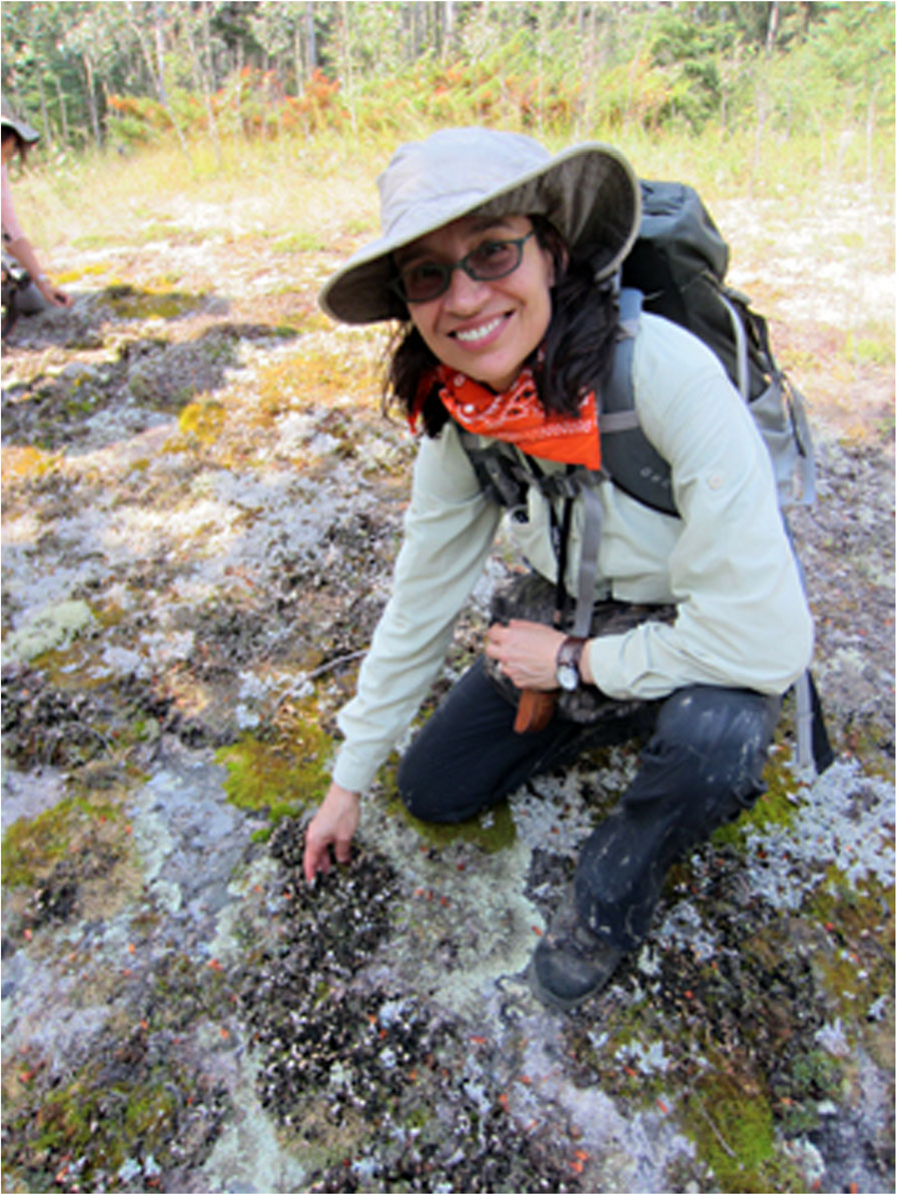
Jolanta Miadłikowska

**Pedro W. Crous**

(p.crous@wi.knaw.nl)

### State of the World’s Plants and Fungi 2020

(Fig. 8)

**Fig. 8:**
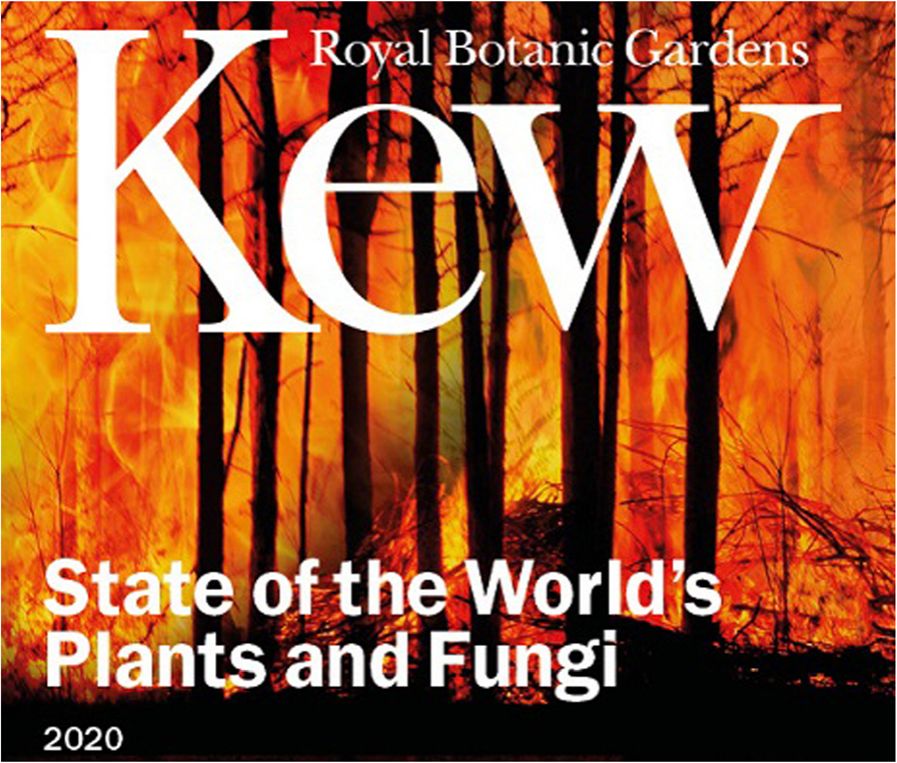
*State of the World’s Plants and Fungi* (2020)

The Royal Botanic Gardens Kew published reports on the *State of the World’s Plants* in 2017, and *State of the World’s Fungi* in 2018 (see *IMA Fungus* **9**: (48)–(49), 2018). These were both prepared through the collaboration of numerous specialists around the world and launched as a part of symposia organized at Kew, and that on fungi was of especial note as no similar report had ever appeared before. Now an update has been prepared considering both plants and fungi, the *State of the World’s Plants and Fungi* published on 30 September 2020 and available to download free of charge (https://www.kew.org/sites/default/files/2020-10/State%20of%20the%20Worlds%20Plants%20and%20Fungi%202020.pdf). There was no associated symposium due to the current pandemic, but the report was nevertheless very much international involving some 210 scientists from 97 institutions distributed through 42 countries. Treating plants and fungi together has the advantage of bringing botanists face-to-face with some comparative information on fungi. It was interesting to see that in 2019, while botanists described 1942 new plant species, the many fewer mycologists achieved almost as many at 1886 species; a most commendable achievement. Much is made of extinction risks, and some 600 plants are considered to have become extinct in modern times. No data are given for fungi here, but bearing in mind that on average a single plant species will support around six fungi that are restricted to it, the plant figure suggests we may have lost a staggering 6600 fungus species, most of which were almost certainly never formally named.

The major part of this report, however, concentrates on unlocking useful properties and the wise use of biological resources. Fungi are emphasized as sources of novel compounds, especially antibiotics, but while their value as foods themselves is hardly mentioned, they are recognized as having great potential in the production of bioenergy from woody plant materials. The ability of fungi to be grown in fermenters is also recognized for its sustainability, and their key mycorrhizal role is emphasized in relation to tree planting. Mycologists will endorse the case for more commercializing of nature-based products, but perhaps be more concerned over restrictions deriving from the Nagoya Protocol to the Convention on Biological Diversity than botanists seem to be. One difference from the previous reports is a section on the plants and fungi of the UK and its overseas territories; the total of UK native plant species is given as 2233 from data of the Botanical Society of the British Isles, which is higher than for some other lists due to how apomictic plants are treated. The number of UK fungi is said to be between 12,000–20,000 species, and no fungal data are provided for overseas territories.

Most mycologists will feel this report does not adequately address the state of the world’s fungi in 2020, but at least it will bring some data on the situation and importance of fungi to a wide botanical audience, who might thereby be encouraged to look at the 2018 report which concerns only fungi (see *IMA Fungus* **9**: (48)–(49), 2018).

### The impact of coronavirus on mycology

(Fig. 9)

**Fig. 9:**
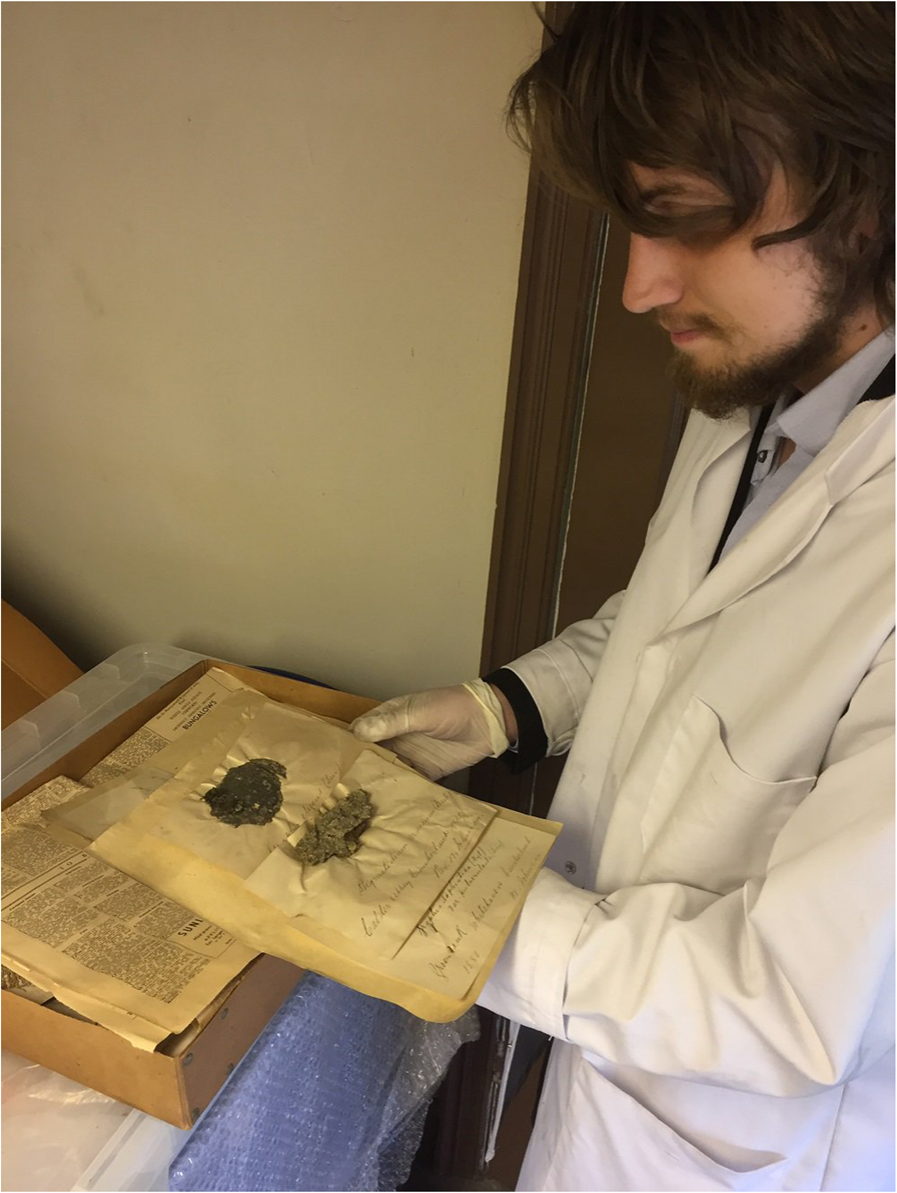
Nathan Smith

Coronavirus has had, and continues to have, severe and wide-ranging impacts across society. Unsurprisingly, this has included mycology. Indeed, whilst all scientific and taxonomic disciplines have felt an impact from the pandemic (e.g. see Baldini 2020 for considerations on how the pandemic has affected plant taxonomy), mycology has felt somewhat unique pressures due its organisational structure, less-institutionalised nature, and the demographic of its practitioners. Here, focusing on the practice of mycology within the context of the UK, I explore the effects coronavirus has already had on mycology, and indeed continues to have, as well as looking at how mycologists have adapted their practices.

Already, within less than a year, several key impacts are notable. Whilst much has been made of the narrative that ‘nature is returning’ (e.g. Manenti et al. 2020), little attention has been paid to the naturalists themselves. Indeed, lockdown has been found to have complex effects on bird recording in Africa, with a negative impact on more intensive recording projects but positive responses to more lockdown-friendly surveys (Rose et al. 2020). As regards mycology, in order to comply with government regulations and to protect the safety of participants, numerous fungal forays and recording expeditions have been cancelled throughout 2020. Whilst some individuals, and groups at a limited capacity, have maintained some recording activity, it is evident that surveying activity has reduced considerably across the country. Furthermore, within the context of the UK, the closure of the Royal Botanic Gardens, Kew, and the long-term furloughing of the majority of its mycological staff is also likely to have a severe impact on mycology in terms of identification and also long-term storage of specimens of note within the fungarium. These factors combined may mean that, in terms of mycological records and collections, 2020 is likely to be a somewhat underwhelming and unrepresentative year. Indeed, more so than any other event in recent history, the coronavirus pandemic has highlighted how wider societal factors and trends can influence sample collection and record-keeping. It has highlighted that we need to, in assessing historic records and potential longitudinal trends, take explicit account of such trends when analyzing data. This is particularly true for mycology where the harvest is great, and the labourers are few. Too often, fungal records depend on the work of a small number of unpaid individuals, and as such any period of inactivity by these individuals can have a substantial effect on regional and national mycological record quality and quantity.

The impact of coronavirus is also being felt in laboratory mycology and fungal science. Numerous laboratories were forced to close or significantly limit access to staff as a result of various lockdown procedures. The results of this are likely to be complex. For those with young children, the increased care responsibilities caused by school shutdowns is likely to have had a substantially deleterious effect on their work output — something that seems particularly true for women in academia (Flaherty 2020, Gabster et al. 2020). Furthermore, for those on fixed-term contracts, such as PhD students and early-career researchers, an additional strain has been placed on their research by the pandemic as periods of lockdown have eaten into valuable funding and lab time that may be unable to be reclaimed later. Effort must be taken to ensure that this does not impact their movement through the academic pipeline.

Finally, teaching, both formal and informal, is increasingly moving online for the foreseeable future. In a recent letter, Cota-Sánchez (2020) mused on the effectiveness of teaching plant taxonomy online. Issues such as limited screen view, size, and resolution of plant structures being discussed, and all students having access to the same plant material, are also applicable to the teaching of fungal taxonomy. However, the solution of Cota-Sánchez, of using virtual herbaria, provides a myriad of problems when applied to the teaching of fungi. Firstly, whilst the majority of plant herbarium specimens are dried and pressed on a page so as to present a near two-dimensional image of the plant structure, and thus relatively well suited to digital imaging and image analysis (Borges et al. 2020), the vast majority of fungal specimens (with the exception of particularly early-material) are dried in a method that maintains a three-dimensional structure and stored in packets, thus causing issues for useful analysis for fungal macrostructure from digital images. Secondly, and perhaps more importantly, is the essential use of microscopic characteristics and chemical tests in positively identifying fungal species. Whilst these may be resolved through the use of prepared slides and demonstrations, much will potentially be lost in students being unable to engage in practical work themselves. Indeed, the centrality of the microscope to mycology remains a substantial concern in adapting mycological teaching to the online environment.

**Fig. 10:**
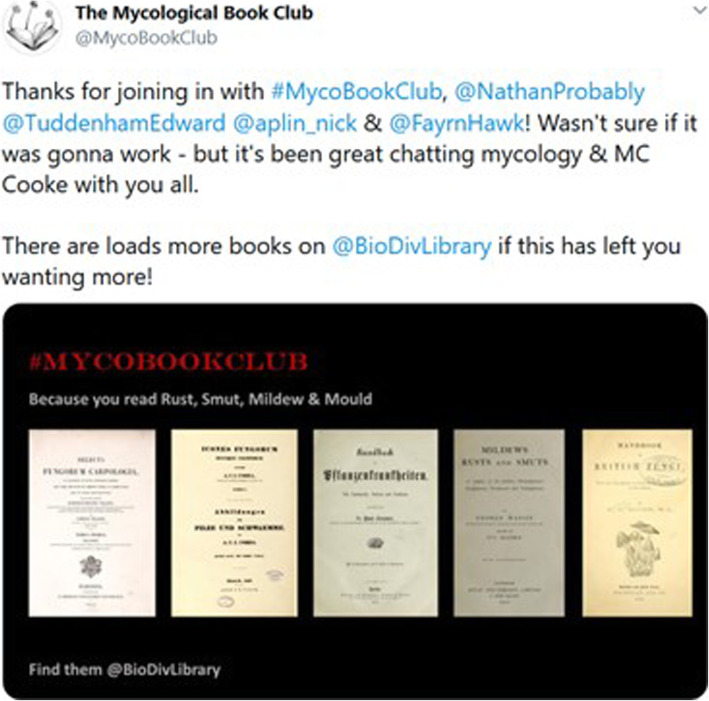
The Mycological Book Club web page, one of several independent projects started by UK mycologists during the first lockdown period

This is not to say mycologists have not adapted. In the UK alone, events have been translated into publications (Harries 2020) or transferred online, such as in the case of UK Fungus Day 2020 (see under Reports below) or British Mycological Society (BMS) Open Meetings, often reaching new audiences as a result. New publications (Diekonigin 2020) and communities (Blencowe 2020; Fig. 10) have also been developed, facilitating communication and a sense of belonging. Many local groups have moved their activities online, whether entirely or partially, allowing online identification and discussion to take place in a regional setting (Pinnington & Mynett 2020). Previously established resources, such as the BMS’s Facebook group, have become even more important, particularly for engaging and encouraging those new to the discipline.

As we enter into the second wave of the coronavirus pandemic, and renewed restrictions in the UK, it is perhaps a suitable time to consider the future of our discipline. There is much mycology has already achieved in these stressful and uncertain times. However, there is a need to consolidate those achievements so that firm foundations can be built for mycology in this new world we find ourselves in. For those seeking motivation, the most recent issue of the *BMS Newsletter* (2020(3)) contains reflections on organising UK Fungus Day during the pandemic alongside other responses of British mycology to the coronavirus pandemic. Inspiration may also be sought from adjacent disciplines, such as entomology, which have also undergone similar reflections on their practice (Coyle et al. 2020). Finally, whatever actions we take as a community, it is necessary to support mycologists across the amateur-professional continuum. The greatest asset to mycology has always been its participants and, as we continue to face new challenges brought forward by the pandemic, it is this asset that will enable us to survive, and perhaps even grow, as a discipline.

**Nathan Smith**

(ns565@cam.ac.uk)

### National approach to biodiversity: increasing knowledge together for The Netherlands

#### Centre of Excellence for Netherlands Biodiversity Research founded

(Fig. 11)

**Fig. 11:**
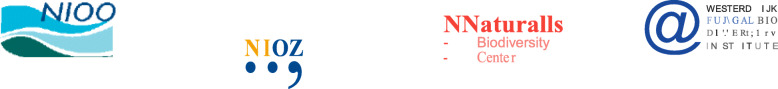
Partners in the Centre of Excellence for Netherlands Biodiversity Research

In order to increase knowledge about Dutch biodiversity, a national approach to scientific research is necessary. To make this possible, the Naturalis Biodiversity Center, The Netherlands Institute of Ecology (NIOO-KNAW), the Royal Netherlands Institute for Sea Research (NIOZ-NWO), and the Westerdijk Fungal Biodiversity Institute-KNAW are now joining forces. The aim is to work together to significantly increase integral knowledge of Dutch biodiversity in all environments: on land, in fresh and saltwater, and from genes to ecosystems. Together, the four institutes have launched the Centre of Excellence for Netherlands Biodiversity Research. The centre forms the core for cooperation with universities and knowledge institutes of The Netherlands. This creates a hub of knowledge about biodiversity that is available to every scientist.

This cooperation also builds on NWO’s recent investments in the National Roadmap for Large-Scale Scientific Infrastructure. Awards were made for the ARISE project (including Naturalis and the Westerdijk Institute) for the construction of a globally unique infrastructure to map all multi-celluular species within The Netherlands and for the project to provide NIOZ’s research fleet with innovative (large-scale) scientific equipment. Oret.nl is also a partner in the Onder het Maaiveld project, in which underground biodiversity is given the attention it deserves with a contribution from the Dutch Postcode Lottery.

These important investments now make it possible to jointly obtain a more reliable picture of biodiversity throughout the Kingdom of The Netherlands faster and smarter: The Netherlands, Aruba, Curaçao, and Sint Maarten.

**Pedro W. Crous**

(p.crous@wi.knaw.nl)

### ARISE: looking for invisible biodiversity

(Fig. 12)

**Fig. 12:**
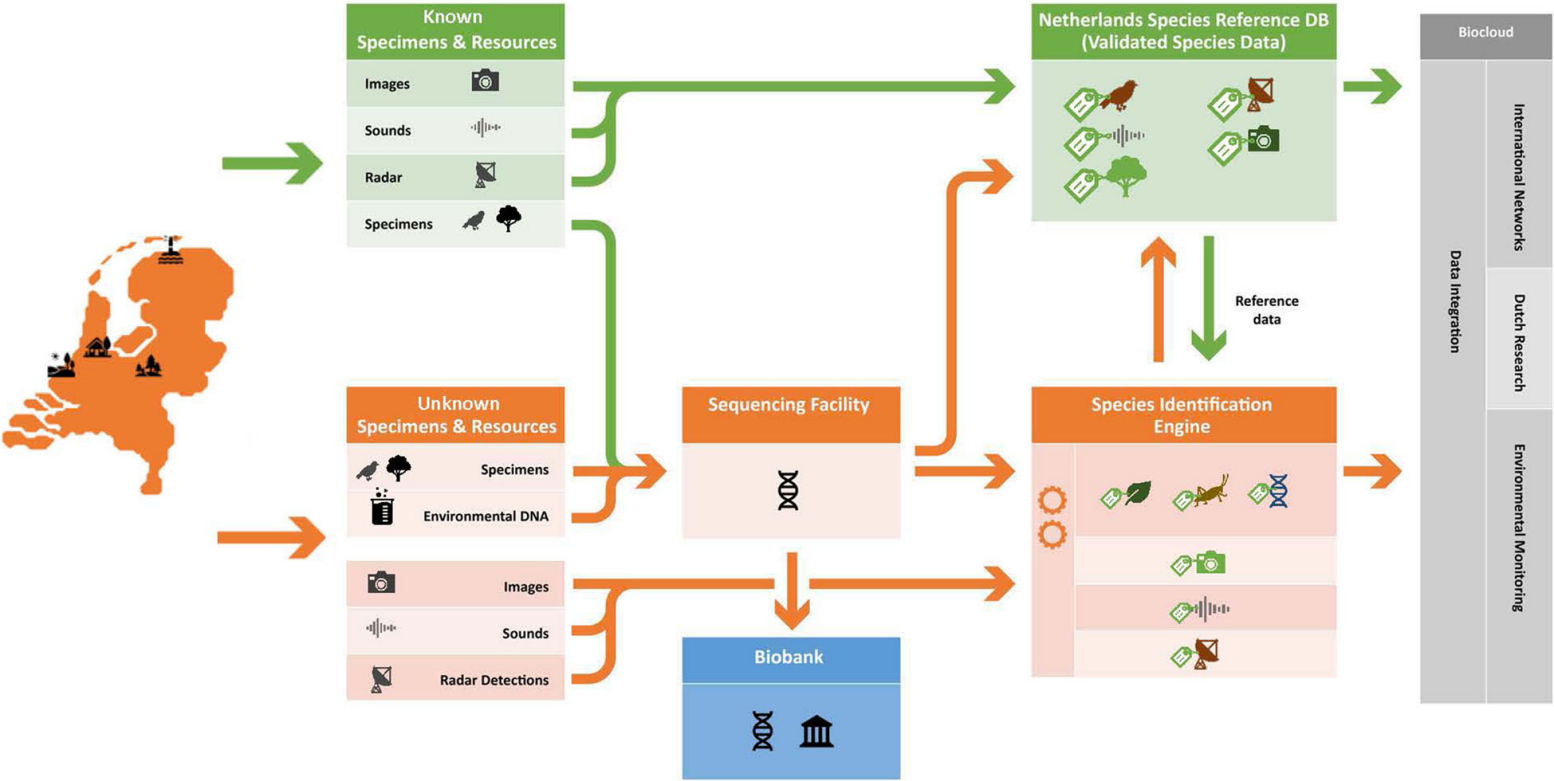
Data flow in the ARISE infrastructure

A new large scientific infrastructure for monitoring biodiversity and ecosystems (NIEBA-ARISE) has recently been funded in The Netherlands to the tune of 13.6 M€ by NWO (the Dutch Research Council). For Naturalis Biodiversity Center, University of Amsterdam-IBED, Westerdijk Fungal Biodiversity Institute, and University of Twente, the partners in the project, this is great news as it gives them the means to start building an infrastructure to identify all multicellular species within The Netherlands, and to provide the basis for broad scale, highly accurate and almost real-time monitoring of biodiversity. ARISE will be accessible to scientists worldwide and will make it possible to dive deeper into the big questions around biodiversity change, the structure of species interaction networks and the functioning of our ecosystems. It will also provide policymakers with more reliable information on biodiversity, giving rise to more effective measures to halt the loss of biodiversity.

The Westerdijk Fungal Biodiversity Institute will focus on soil biodiversity in The Netherlands. Within the CBS culture collection, there are more than 13,000 cultures isolated from ‘soil’ as substrate. These will be fully characterised based on morphology and DNA sequence, and the results made available to various online platforms. Furthermore, various monitoring projects throughout The Netherlands will provide additional soil samples from which more soil fungi will be isolated and added to the project. All Dutch insects, fungi, algae, and other small organisms will be mapped by using a combination of biodiversity knowledge and new techniques. The goal is to establish a reference database that can be used for research as well as for monitoring purposes. For further information see Biesmeijer et al. (2020).

**Pedro W. Crous**

(p.crous@wi.knaw.nl)

## REPORTS

### First International Symposium on Tropical African Mycology (FISTAM)

**Fig. 13:**
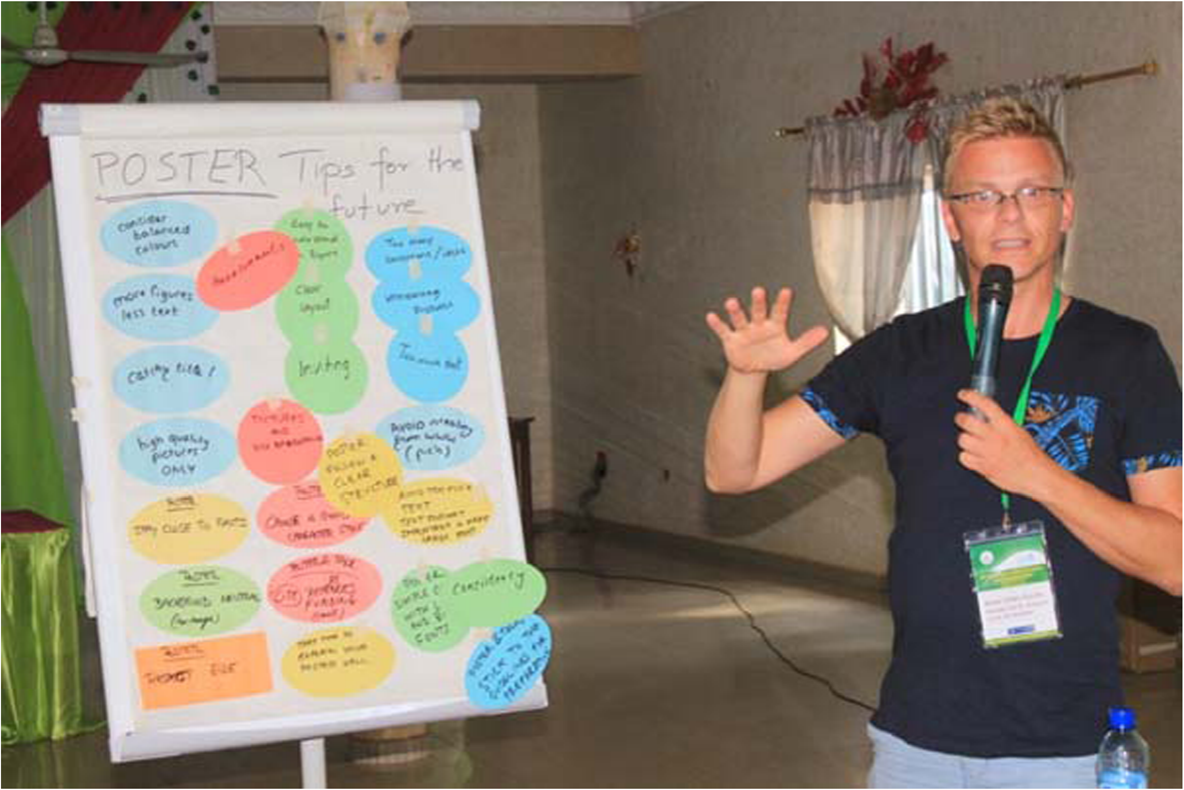
Training session on best presentation practices led by Michael Thomas-Poulsen (University of Copenhagen) on best presentation practices

**Fig. 14:**
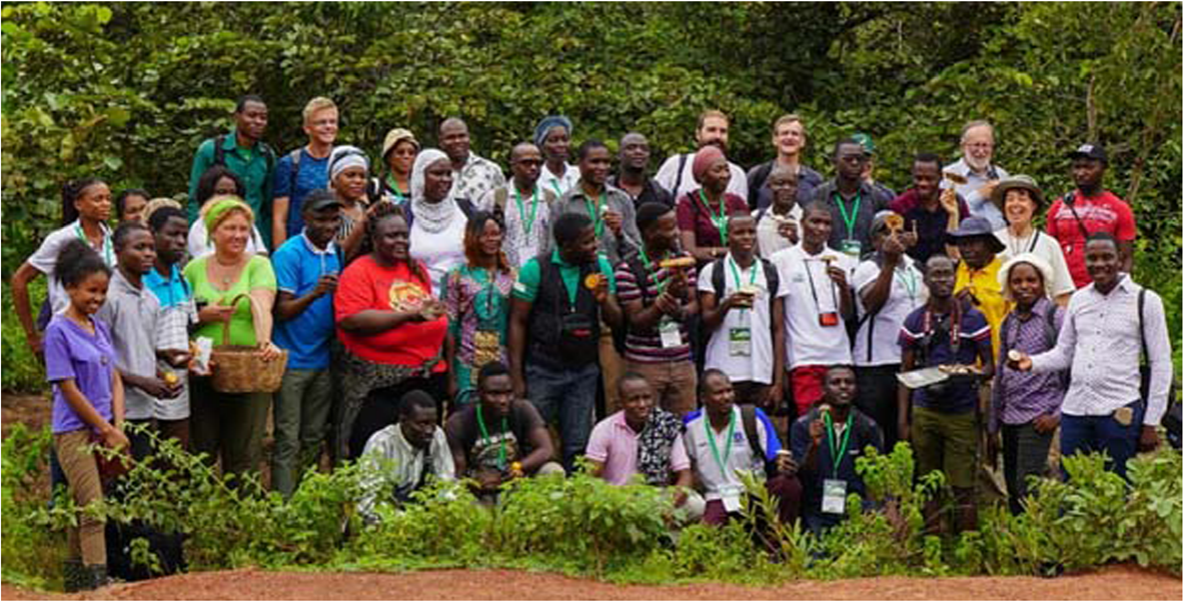
Group picture during the excursion at Okpara forest. Photo: André De Kesel

**Fig. 15:**
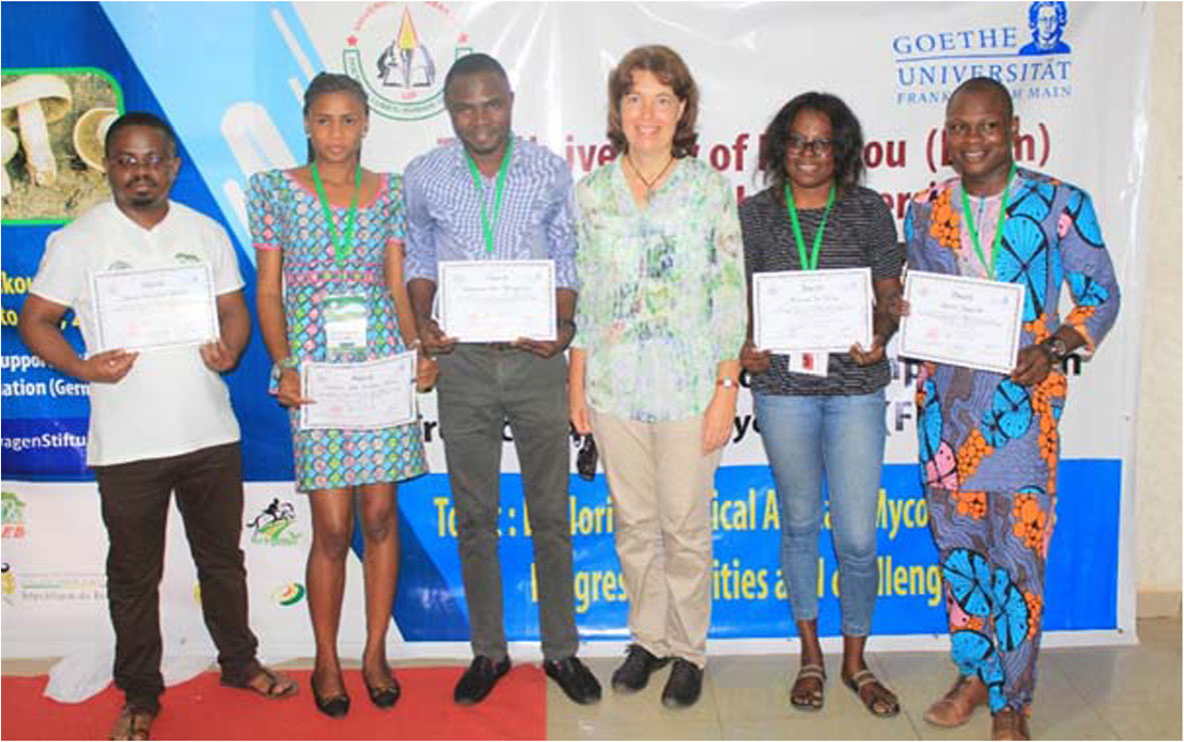
Young mycologist awardees: Héritier Milenge Kamalebo (DR Congo), Leontine Nicole Ematou Njiki (Cameroon), John Yangyuoru Kupagme (Ghana), Isis Gloria Mienandi (Congo), and Hyppolite Aignon (Benin) (*left to right*), with Meike Piepenbring (*centre*)

The First International Symposium on Tropical African Mycology (FISTAM) was held in the University of Parakou, Benin (West African), on 9–13 September 2019. The symposium welcomed 87 participants from 25 African and six European countries. The symposium covered a wide range of topics related to mycology and was divided into 11 sessions ranging from theoretical and fundamental aspects (taxonomy, molecular phylogeny, etc.) to applied fields (ethnomycology, cultivation of fungi, foodborne fungi and mycotoxins, etc.). In addition to 11 keynote talks by worldwide experts, 89 oral talks and posters were presented during the 5 days of the symposium. The oral talk sessions allowed young researchers (mostly PhD students and junior researchers) to learn about new research methods and to improve their capabilities in science communication. The poster sessions favoured interactions and discussions between young and more experienced mycologists. Many new research ideas came out from these discussions. Oral and poster presentations were supported by thematic training based on methodological limits depicted from oral talks by junior mycologists (Fig. 13). FISTAM was a success and has fostered the networking, establishment and encouragement of collaborations within African mycologists and between African and invited European mycologists. A one-day excursion (Fig. 14) to woodlands close to Parakou was used for the field demonstration of fungi belonging to diverse systematic groups and to facilitate personal interactions. To promote excellency in mycological research, five awards were distributed to the best oral and poster presentations by junior mycologists (Fig. 15). Finally, FISTAM was concluded by the ‘Parakou Declaration’ to promote mycology in tropical Africa.

The symposium was funded by the Volkswagen Foundation (grant n° 96–338) who are thanked for their invaluable support. We are also grateful to all other institutions which contributed to the realization of this symposium. Among others, the Faculty of Agronomy of the University of Parakou (Benin), the Goethe University Frankfurt am Main, the Centre for Interdisciplinary Research in Africa (ZIAF, Goethe University Frankfurt am Main), the municipality of Parakou, the Ministry of Higher Education and Research (MESRS) and the media service.

**Nourou Soulemane Yorou, Boris Armel Olou, and Meike Piepenbring**

(piepenbring@bio.uni-frankfurt.de)

### Recent advances in biodiversity, biology and biotechnology of fungi

(Figs. 16 and 17)

**Fig. 16:**
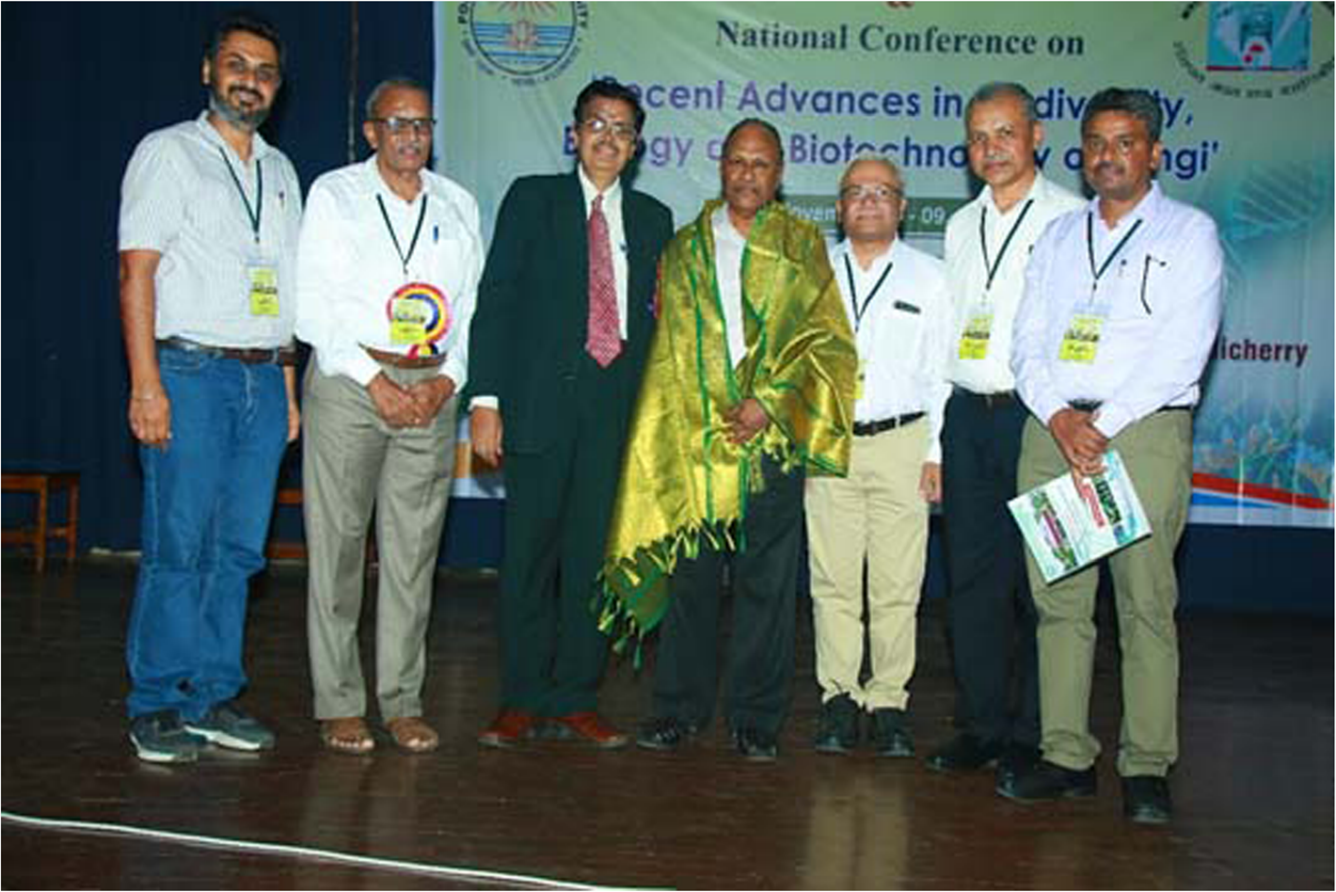
Chairperson of the first session and lead lecture presenters

**Fig. 17:**
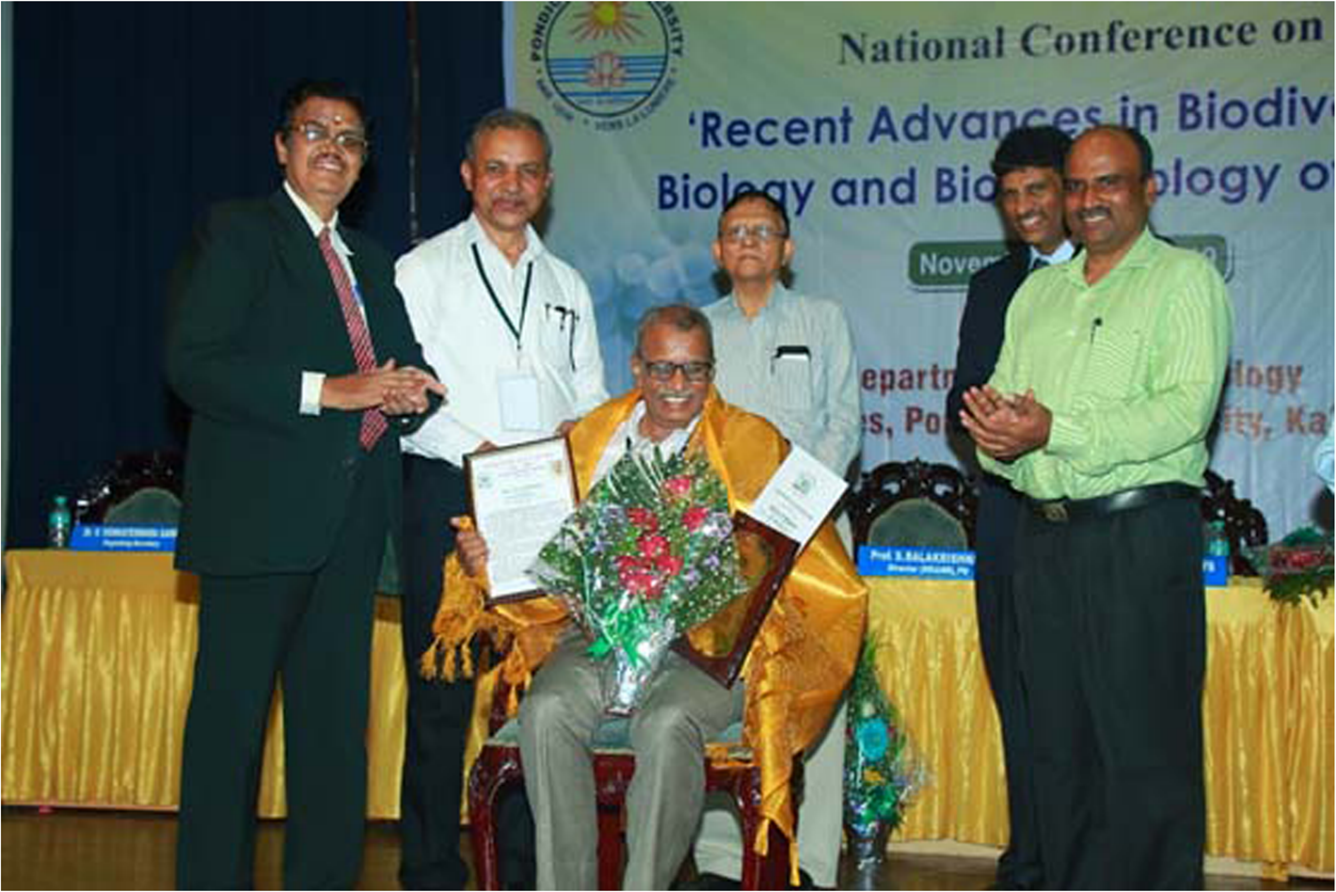
Life-time Achievement Award presented to K.R. Sridhar (*seated*) by the Chief Guest Shri Justin Mohan (*right*)

This three-day national conference was organized by the Department of Biotechnology, Pondicherry University from 7 - 9 November 2019 and held at the Convention-Cum-Convention (CCC) Centre of Pondicherry University under the auspices of the Mycological Society of India (MSI) which held its’ annual business meeting during the event. The conference was opened by Shri J. Justin Mohan (Indian Forest Service; Secretary, National Biodiversity of India, Chennai, India) who delivered an address on the importance of biodiversity and his connections with fungi, particularly VAMs. K.R. Sridhar (Mangalore University) received the MSIs Life-Time Achievement Award and lectured on the different dimensions and biodiversity of the aquatic microbiome. The first session started with a lecture delivered by MSI President N.S. Atri (Punjabi University) who shared his experiences in exploration, sociobiology, and conservation of mushrooms in India. Memorial award lectures included those for: (1) C.V. Subramanian delivered by S.J. Savitha (Bangalore University) on ‘*Coprinopsis cinerea,* a promising coprophilous fungus with novel therapeutic compounds’; (2) V. Agnihotrudu delivered by B.F. Rodriguez (Goa University) on ‘Arbuscular mycorrhizal fungi (AMF) – potential role as biofertilizers’; (3) K. Natarajan delivered by M. Sudhakara Reddy (Thapar University) on ‘The ectomycorrhizal fungi *Suillus*: diversity, plant growth promotion and metal tolerance mechanisms’; and (4) K. Shome delivered by Sanjay K. Singh (MACS Agharkar Research Institute, Pune) on ‘Studies on pathogenic and non-pathogenic isolates of *Fusarium oxysporium* to know their evolutionary lineages and its significance in agriculture’. This year’s special attraction was a session dedicated to the Indian Biological Diversity Act 2002 in which T. Narendiran (National Biodiversity Authority, Chennai) explained ‘Access and benefit sharing – law and procedures’. This was followed by a lecture on the ‘Fungal Endophytic Culture Collection of VINSTROM’ by T.S. Suryanarayanan (Vivekananda Institute of Tropical Mycology, Ramakrishna Mission Vidyapith, Chennai).

The main conference included nine technical sessions including ones on: The Biological Diversity Act 2002 and Culture Collections; Biodiversity, Conservation & Systematics of Fungi; Fungi in Agriculture and Forestry; Bioinformatics, Molecular Biology and Omics of Fungi; Biotechnological Applications of Fungi; Marine Fungi and their applications; Mushrooms, Food & Industrial Mycology; and Medical Mycology. Lead lectures included ones by D.J. Bhat (Goa University) on ‘Advances in identification and taxonomy of Fungi’; S. Raghukumar (MykoTech, Goa) on ‘Faces without identities, identities without faces: making sense of fungal speciation’; D.J. Bagyaraj (Center for Natural Biological Resources and Community Development, Bangalore) on the importance of ‘Arbuscular mycorrhizal fungi for sustainable agriculture’; N. Mathivanan (University of Madras) on ‘Bioprospecting *Trichoderma* for biological control of plant diseases’; Manchikatla V. Rajam (Delhi University) on ‘RNA interference mediated silencing of the important genes of *Fusarium oxysporum* for resistance against Fusarium wilt in tomato’; Marcio Jose Pocas (University of Brazilia) on ‘Evaluation of the interaction amongst antifungal drugs, epigenetic modulators and phododyamic therapy in *Cryptococus neoformans*’; T. Satyanarayana (formerly Delhi University) on ‘Attempts in unravelling potential applications of the thermophilic mould *Sporotrichum thermophile*’; R.N. Kharwar (Varanasi) on ‘Fungal endophytes: an untapped resource for cryptic biodiversity and bioactive compounds’; Rajesh Jeewon (University of Mauritius) on ‘Marine fungi: morpho-molecular taxonomic evaluation and species diversity based on DNA sequence data’; Yashpal Sharma (University of Jammu) on ‘A decade of understanding ethnomycology of wild edible mushrooms in Jammu and Kashmir’; Kalaiselvam (Annamalai University) on ‘Current status, challenges and opportunities in marine mycology’; and K.K. Janardhanan (Amla Cancer Research Centre, Kerala) on the ‘Development of novel anticancer bioactive compounds from mushroom mycelia by biotechnological process’. A preconference workshop on ‘Methods to study marine fungi’ was held on 6 November 2019.

In addition Naveen Kango (D. Harisingh Gour Vishwavidyalaya, Saagar) was chosen as an Associate Fellow of Mycological Society of India and spoke on ‘Cellulases and hemicellulases of *Aspergilus tubingensis*: applications in sugarcane bagasse and rice straw valorization’. In total there were 18 lead lectures, 4 memorial award lectures, 67 oral presentations, 55 poster presentations, one life-time achievement award lecture and a Presidential lecture. Around 150 abstracts were received, and the conference had over 230 participants including three from other countries. The conference was concluded with a valedictory function, during which Thirumulachar Best Oral and Poster presentations were made.

**V. Venkateswara Sarma**

*Organizing Secretary* (Pondicherry University)

(sarmavv@yahoo.com)

### 4th International *Malassezia* Workshop

(Fig. 18)

**Fig. 18:**
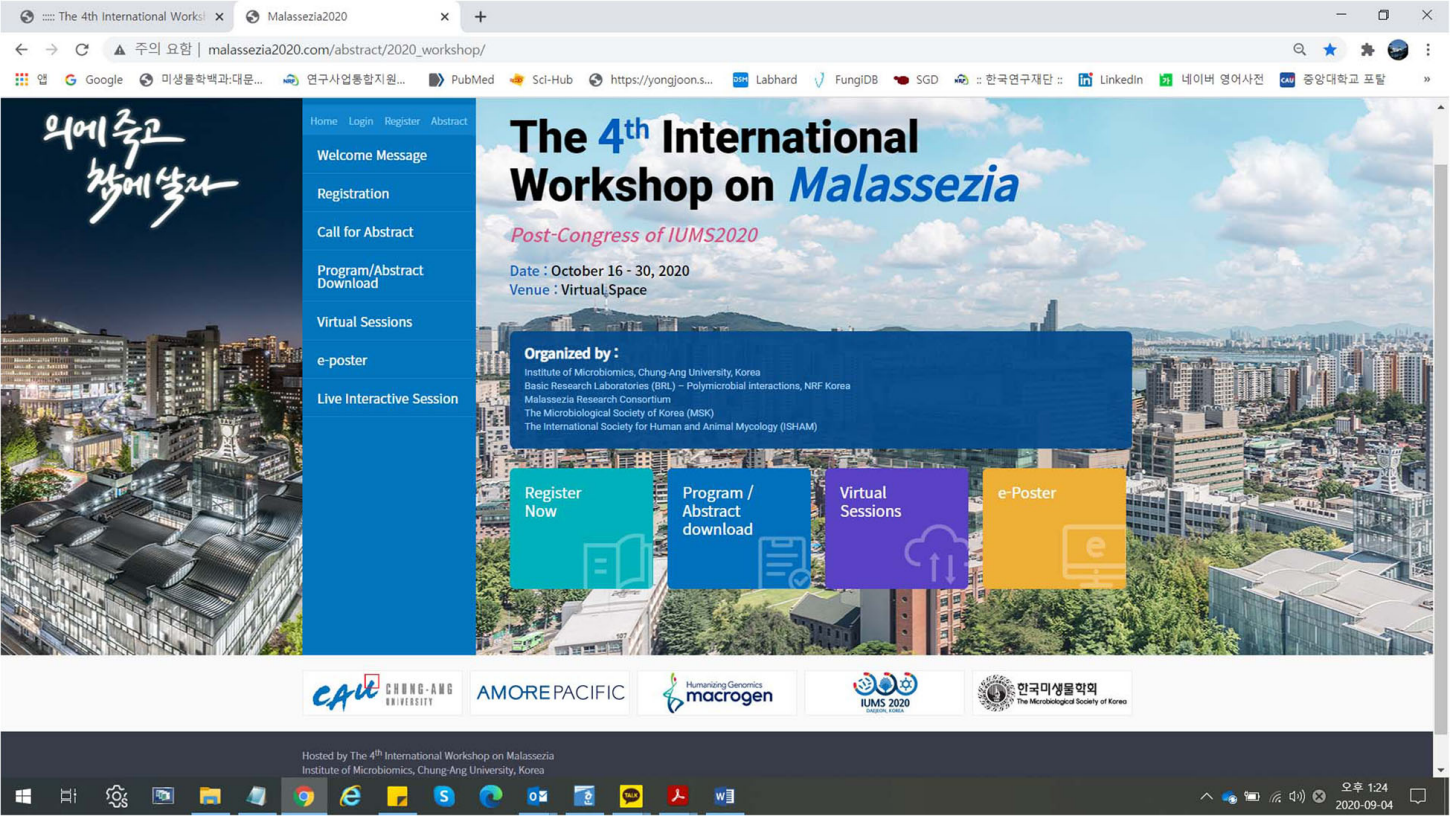
Website of the 4th International *Malassezia* Workshop

This was organized as a virtual meeting and held on 16–31 October 2020 on behalf of the ISHAM working group on *Malassezia* and the International *Malassezia* Research Consortium. The presentations were organized into five themes: (1) Ecology and Diversity; (2) Clinical, therapeutics, and diagnostics; (3) Human disease and implications; (4) Microbiome, host interactions, immunology, and biochemistry; and (5) Molecular biology, genetics, and genomics. The meeting had 240 participants from 37 countries and the website of the workshop was visited 4134 times.

All talks were pre-recorded, and the participants had access to them during the entire meeting. One lecture stood out in number of views, namely that on the ecology of *Malassezia* yeasts by Anthony Amend (Hawaii) with around 250 views.

Although this workshop was initially planned as a regular meeting, all participants were happy with this virtual version, thanks to the local Korean organizers. A future workshop is planned for 2022 linked to the ISHAM congress in New Delhi.

**Teun Boekhout, Tom Dawson, and Wonhee Jung**

(*Organizers*)

(t.boekhout@wi.knaw.nl)

### Fungi to dye for – Dutch Design Week (DDW)

(Fig. 19)

**Fig. 19:**
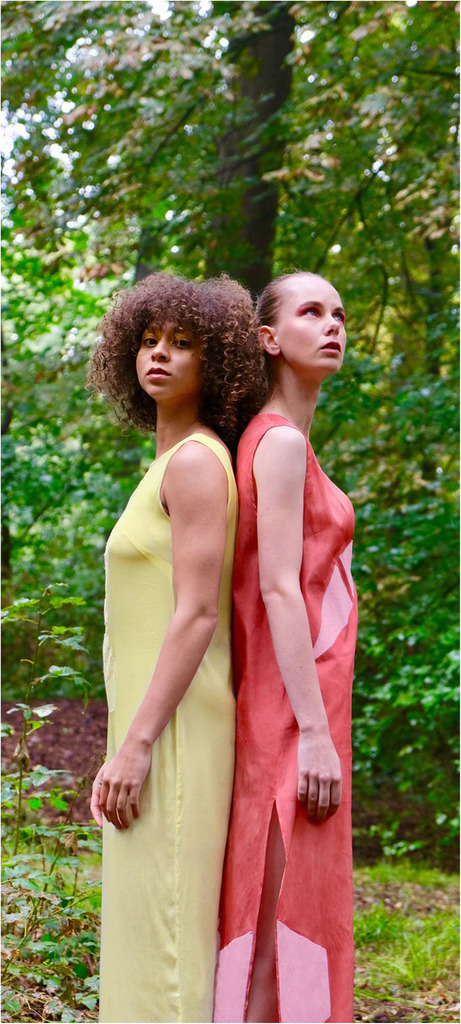
Dresses designed and coloured by Ilse Kremer using fungal dyes

One of the major problems in the fashion industry today is water pollution caused by harmful textile dyes. Designer Ilse Kremer therefore developed a project which focusses on how biodesign can reduce the use of such dyes by employing fungi. Together with myself, as group leader for Fungal Natural Products at the Westerdijk Fungal Biodiversity Institute, Ilse created a special fungal dye. The Figure icludes an example of the clothes she stained with a yellow compound we produced for her. The project was presented during Dutch Design Week which ran on 17–25 October 2020. The event was held fully online this year, so the designer setup a virtual room for this and also did a livestream during the weekend in which she discussed her work and showed garments she had made.

For more about this project see https://ddw.nl/en/programme/3241/fabulous-fungi or check out Ilse Kremer’s personal webpage (http://www.ilsekremer.nl/fabulous-fungi.html) for even more examples of her beautifully dyed fabrics.

**Jérôme Collemare**

(j.collemare@wi.knaw.nl)

### UK Fungus Day 2020 – a virtual event under COVID-19 restrictions

(Fig. 20)

**Fig. 20:**
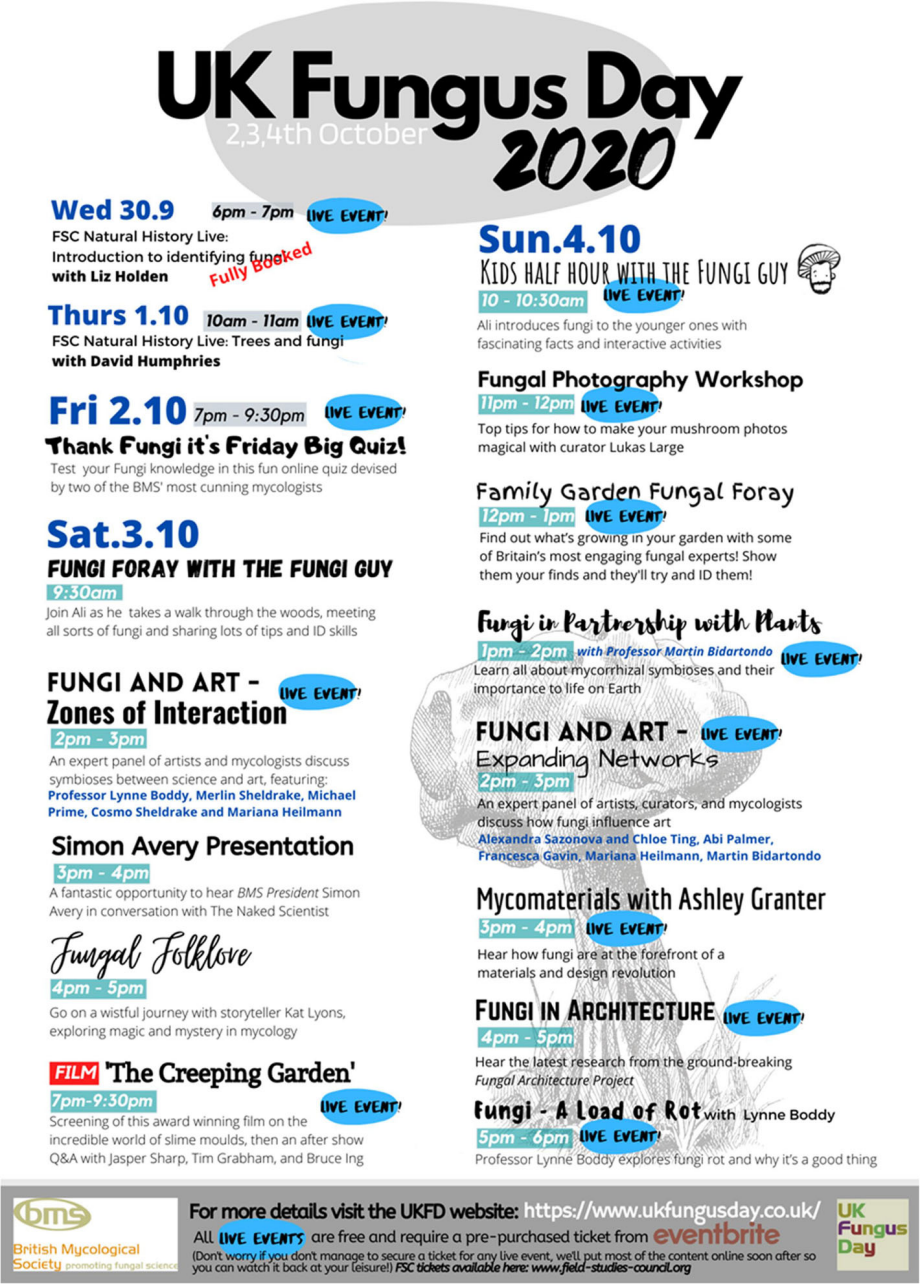
UK Fungus Day poster with virtual events indicated

The decision to move UK Fungus Day (UKFD) online was made by the British Mycological Society (BMS) in April when the impact of the pandemic was reaching its first peak. October 2020 seemed a long way off then, but memories of the time commitment and hard work from public-facing events in 2019 were still fresh, so a virtual UK Fungus Day sounded like an easy option. It turned out to be every bit as challenging for the small team involved.

The Competition tab provided the ideal layout for promoting this year’s challenge, ‘*Create a Fantasy Fungus’*, and Ali MacKernan who led this initiative, designed and illustrated the content. Creating an online application form was another new hurdle successfully overcome. Rich Wright created a new logo for the site and persuaded a wide range of artists to share their interests in mycology by contributing to UKFD virtual events.

It was agreed that most of the communication and messaging would go out through Facebook to organizations and individuals who regularly follow UKFD through those pages. Several committee members also contacted external organizations such as the Field Studies Council and Royal Society of Biology who all were keen to support UKFD. Closer to the launch, an interactive page was designed where visitors could sign up for their favourite events using Eventbrite software. Everything worked well, and most events were gratifyingly over-subscribed.

There are many steps that can be taken to increase inclusivity and diversity within the BMS membership, and UKFD provides a unique opportunity to explore and promote some of these in the future. If the Society is to appeal to a wider audience, it must consider new and different ways of interacting with the public such as those tried this year. This issue was raised at BMS Council recently and the Fungal Education & Outreach Committee will now explore how best to move this agenda forward.

**Norman Porritt**

(norman@britmycolsoc.info)

Correction In the report of the 18th Congress of European Mycologists (*IMA Fungus* **10**(23): 4, 2019), the caption of Fig. 4, should be modified to: ‘Some members of the CEM local organizing committee: Julia Pawłowksa (*left*), Marta Wrzosek (*centre*), and Magdalena Frąc (*right*)’.

## AWARD

### Francis Martin – Légion d’ Honneur

(Fig. 21)

**Fig. 21:**
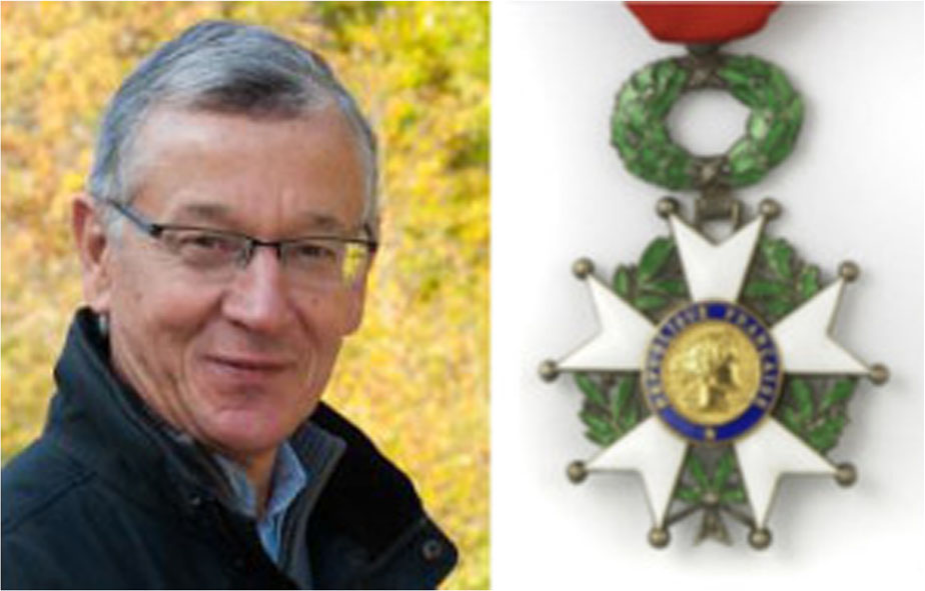
Francis Martin and the award’s medal

The IMA is pleased to congratulate Francis Martin, who since 2010 has been Director of Research in the Advanced Research on the Biology of Tree Biology and Forest Ecosystems (ABRE) unit within the Institut National de la Recherche Agronomique (INRA) in France, on his appointment in the rank of Chevalier of the prestigious French Legion d’Honneur. Francis is well-known for his outstanding genomic studies of mycorrhizal and other fungi, including ecto- and endomycorrhizas, signalling pathways, and further the origin and the loss of lignin-decomposition mechanisms during the evolution of basidiomycetes. His name will also be familiar to many readers for his role in various editorial capacities for several leading journals, including *Fungal Biology Reviews, Fungal Genetics and Biology, Mycological Progress,* and *New Phytologist.* It is always a pleasure to see distinguished mycologists recognized by the broader scientific community, and we trust that this recognition will help facilitate Martin’s future explorations of the genomic basis of fungal life-styles and symbiotic interactions with other organisms.

## BIRTHDAY GREETINGS

### John Walker – a mycologist for Australia

(Figs. 22 and 23)

**Fig. 22:**
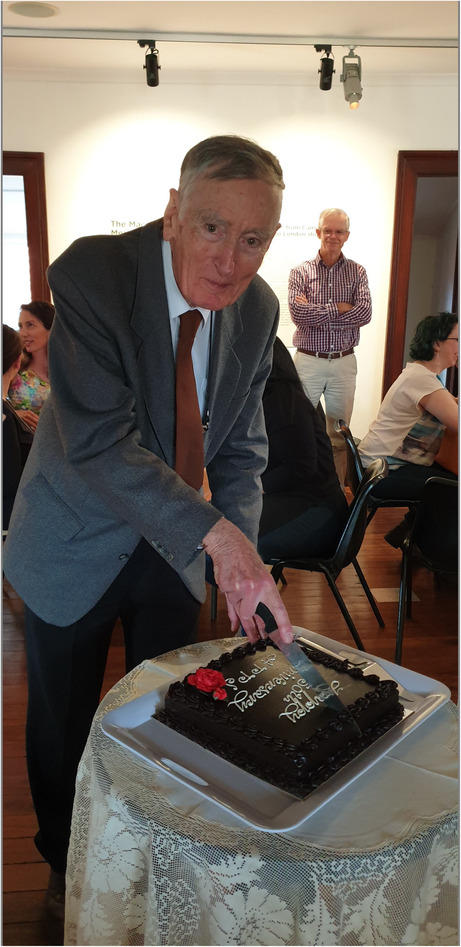
John Walker at the Australian Plant Pathological Society (APPS) 50th anniversary celebration. Belgenny Farm, 2019

**Fig. 23:**
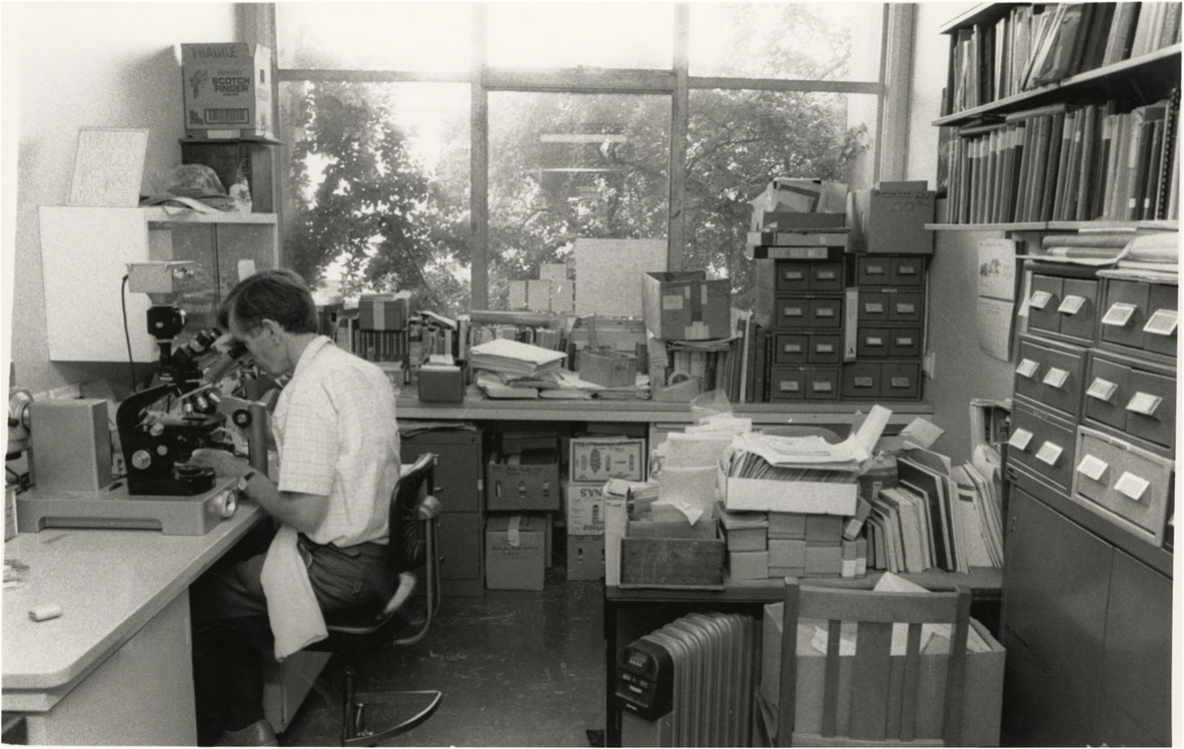
John Walker in his office at the Biological and Chemical Research Laboratory, Rydalmere, 1988

John Walker (b. 1930), who celebrated his 90th birthday this year, has shaped mycology and mycologists in Australia for over 60 years. John joined the Department of Agriculture in New South Wales in 1952 and became the curator of the Plant Pathology and Mycology Herbarium (DAR) at Rydalmere (Sydney) in 1960. John has diverse mycological interests, making many taxonomic contributions to the study of Australian plant pathogens, both ascomycetes and basidiomycetes, particularly rusts and smuts.

A remarkable aspect of John’s enduring career has been the breadth and depth of his taxonomic skills, particularly in the pre-molecular era. John introduced, sometimes with colleagues, almost 100 new fungal species and seven new genera, namely *Arkoola* (1986), *Cepsiclava* (2004), *Kirramyces* (1992), *Lidophia* (1974), *Polythrinciopsis* (1966), *Racospermyces* (2001), *Sonderhenia* (1988), and *Yelsemia* (2001); the last a touching dedication to his late wife Elsie May. John delivered the Daniel McAlpine Memorial Lecture twice (in 1980 and 1991), an honour extended biennially by the Australasian Plant Pathology Society to eminent scientists in recognition of their significant contributions. In his addresses, John highlighted many issues that still challenge Australian plant pathologists today — the under collection of fungal specimens for study and future reference; neglect of diseases of native plants and weeds; increased specialisation of plant pathologists at the crop, pathogen or discipline level; lack of knowledge about the biology of many plant pathogens; and the reliance of effective biosecurity (quarantine) on accurate identification of plant pathogens. John also embodies Daniel McAlpine’s (1894–1932) desire that Australian mycologists should be independent of their European counterparts. When John started his career, most specimens of fungi collected in Australia were sent to the former Commonwealth (later International) Mycological Institute in Kew. By the end of John’s professional career DAR housed, and still does, the largest collection of fungi in Australia.

Australian mycologists and plant pathologists have held John in high esteem for over 50 years. This is reflected in the etymology for *Vermisporium walkeri*, penned by Haring (‘Harry’) Swart (1922–1993) and M. Anna Williamson in 1983, for his struggle to gain full recognition for the efforts of taxonomic mycologists in Australia.” Other fungal names that honour John include *Acrocalymma walkeri* (as *Massarina walkeri*), *Anthracocystis walkeri* (as *Sporisorium walkeri*)*, Endoraecium walkerianum*, *Gaeumannomyces walkeri*, *Mycosphaerella walkeri*, and *Tilletia walkeri*.

John was more than a curator to DAR, but is a mycologist for Australia, who champions the value of collections for biodiversity and biosecurity purposes. His recently published historical account of DAR and its work (Walker 2018) provides a glimpse of his passion:

“Professor W. Sackston from the University of Montreal was an expert on diseases of flax, and had come to investigate work on flax and linseed diseases being done in Australia. We’d been warned of his visit a day or two before and exhorted by Dr Magee to make sure our rooms were tidy. As usual, I left my room just as it always was but around the Branch, there was a flurry of activity as everyone cleaned up their room, things were put away in cupboards, benches were neat and clean with only the microscopes standing on them, on their desks the papers were arranged in piles, their clean lab coats were unsullied white and, on the day, many of the occupants were sitting at their desks writing. As was his habit, Dr Magee brought the visitor around the Branch himself and when they came to my room, I was at the bench looking at a slide under the microscope, my white lab coat somewhat less than pristine, and surrounded by my usual mess of specimens and papers. Dr Magee introduced me to Professor Sackston and we spoke for several minutes about diseases of flax and linseed in Australia. Over the years, I’d had quite a bit of experience with these crops and he was pleased to hear about the disease situation in New South Wales. As we finished talking, he turned to Dr Magee and said ‘*Do you know, Charles, this is the first room I’ve come to that looks as though there’s some work being done’*. Dr Magee looked a bit abashed, I can’t remember whether he said anything or not, I just stood there looking blank but inside I leapt for joy and thought “Good on you”. I must say I never understood the attitude that we must put on an act for visitors, when it was far preferable we just be ourselves and get on with our work as usual.”

We send John our best wishes on the occasion of this special birthday, and thank him for all he has done for Australian mycology with such distinction over such an extend period.

**Jordan Bailey, Michael Priest, and Roger Shivas**

(roger.shivas@daf.qld.gov.au)

### José Carmine Dianese – explorer of Brazil’s Cerrado microfungi

(Fig. 24)

**Fig. 24:**
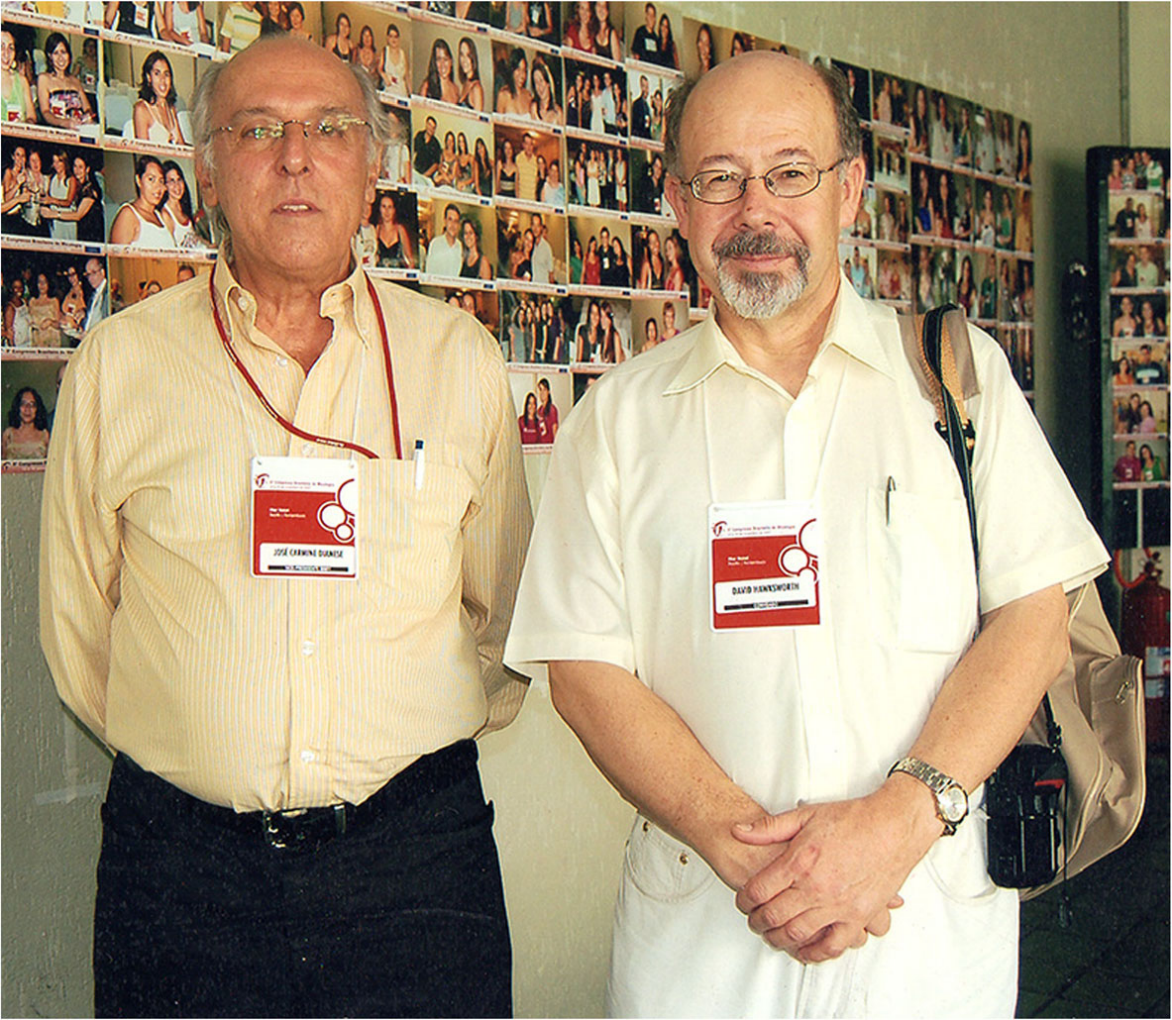
José Carmine Dianese (*left*) and the author (*right*) at the 5th Congresso Brasiliero de Micologia, Recife, 2007

José, born in Santo Antonio do Monte, State of Minas Gerais, Brazil on 9 May 1940, right in the centre of the Cerrado, turned 80 this year. Compulsorily retired in 2010 on his 70th birthday, for 44 years he was linked to the Biological Sciences Institute of the University of Brasília (UnB), following his return from the University of California-Davis (UC-DAVIS) in 1970 from which he obtained a PhD in phytopathology. He was Director of the Institute for 1972–1978, where he established a Department of Plant Pathology, and later a mycological collection (UB) dedicated to Cerrado fungi, and now impressively with some 25,000 specimens. The Department had a major educational role, graduating over 300 postgraduates from Brazil, other Latin American countries, and Africa. He personally supervised around 40 master and doctoral degrees, of which 15 recipients later became professors in Brazilian universities.

His roughly 130 publications have made an unparalleled contribution to our knowledge of fungi in the Cerrado, including the description of over 120 new species and more than 20 new genera from this extraordinary biome, and these studies continue today. The collections accumulated from the region are a unique resource and record of fungi in an area rich in endemic plants and animals but which is also subject to fires and extensive human disturbance.

José served as President of the Latin American Mycological Association (ALM) for 2002–05, and chaired the VI Latin American Congress; he was elected an Honorary Member of the ALM in 2008, and continues to play an important role on the world mycological stage – through both invited lectures at meetings around the globe, and more particularly as a member of the Executive Committee of the IMA, International Committee on the Taxonomy of Fungi (ICTF), and the Nomenclature Committee for Fungi (NCF). His contributions have been widely recognized, and he is the only Brazilian to be elected an Honorary Member of the Mycological Society of America, and is a Fellow of the American Phytopathological Society, and recipient of both the Álvaro Santos Costa Award of the Sociedad Brasileira de Fitopatologia, and the Mrak International Award from the University of California (Davis).

The IMA wishes him all the best of health in this special birthday year, and looks forward to eventually seeing a synthesis of his work on the Cerrado fungi in print!

### Richard “Dick” C. Harris – American lichen taxonomist turns 80

(Fig. 25)

**Fig. 25:**
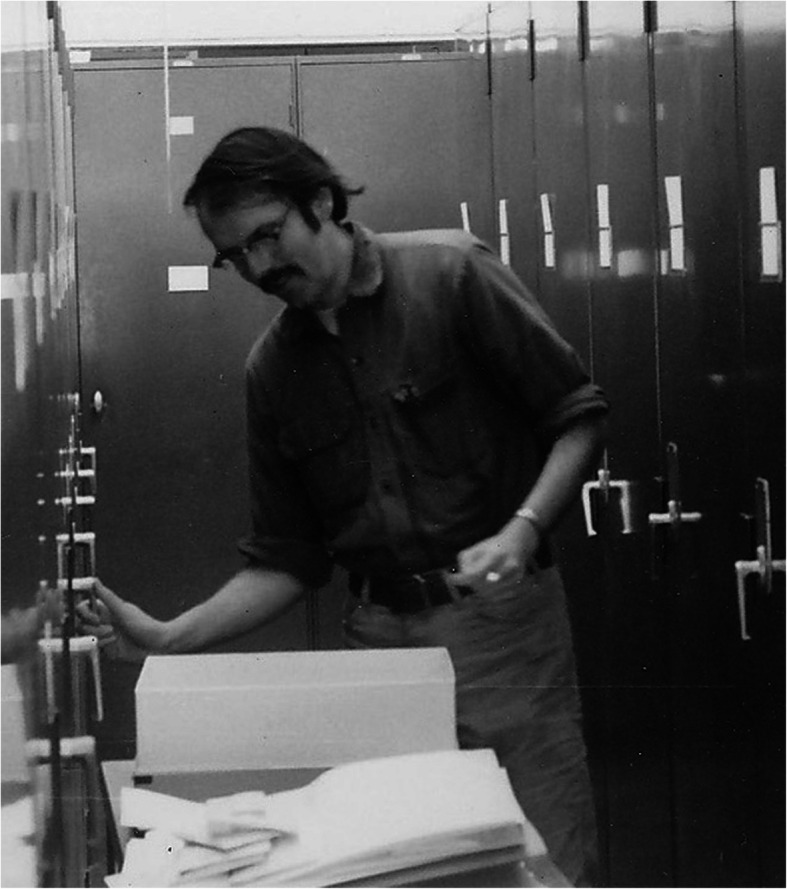
Richard C. Harris at work in NYBG

Somewhat belatedly, we wish Dick all the best for turning 80 on 7 December 2019. Dick is a well-known member of the lichenological community whose published work spans more than half a century (1966–on). He joined The New York Botanical Garden in 1979, and commenced a research program me documenting New World lichen biodiversity, especially that of the eastern USA and Caribbean. Although his taxonomic focus was always on pyrenolichens, his interests were general and resulted in publications that touched on nearly every lichenized group.

At a time when crustose lichens were particularly understudied in temperate and tropical areas of the New World, Dick wrote and published some of the first general identification keys for these regions in modern times. In addition to published work, Dick was one of the founders of the Tuckerman Workshop series, which has brought together professionals, amateurs and students for nearly 30 years. The connections made through the Tuckerman, coupled with Dick’s tireless support of beginning students (including myself), built a network of lichen specialists in a region that much needed increased scientific capacity. These efforts coincided with a mammoth expansion of the lichen colletions at The New York Botanical Garden. Over more than 30 years, Dick transformed a small collection with historically important specimens into one the largest and most active lichen collections in the world. Rather than diffuse growth of the holdings, Dick focused and in doing so created the most comprehensive possible resource for the lichens of the regions he knows and loves.

For a more detailed biographical sketch, refer to Buck (2009), who also provided a review of the achievements of the Tuckerman Workshop (Buck 2016). A review of the history of the New York Botanical Garden lichen collection was published by Lendemer & Harris (2016).

**John Lendemer**

(jlendemer@nybg.org)

### Tomasz Majewski – doyen of the *Laboulbeniomycetes*

(Fig. 26)

**Fig. 26:**
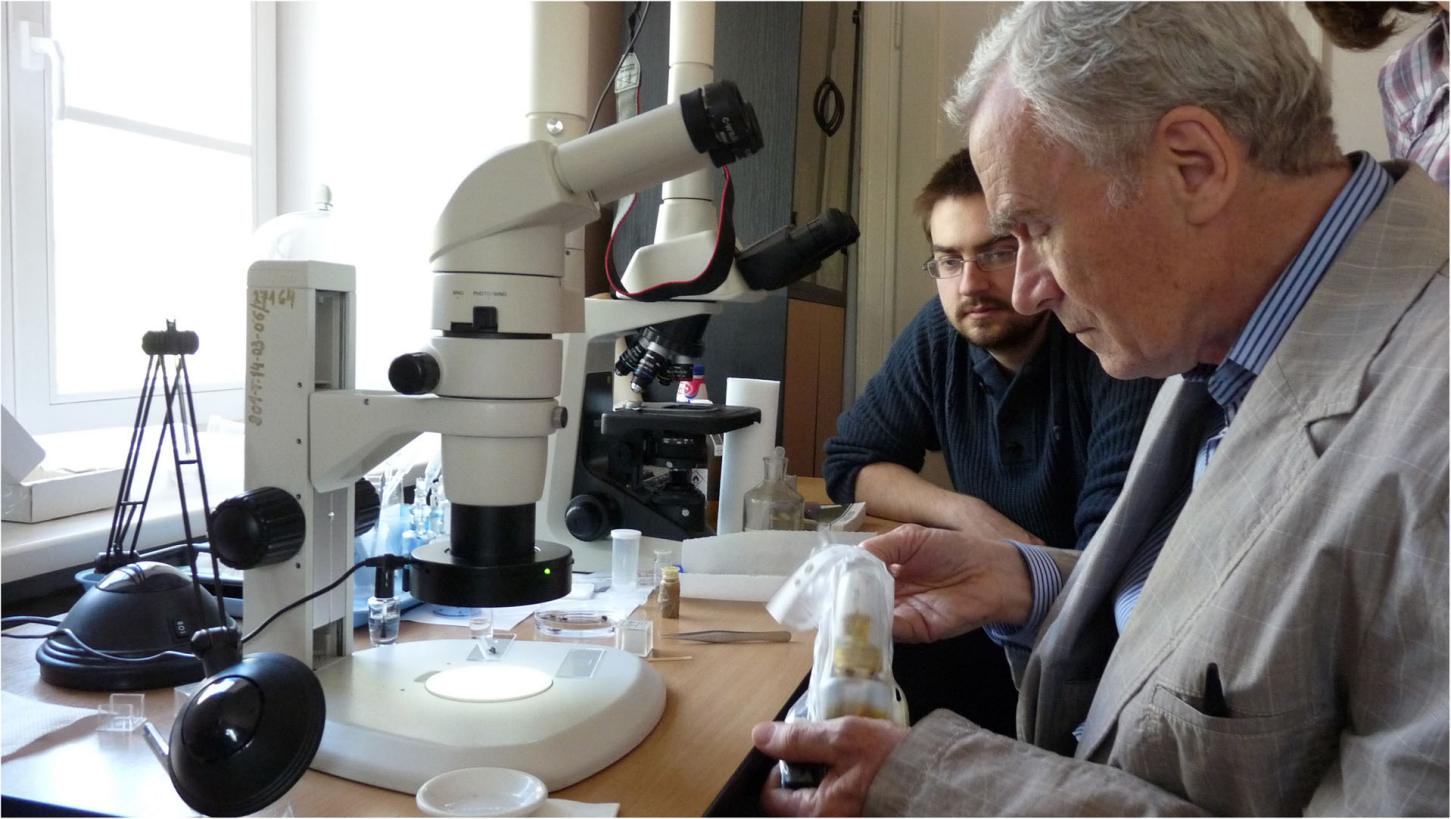
Tomasz Majewski teaching. Photo: Marta Wrzosek

Tomasz Majewski celebrated his 80th birthday on 28 July 2020. Born in Warsaw, he obtained an MSc from the University of Warsaw in 1963. He was working at the Phytopathology Department of the Warsaw University of Life Sciences in Józef Kochman’s group, and defended his PhD thesis on *Parasitic fungi of the Białowieża National Park against the background of the mycoflora of Poland* in 1969. Although employed for 25 years by the Institute of Botany of the Polish Academy of Sciences in Kraków, his place of work remained at the Department of Plant Systematics and Geography in the University of Warsaw. In the 1980s, Tomasz Majewski spent a one-year scientific fellowship at Shizuoka University, Japan, working on the biodiversity and taxonomy of *Laboulbeniales*. He obtained the title of full professor in 1988, and in 1990 returned to the Phytopathology Department of the Warsaw University of Life Sciences, retiring in October 2006.

During his scientific life, Tomasz focused mainly on microfungi with a special emphasis on obligatory parasites of plants and arthropods. Starting from the early 1970s, he gathered one of the world’s richest collections of Laboulbeniomycetes, stored at the Institute of Botany of the Polish Academy of Sciences (KRAM). He described five new genera and almost a hundred new fungal species in works beautifully illustrated by line drawing, and amounting to more than 120 papers and monographs, among which the most cited are: *The Laboulbeniales of Poland, Distribution and ecology of Laboulbeniales in the Białowieża Forest*, *The Atlas of the Geographical Dstribution of Fungi in Poland: Laboulbeniales,* and *A preliminary Checklist of Micromycetes in Poland*. Thanks to his thorough research on *Laboulbeniales* carried on for 40 years, Poland is today the best studied country for these fungi. Amongst his earlier well-known monographs are four Polish books devoted to obligatory plant parasites (*Peronosporales* s. lat., *Ustilaginales* s. lat., and *Pucciniales*). He also made a large contribution to the development of the history of science, writing biographical notes on famous Polish botanists, phytopathologists, and mycologists, and two extensive books presenting a history of botany in Warsaw and a history of understanding plant diseases in Poland. He is still active in this field today, and currently working on a book on the history of Polish mycology which we look forward to being realized. A detailed cv, together with a list of species described by him and named in his honour, was published by Mułenko & Ruszkiewicz-Michalska (2010).

Tomasz is a member of the Polish Botanical Society, Polish Phytopathological Society, and the Warsaw Scientific Society. In 1995 he was awarded the Professor Władysław Szafer’s Medal for his outstanding scientific achievements, and he has been awarded Honorary Memberships of the Polish Botanical Society and Polish Mycological Society.

We thank him for his guidance in mycology for all this time, and we send him our congratulations and best wishes on the occasion of this special birthday.

**Julia Pawłowska, Michał Gorczak, Marta Wrzosek, and Małgorzata Ruszkiewicz-Michalska**

(jzpawlowska@gmail.com)

### David “Dave” Warren Malloch – one of Canada’s most influential mycologists

(Fig. 27)

**Fig. 27:**
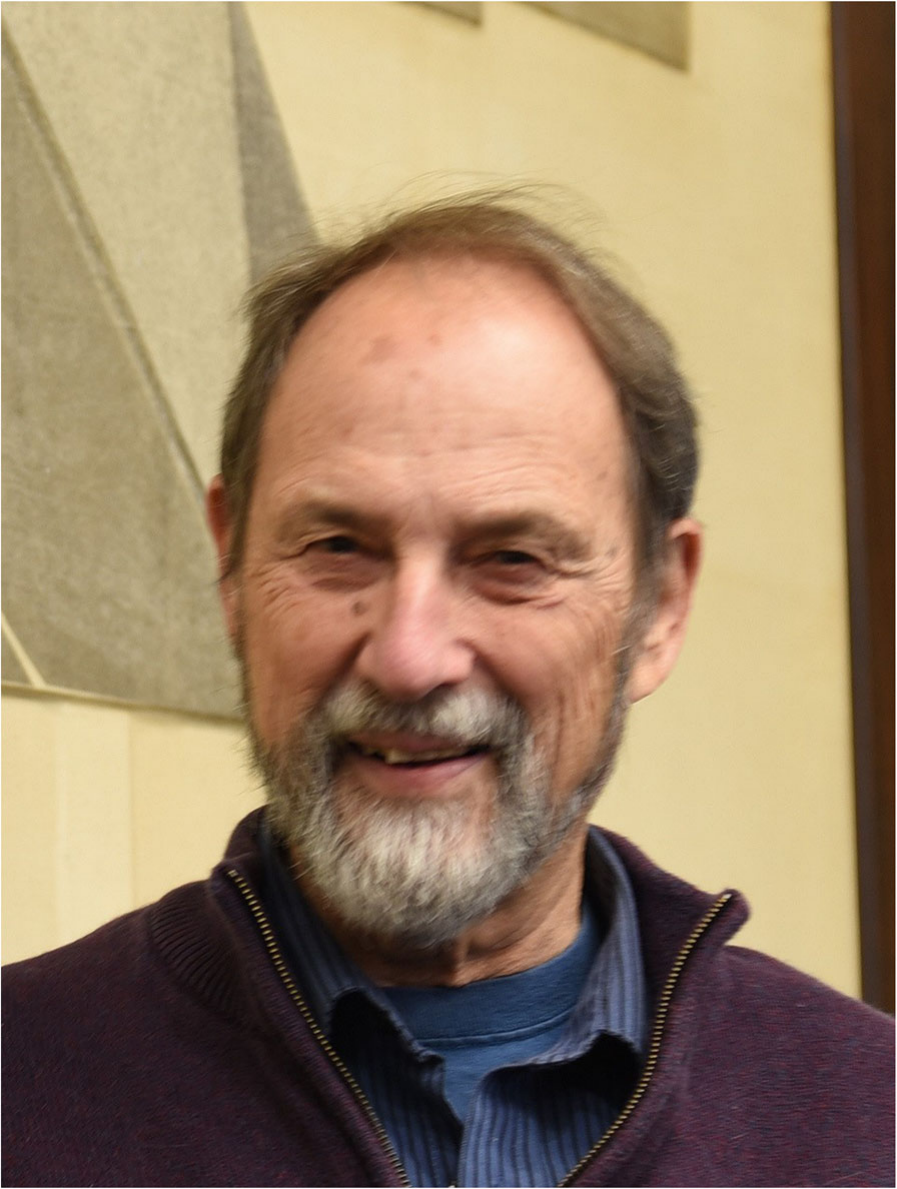
David W. Malloch. Photo: Wen Chen

One of Canada’s most influential and original-thinking mycologists, David “Dave” Malloch, reached 80 on 28 March 2020. He was a student of Harry Thiers and later Roy Cain, and after a brief time at the Biosystematics Research Institute in Ottawa (now Ottawa Research & Development Centre), Dave spent the rest of his career as a professor at the University of Toronto. Known as an inspiring teacher, a keen observer and insightful thinker, Dave trained several PhD students who went on to have distinguished research careers, including Adrian Carter, Leonard Hutchison, Scott Redhead, James Scott, Richard Summerbell, and Wendy Untereiner. After his retirement in 2003, Dave moved to Saint John, New Brunswick, on the east coast of Canada overlooking the mouth of the Bay of Fundy, where he continued to mentor students, participate in bioblitzes, and study fungi as a Research Associate of the New Brunswick Museum. He has continued to keep in contact with his mycological colleagues from his Ottawa days, not least at my retirement party in 2019 along with Kris Pirozynski, Scott Redhead, and the late “Stan” Hughes.

Dave has particularly broad interests in mycology, with expertise in ecology, insect-fungus interactions, and conifer endophytes, and further has made important contributions to the taxonomy of yeasts, agarics, several groups of *Ascomycota*, and asexual fungi. His 1981 book, *Moulds: their isolation, cultivation and identification*, originally published by the University of Toronto Press, is now freely available on his website (http://website.nbm-mnb.ca/mycologywebpages/mycologywebpages.html), along with a unique guide based on HSV colours for use on cell phones, and accessible through the ‘Mushroom Colours’ page of his site.

The IMA congratulates Dave on his 80th birthday and wishes him all the best for the future.

**Keith Seifert**

(stilbella@hotmail.com)

### John W. Sheard – distinguished lichen monographer

(Fig. 28)

**Fig. 28:**
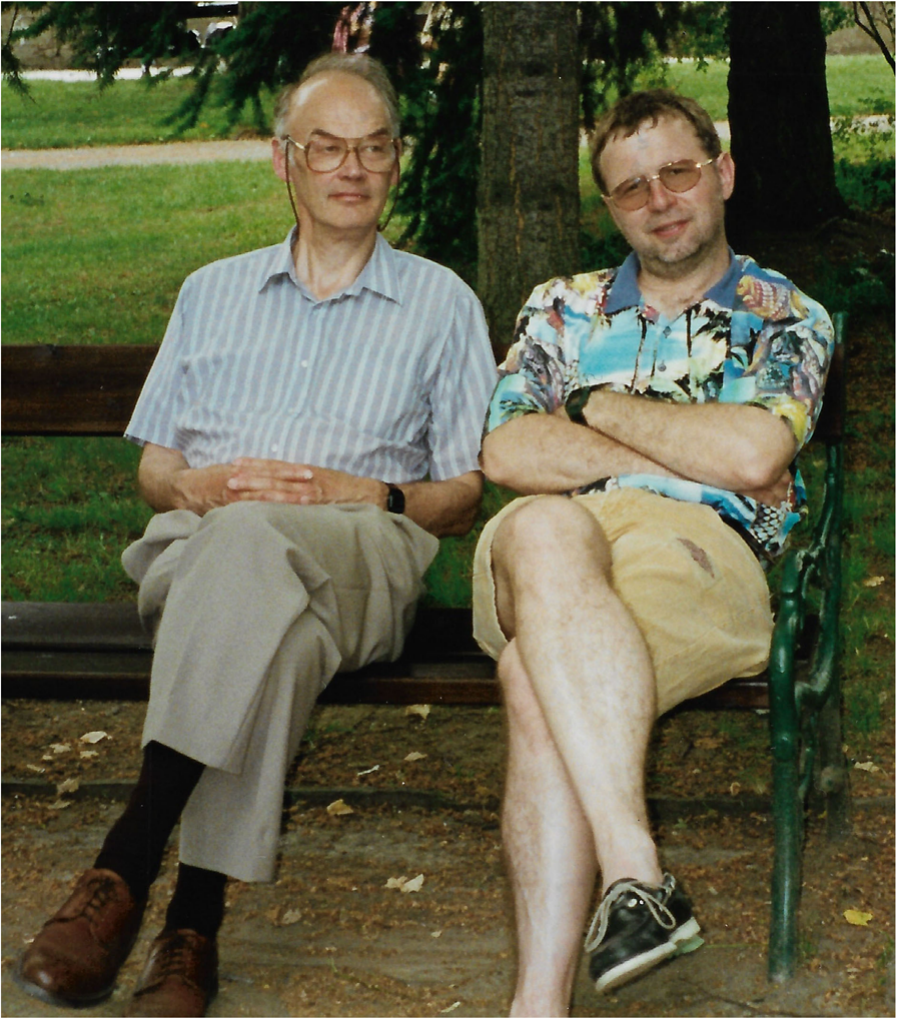
John W. Sheard (*left*) and Helmut Mayrhofer (*right*) in Graz, Austria, on the occasion of his second visit, 1998. Photo: Eleonora Mayrhofer

John, a significant lichenologist and ecologist and long standing professor in Biology at the Department of Biology at the University of Saskatchewan in Saskatoon (Canada), celebrated his 80th birthday this year. John was born and grown up in Yorkshire (UK) and educated at Imperial College London where he receved BSc, MSc and finally a PhD in 1965. His academic career started at Magee University College in Londonderry (Northern Ireland) before leaving the UK for a post-doctoral fellowship at the National Museum of Natural Sciences in Ottawa in 1967. One year later, he was appointed Assistant Professorship in the Department of Biology at the University of Saskatchewan in Saskatoon where he continuously served as Associate Professor (1971–78), Professor (1978–2000), and is now Professor Emeritus. His teaching topics included: General Biology; Survey of Bacteria, Algae and Fungi; Evolutionary Survey of the Plant Kingdom; Biology of Lichens; and Multivariate Methods in Taxonomy and Ecology. He also supervised four PhD candidates in ecology and four MSc ones in ecology and lichenology.

He devoted himself to the huge genus *Rinodina*, on which he obtained his PhD – that included the first 10 km square distribuition maps of UK lichens ever published (Sheard 1967). The genus kept him very busy in preparing many publications on different aspects, mainly in taxonomy but also on ecology and biogeography. These were the results of extremely detailed microscopic studies of thousands of lichen samples extending over more than 40 years, and his 2010 monograph represented a lifetime of research and is a long-lasting milestone in lichen diversity research. His research, however, also includes papers on the taxonomy, ecology and phytogeography of other lichenized genera, such as *Buellia*, *Dimelaena*, and *Thamnolia,* the contraversial *Ramalina siliquosa* group, and further on the vegetation of arctic sites, the distribution of uranium series radionuclides in northern Saskatchewan, and weed communities associated with arable farm management. In 2002, the American Bryological and Lichenological Society (ABLS) gave him the Edward Tuckerman Award for the best paper in the field of lichenology in *The Bryologist,* and in 2017 the Canadian Botanical Association granted John its highest award, the 2017 Lawson Medal on the basis of his landmark monograph on *Rinodina* in North America (Sheard 2010).

Besides his merits as the worldwide expert in *Rinodina*, he is a local expert in vegetation ecology and a well respected university professor contributing to numerous public and community organisations. For example, John has been a long-time member of the Mental Health Advisory Committee to Saskatoon District Health Board. He always enjoys hiking in summer and cross-country sking in winter, and was very active in the Saskatchewan Ski Association in the 1970s and 1980s, serving as its Vice-President.

Despite the many years living and working in Canada John remains an extremely smart and generous English gentlemen, and all his collagues and friends send him our congratulations on this special birthday and wish him continuing enjoyment of life and lichens even in these difficult times.

**Helmut Mayrhofer**

(helmut.mayrhofer@uni-graz.at)

## IN MEMORIAM

### John F. Peberdy (1937–2020)

(Fig. 29)

**Fig. 29:**
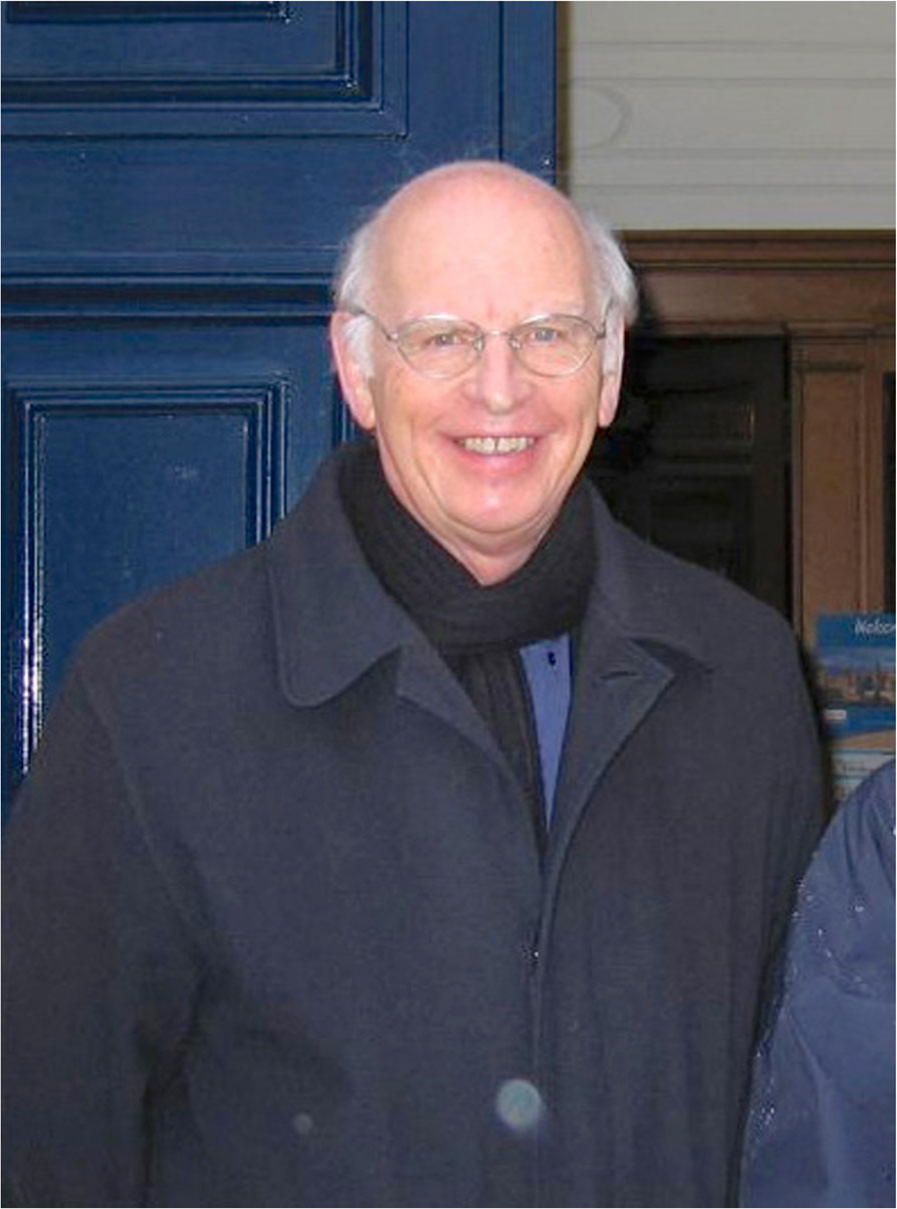
John F. Peberdy

John Peberdy passed away peacefully on 14 May 2020 following a battle with cancer. John studied Botany at the University of Newcastle in the UK where he became fascinated with microbiology and fungi in particular. He studied for a PhD in fungal biochemistry at the University of Nottingham before working briefly in industry at a Water Pollution Research Laboratory. He then returned to academia, first in a lecturing position at Hull before moving back to the University of Nottingham for one in Microbiology in 1966. He spent the rest of his career at Nottingham where he was promoted to a professorship in 1984. John had many wide-ranging impacts during his career. His research field was in fungal biochemistry and genetics, where he was a pioneer in isolated fungal protoplasts and ran one of the first labs to achieve fungal transformation. John mentored over 50 PhD and MSc students, had many European exchange students, and also numerous international collaborators. Throughout his career, he had an immense interest in the practical applications of mycology, particularly in the commercialisation of biotechnology. He established a small company, developed a national UK universities competition, the Biotechnology Young Entrepreneurs Scheme, and in 2000 was appointed a Member of the British Empire (MBE) for services to student entrepreneurship. John was also very active in promoting fungal biology. He was President of the British Mycological Society in 1984 and continued to help organise many BMS and other international meetings. One lasting legacy is that John proposed and launched the European Conferences on Fungal Genetics (ECFG), the first of which was held at Nottingham University in 1992; the biennial ECFG meetings continue to be very popular. John’s passions for microbiology and for training young researchers and entrepreneurs to apply their scientific and technical knowledge continue to be inspirational and he will be greatly missed.

**Paul S. Dyer and Rosie E. Bradshaw**

(paul.dyer@nottingham.ac.uk)

### Nicholas “Nick” Read (1954–2020)

(Fig. 30)

**Fig. 30:**
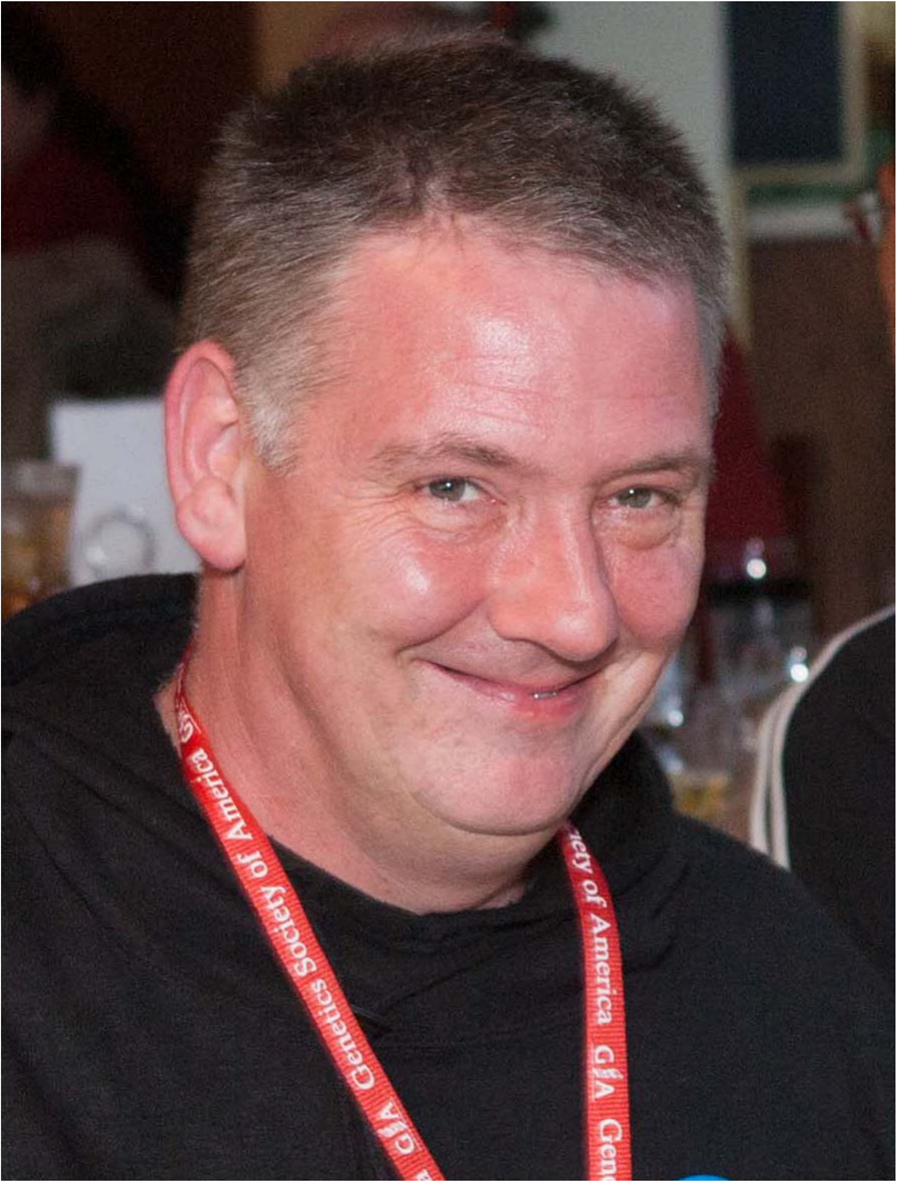
Nicholas Read

It was truly a great loss to international mycology when Nicholas “Nick” Read passed away on 21 March 2020, following a long struggle with illness. Nick was born in London on 7 December 1954, but spent his childhood in South Africa. During his career, he made major contributions both to his research area of cell biology and also the international conference scene. He also had a passion for field mycology and led University field courses for many years.

Nick completed his PhD at the University of Bristol in 1981. He continued to work as a postdoctoral fellow at Bristol before moving to the University of Edinburgh in 1985 where he remained until 2013, being promoted to Reader and Professor in Fungal Cell Biology during this time. Finally, Nick moved to the University of Manchester in 2013 to become Director of the new Manchester Fungal Infection Group where he was pivotal in establishing the international reputation of the group before ill-health, especially amyloidosis, reduced his involvement in recent years.

It was at Bristol that he first published some of his jaw-droppingly good scanning electron micrographs, and over his subsequent research career Nick was well known for his pioneering use of the latest developments in microscopy, for example being one of the first to use confocal microscopy with filamentous fungi, and he liked to push imaging techniques to their limits and beyond. He most recently published some stunning fluorescent images of genetically manipulated fungi to study processes of hyphal fusion. He was very proud of the discovery of conidial anastomosis tubes (CATs), being specialised hyphae formed by the conidia of *Neurospora* and certain filamentous fungi to allow early cell fusion. With support from the British Mycological Society he also co-produced a DVD of video clips of time-lapse hyphal growth and other images that many still use in mycology teaching. He was a great teacher of microscopy, training numerous undergraduates and PhD students, many of whom have gone on to train others and pass on valuable skills of observation. Nick published over 150 publications in his career and a special edition of *Fungal Biology Reviews* will be published in 2021 in his honour.

Nick was a frequent speaker at international conferences and was well known for his good humour and social nature at conferences, allied to a keen alertness to research opportunities that might arise. As well as participating in conferences, Nick helped lead many meetings, most notably IMC9 in Edinburgh in 2010, which was arguably one of the most successful International Mycological Congresses of all time. As well as having superb science content, the Congress involved a very memorable reception including whisky tasting, karaoke, Scottish dancing, live bands, etc. This really reflected Nick’s character as an earnest researcher yet one who really wanted people to enjoy their science. Particularly moving was that one of Nick’s daughters, Hannah an accomplished professional violinist, played live at the event to great applause. Other career highlights were that Nick was President of the British Mycological Society, Chair of the Fungal Genetics Policy Committee, and a Fellow of the Royal Microscopy Society and the Royal Society of Biology. Nick was also a Vice-President and Executive Committee Member of the IMA, which awarded him a Fellow’s Medal from the IMA in 2018 in recognition of his contributions. In one of his later messages, he reported that he was extremely honoured by this award and kept the framed fellowship certificate on the wall next to his hospital bed to inspire him to further contributions to international mycology. Nick is survived by daughters Hannah and Sophie, and wife Shenda. His knowledge and enthusiasm for all things mycological was infectious and Nick is deeply missed.

**Paul S. Dyer and Patrick Hickey**

(paul.dyer@nottingham.ac.uk)

### Anthony “Tony” Peter Joseph Trinci (1936–2020)

(Fig. 31)

**Fig. 31:**
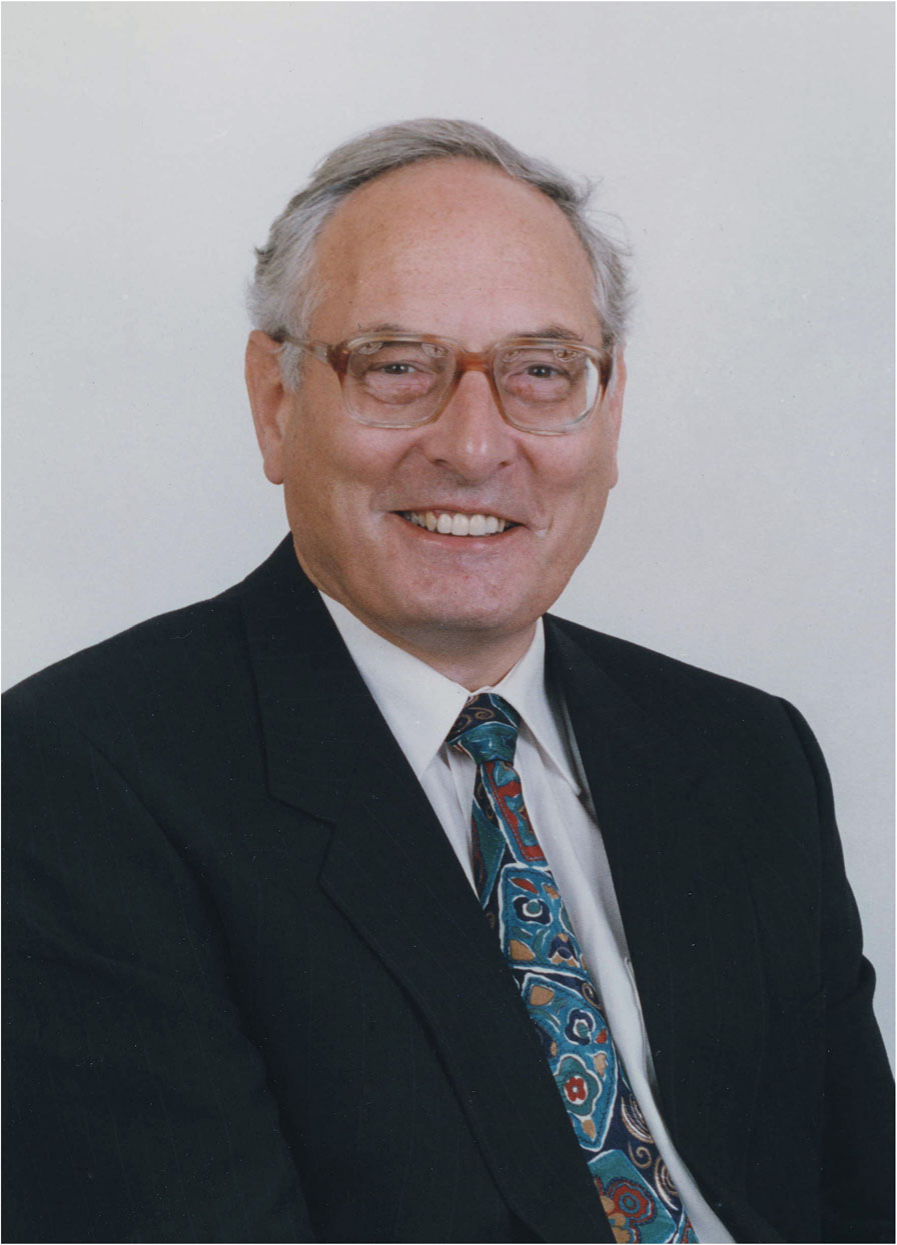
Anthony “Tony” Peter Joseph Trinci

“Tony” Trinci died aged 84 on 7 October 2020. Born in Swindon to parents of Italian descent, he read Botany at Durham University (1959) where he focussed on fungal physiology during an MSc. After a short spell as a school teacher, he returned to Durham and did a PhD with the late Geoff Banbury studying the physiology of tall conidiophores of *Aspergillus giganteus.* In 1964 he moved to a lectureship in the Microbiology Department at Queen Elizabeth College (QEC) London, where he initiated his research on fungal growth kinetics and physiology. In seminal studies, he developed methodologies allowing direct observation of colony growth and organization of the mycelium by hyphal tip growth and branch initiation. He described and defined for the first time a set of parameters for measuring growth of the mycelial colony: Colony Radial Growth Rate, Peripheral Growth Zone, and the Hyphal Growth Unit allowed the definition and modelling of hyphal branching and colony formation using wild-type mycelia and colonial mutants. He complemented this work on surface growth with studies of filamentous fungi in submerged continuous culture describing many aspects of growth physiology.

In 1981 he moved to the Barker Chair of Cryptogamic Botany in Manchester. A great advocate of curiosity-driven research but, intriguingly, his fundamental mycological knowledge became applied to collaborative projects supporting commercial applications of filamentous fungi – notably Quorn mycoprotein developed by Rank Hovis McDougall and Imperial Chemical Industries (ICI) from *Fusarium venetatum*, a human food product that has achieved great commercial success with the rise of vegetarianism. It became apparent that during the fermentation of the filamentous fungus colonial variants arose with short hyphal filaments which could take over the culture and so might impair the product. Tony’s research with Marilyn Wiebe, Geoff Robson (deceased) and Steve Oliver showed how these variants arose and how they could be suppressed.

A second major theme to his research was in a decades-long collaboration with Mike Theodorou, a rumen microbiologist at the Institute of Grassland and Environmental Research, where he studied the intriguing gut anaerobic fungi. Tony’s broad knowledge was applied to understanding the life-cycle and role these fungi played in the rumen. The group elucidated the timing of events of the gut fungal life-cycle, including the identification of a hitherto unrecognised survival stage. They confirmed anaerobic fungi as ubiquitous in the gastrointestinal tract of most large, mammalian herbivores where they digest lignocellulosic plant biomass. Jayne Brookman joined the collaboration in 1994 and molecular approaches allowed identification and classification of these enigmatic fungi. Again, as with Quorn, projects with a commercial partner, in this case Genencor (now part of DuPont), led to practical outcomes. Investigating fungal enzymes from aerobic and anaerobic fungi for use in animal feed they had a marked success with a phytase enzyme, isolated from *Penicillium* species, that was used to release phosphate in animal feeds.

Tony became a leading figure in the changes occurring in biosciences in UK universities over the past 40 years. He recognised the opportunities brought by molecular biology to research and teaching across all biosciences. Soon after his appointment in Manchester he selflessly led change in organization and culture, leading to the first integrated School of Biological Sciences. This pattern, which today provides massive opportunities for research and for students’ learning, is now the norm throughout the UK and in multiple institutions overseas. In mycology specifically, he was again a champion of inter-disciplinarity. Working across unconventional boundaries he facilitated links with medicine and supported David Denning’s development of the Manchester Fungal Infections Group.

He received many prizes and awards; was recognised internationally, and uniquely served as President of the two largest UK professional learned societies in microbiology: the Microbiology Society and Mycological Society. He was awarded the highest award of the Microbiology Society, the Marjory Stephenson Prize, was an Honorary Member of the British Mycological Society, the Microbiology Society and the Mycological Society of America, and a Fellow of the Royal Society for Biology.

He was an inspirational teacher and mentor, an innovative and transformative researcher, and an efficient and visionary administrator. Most of all, however, he was a genuinely nice, considerate and modest person who gave huge amounts of credit and personal support to colleagues and students also giving unselfishly of his time to improve organizations. He will be sadly missed. Outside academia his life was also varied: first and foremost, he was a devoted family man but also involved in socially-focussed politics, school governorships, and charitable organisations. In January 1961 he married Margaret, whom he met at the University of Durham, and she survives him, along with their three children, John, Sarah and Rachel, seven grandchildren, and two great-grandchildren.

A fuller obituary with some more personal appreciations is published on the website of the Anaerobic Fungal Network (https://anaerobicfungi.org/).

**Keith Gull**

(keith.gull@path.ox.ac.uk)

### Balumuri Pandu Ranga Vittal – a distinguished mycologist from India

(Fig. 32)

**Fig. 32:**
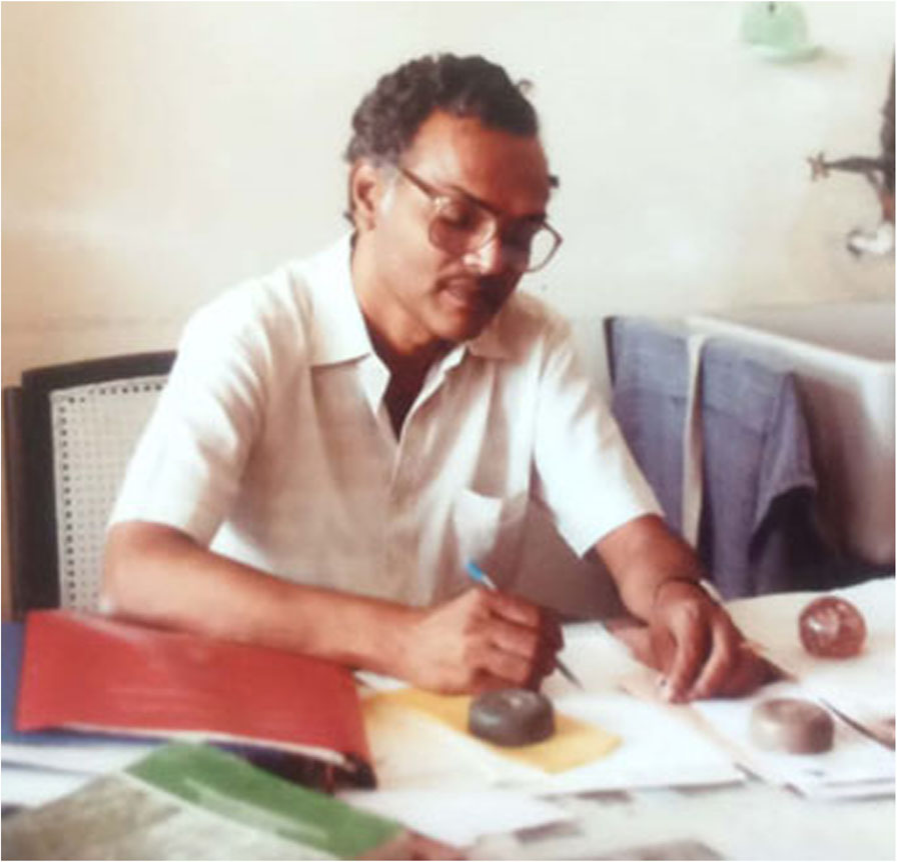
Balumuri Pandu Ranga Vittal in his office in Chennai

India lost one of its most distinguished mycologists, Balumuri Pandu Ranga Vittal on 28 October 2018 to a massive cardiac arrest in Chennai, at the age of 74. He was born on 3 April 1944 in the village of Ravipadu, Cumbum Mandal, in the Prakasam district of Andhra Pradesh. He obtained a Master’s degree in Botany from Andhra University in 1964, and then joined a research project as Research Assistant on the aerobiology of sugarcane pathogens under the supervision of the late Professor T. Sreeramulu. Subsequently, he moved the Centre for Advanced Studies (CAS) in Botany in the University of Madras, and worked under the late C.V. Subramanian – the doyen of Indian mycology. Vittal studied the taxonomy and ecology of leaf litter fungi and obtaining a PhD in 1973. Then, following post-doctoral studies at CAS he was appointed as lecturer there in 1976, becoming a reader in 1984, and professor in 1994 until retirement in 2004; he continued as an emeritus professor there until August 2010. Vittal made visits to the then Commonwealth Mycological Institute at Kew in 1978 and 1981, the then Centraalbureau voor Schimmelcultures in Baarn, The Netherlands, in 1978–1979, and Rothamsted Experimental Station, Harpenden, UK, in 1981 and 1986 (with Philip H. Gregory and John Lacey, respectively). Further visits to the UK followed from November 1986 to August 1987, and 1995.

An admired and inspiring teacher, he was actively engaged in teaching microbial diversity, plant ecology and fungal ecology courses to MSc and MPhil students of the University of Madras. He worked on the diversity of fungi colonizing decomposing leaf litter in terrestrial ecosystems and marine fungi colonizing woody litter in mangroves. Apart from studies on the taxonomy and ecology of litter fungi, Vittal initiated and developed an active school of aerobiology in CAS. He guided 26 MPhil and 11 PhD scholars for their degree, and has over 80 publications. He managed projects sponsored by various national funding agencies and successfully handled 10 major research projects in mycology and aerobiology.

Along with co-workers, he introduced several new genera and species and published biodiversity and ecological observations of leaf litter fungi from different plant hosts in forests of the Eastern Ghats, initially with Subramanian and later his students T. Saravanan, S. Shanthi, and B. Sampath Kumar. Vittal also initiated detailed work on diversity and ecology of marine fungi in mangroves with several colleagues. In parallel, he continued aerobiological and biodeteriogenic studies, and surveyed and enumerated airborne fungi and pollen grains from diverse urban and rural environments including work-places, libraries, museums, and poultry farms – also collaborating with allergy specialists.

Vittal served as President of the Indian Aerobiological Society (1994–97) and Treasurer, Secretary and then President of the Mycological Society, of India (1994–2004). He was a founder member of the Mycological Society of India and member of the International and Indian Aerobiological Societies. He was honoured by Fellowships of the Indian Aerobiological Society and Madras Science Foundation, and received Life-time Achievement Awards from the Indian Aerobiological and Mycological Societies of India. The fungal genera *Vittalia* and *Vittaliana* were also coined in this honour.

In addition to being an excellent teacher and dedicated researcher, Vittal was a wonderful human being, a man of few words with great humility and simplicity, and affectionate, soft spoken and often witty in conversation. With his passing, India lost an accomplished mycologist. He is survived by his wife, daughters, sons-in-law, and grandchildren.

**V. Venkateswara Sarma**

(sarmavv@yahoo.com)

## BOOK NEWS

### 21st century guidebook to fungi

#### By David Moore, Geoffrey D. Robson and Anthony P. J. Trinci. 2nd edn, 2020. Cambridge: Cambridge University Press. Pp. x + 600, illustr. (many col.). ISBN 978-1-108-74568-0. Price: £ 49.99 (Fig. 33)

This textbook was first published in 2011 (see *IMA Fungus* **2**: (62), 2011), where it met a need for advanced mycological courses, especially those with a fungal biology, experimental or applied mycology focus rather than a primarily systematic one. The titles of 17 of the 19 chapters are as in the first edition, the former one on “Molecular biotechnology” has disappeared, and there are new ones on “Fungal genetics: from gene segregation to gene editing” and “Killing fungi: antifungals and fungicides”. Do not, however, be misled as there has been a thorough section-by-section updating and reworking to reflect the enormous strides that have occurred in our understanding of fungal biology during the last decade. The whole also benefits from the inclusion of full colour illustrations in the body of the work, rather than the somewhat inadequate half-tones in the text with coloured versions as a discrete tipped-in signature of the first edition. In addition of colour is also now used in many of the line illustrations and in section headings, and the references at the end of each chapter are printed on grey; these changes make the whole more attractive and will surely increase the appeal to students. I found the new chapter on “Fungal genetics” especially well-written and presented, with succinct explanations and developments placed in an historic context, and as up-to-date as one could hope for with references including ones from 2019; this is a primer that can be commended to any mycologist wanting a clear exposition of this rapidly advancing field. The two Appendices of the first edition are retained, an updated outline classification as in the previous edition; an outline classification down to order for all fungal-like organisms (including oomycetes and slime-moulds), and a superb overview of mycelial and hyphal differentiation including tissue types, hyphal systems, methods of conidiogenesis, etc., incorporating the meaning of the numerous specialized terms used, and now benefitting from the use of colour. An extraordinary number of references to original literature sources are again a feature throughout the book, and this adds immensely to its’ value as an information source. There is, however, no indication that a companion CD, which was included in the back of the first edition and incorporated hyperlinks, is to be made available this time; something partially mitigated by the inclusion of DOI and URL internet strings at the end of most references, and understandable in an era where CD-drives are becoming rarer on lap-top and other personal computers .

**Fig. 33:**
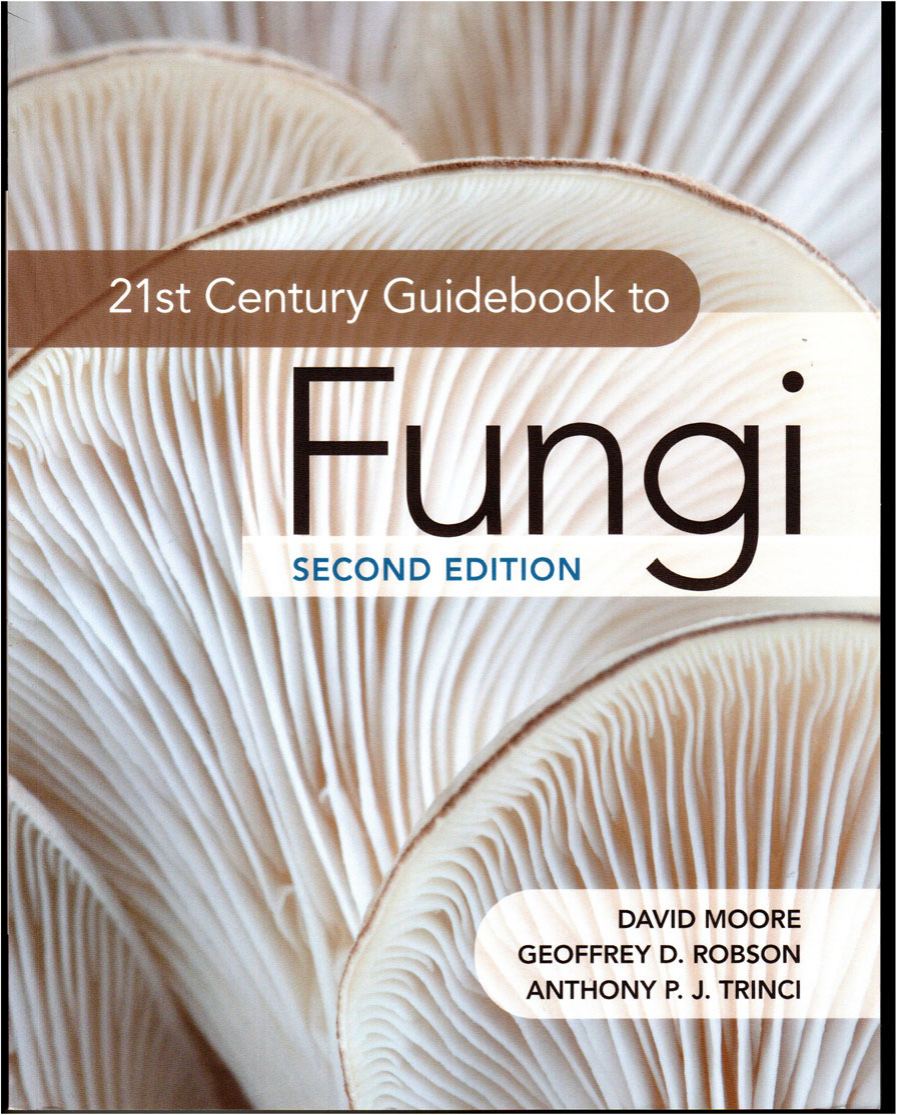
*21st Century Guidebook to Fungi* (2020)

The first edition received wide acclaim, and several glowing comments are repeated in the Preface, and this is certain to be the case with this new one. I was privileged to be sent a copy of the proofs, on which I commented that “. .. this provides a masterly, enticing, and fascinating window into the mysteries and marvels of the biology and physiology of fungi; their life-styles, how they develop and grow, their chemical armoury, beneficial and harmful interactions with diverse other organisms, ecological roles, and importance to human well-being”, as recorded on the back cover. This is a real *tour-de-force*, by three exceptionally talented and experienced lecturers in mycology, and can be unhesitatingly recommended, though I fear the price, which is reasonable for such a substantial and well-prepared work, may nevertheless place it beyond the pockets of all but the keenest students.

The book is dedicated to “Geoff” Robson who passed away suddenly in May 2018 while this text was still being worked on, and even more sadly “Tony” Trinci died shortly after its’ publication (*see earlier in this edition of* MycoNews).

### Lichen secondary metabolites: bioactive properties and pharmacological potential

#### Edited by Branislav Rancović. 2019. 2nd edn. Cham: Springer Nature Switzerland. Pp. v + 260, illustr. (some col.). ISBN 978-3-030-16813-1 (hdbk), 978-3-030-16814-8 (ebk). Price: 135.19€ or £ 109.99 (hbk), 106.99€ or £ 87.50 (ebk) (Fig. 34)

The first edition of this work appeared as recently as 2015 (see *IMA Fungus* **6**: (30), 2015) so a second was something of a surprise. It is, however, very much an update and has grown by 58 pages. The chapters are the same, with the same authors, plus one welcome additional one. Seven of the eight original chapters are updated with references to more recent work, especially those on traditional medicinal uses and antigenotoxic effects that may have potential as cancer treatments. I found the new chapter especially fascinating. It concerns antineurogenic and antidiabetic activities, and lucidly explains the diseases and how they may be targeted. The book is well-produced but I was sad to see that there was still no index, something drawn attention to in my comments on the first edition; surely it would have added value to the work if they had been one covering the species and compounds that are cited. The overall impression of the book is that while there is enormous potential for the exploitation of natural products from lichenized fungi, it has to be recognized that major obstacles remain to be overcome in order to realise those benefits.

**Fig. 34:**
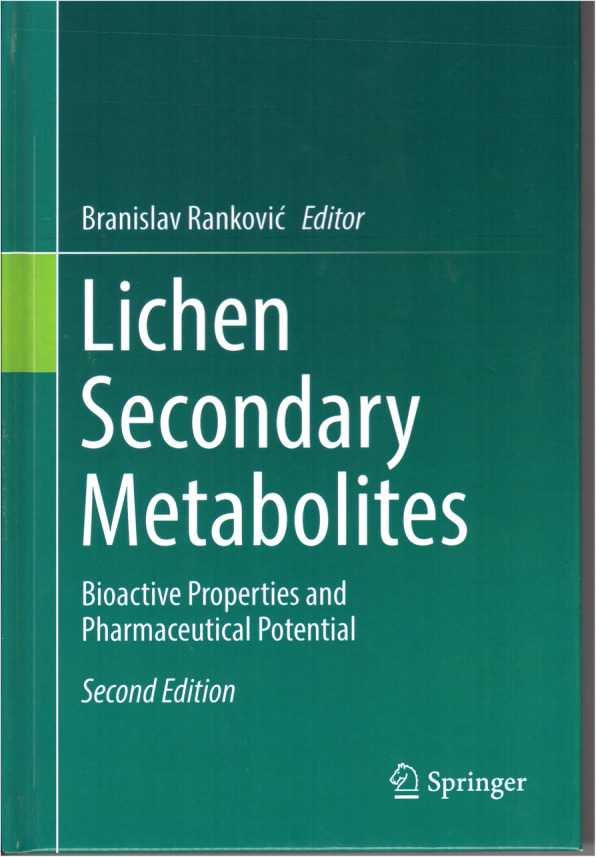
*Lichen Secondary Metabolites* (2nd edn, 2019)

### Fungi of Antarctica: diversity, ecology and biotechnological applications

#### Edited by Luiz Henrique Rosa. 2019. Cham: Springer Nature Switzerland. Pp. xv + 345, illustr. (colour). ISBN 978-3-030-18366-0 (hbk), 978-3-303-18369-1 (pbk), 987-3-030-18367-7 (ebk). Price: £ 109.99 (hbk), £ 89.99 (pbk), £ 87.50 (ebk) (Fig. 35)

A thorough synthesis of where we are with knowledge of the fungal organisms of Antarctica is long overdue, so I was so pleased to see this title. Sadly I was disappointed to find the title somewhat misleading. This work contains 15 multi-authored contributions, involving 57 authors of which 50 are from South America; ten have the editor as a co-author. Having said that, there is, nevertheless some very useful information compiled here, focussed on fungi cultured and (or) detected by molecular methods, from different organisms or other substrata. There is a series of chapters concerned with particular habitats: soils, lakes, rocks, snow and ice, permafrost, plants and lichens (considered only as a habitat), and invertebrates. Almost all of these have checklists of the fungi reported, arranged by host or locality, and with the dispersed literature sources cited, and this is the great value of the book, though I have to wonder of the value of entries such as “*Penicillium* sp. 6”! I was, however, especially disappointed to find the very considerable literature on lichenicolous fungi overlooked, and also that on non-lichenized macromycetes (e.g. Pegler et al. 1980). It would also have been helpful for researchers to have the book by Onofri et al. (2007) cited and drawn to their attention; that has detailed descriptions, drawings, and locality information on the microfungi known from cultures and specimens which would supplement and extend what is reported here. I found the chapter of studies using cultivation-independent approaches of particular interest; they include “microscopy” (but not traditional light microscopy) and molecular approaches, again with a tabular compilation of studies including details of the samples, localities, methodologies, and references, in this case extending over five pages. There are also contributions concerned with the fungi isolated as sources of bioactive compounds, enzymes, pigments, and the use of psychrophilic yeasts in the biocontrol of the post-harvest low temperature storage of fruit. In parallel with the habitat-orientated chapters several of these have useful tabulations of what activities or products have been reported by which fungi, along with the original references. A final chapter provides an introduction to genomic approaches, which are evidently very much in their infancy as regards Antarctic fungi, and it will be interesting to see how these develop and add to our understanding of the diversity of fungi in the continent.

**Fig. 35:**
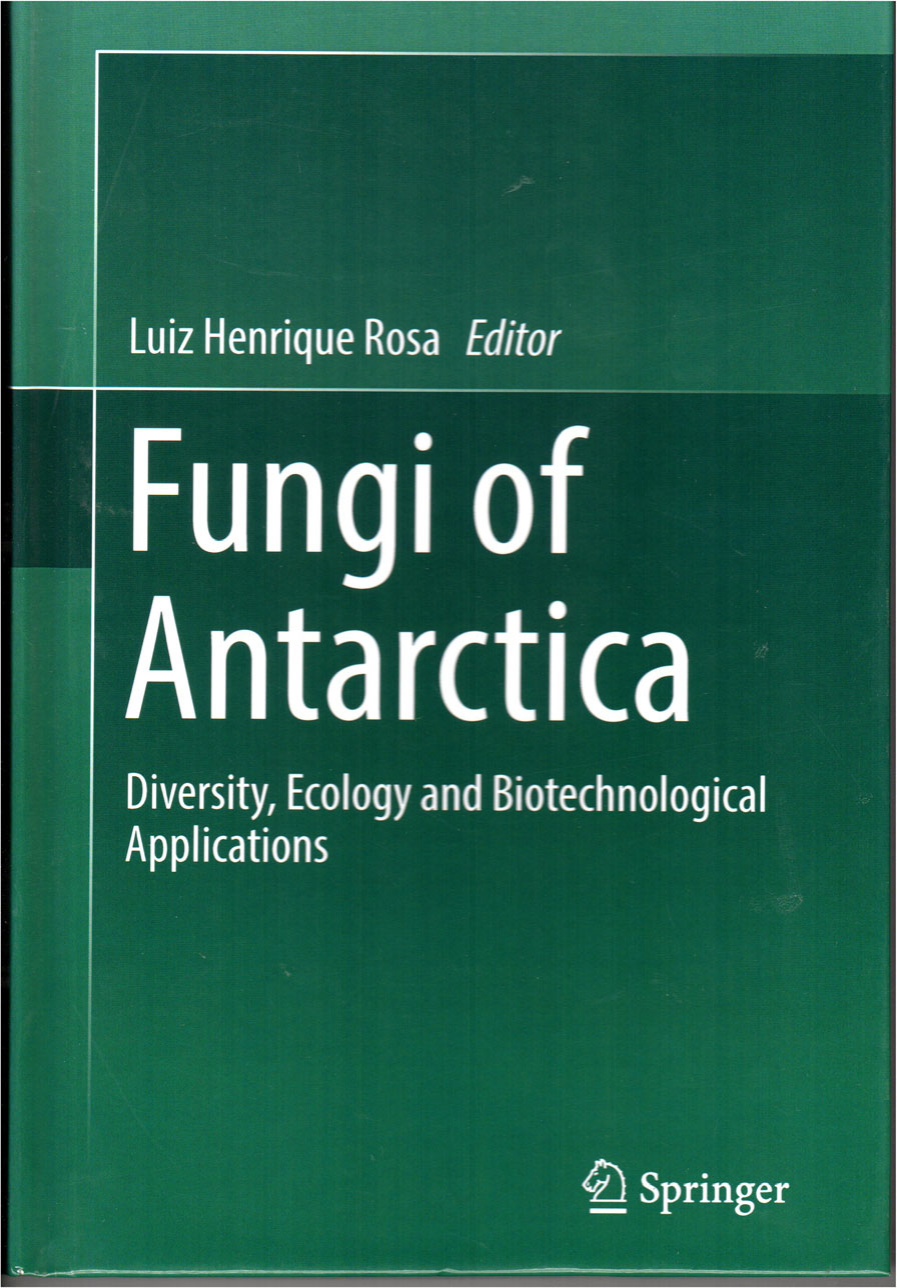
*Fungi of Antarctica* (2019)

The book is well-produced, and it was particularly pleasing to see the high quality of the numerous habitat photographs in particular distributed through the volume as that has been an issue with some other titles from this publisher in the past. It was also good to see an index, even though it only includes a small selection of the scientific names in the text. The main value of the book to me, however, is the tabulations and the contributors are to be thanked for undertaking those time-consuming compilations. The niche in the literature for a comprehensive study of the non-lichenized fungi of Antarctica sadly remains to be filled.

### Food and indoor fungi

#### By Robert A. Samson, Jos Houbraken, Ulf Thrane, Jens C. Frisvad, and Birgitte Andersen. 2nd edn, 2019. [Westerdijk Laboratory Manual series no. 1 (2nd edn).] Utrecht: Westerdijk Fungal Biodiversity Institute. Pp. 481, illustr. (most in colour). ISBN 978–94–91751-18-9. Price: 85.00 € (Fig. 36)

The first edition of this title appeared in 2010 (see *IMA Fungus* **2**: (31)–(32), 2011) and, prepared by several of the most experienced researchers in the field, has become invaluable for all involved in the detection and identification of food and indoor fungi. The same team produced this new edition, which is now just almost 100 pages longer, but has undergone major rearrangements. The initial chapter is now an overview of their taxonomy and nomenclature, including an explanation of the effects of moving to the “IF=IN” system, and two tables showing the older and now current names of the filamentous fungi treated and yeasts commonly encountered, respectively. Then follow overviews of the taxa in *Mucoromycota*, *Ascomycota*, and “Asexual fungi” included along with comprehensive keys, dichotomous and (or) synoptic to the species. It is most valuable to have these all brought together in one section, and those on *Aspergillus* and *Penicillium* are especially detailed also with superb colour photographs of cultures grown under standardized conditions. The second chapter forms the bulk of the book at 320 pages taking each treated species in turn; in the new edition all are arranged alphabetically which is a real bonus for quickly locating a particular species. As before, each species has a double-page spread: each left-hand page with the accepted name and any key synonyms, key cultural and descriptive data, notes on separations from similar species, and information on molecular markers,” typical” cultures, chemical products, habitat and distribution, applications, and key references; the opposing right-hand page has truly superb illustrations, in colour or DIC, of both cultures on different media and microscopic details. The authors estimate that there are only around 175 commonly encountered food and indoor fungi, and of those some 160 are treated in depth.

**Fig. 36:**
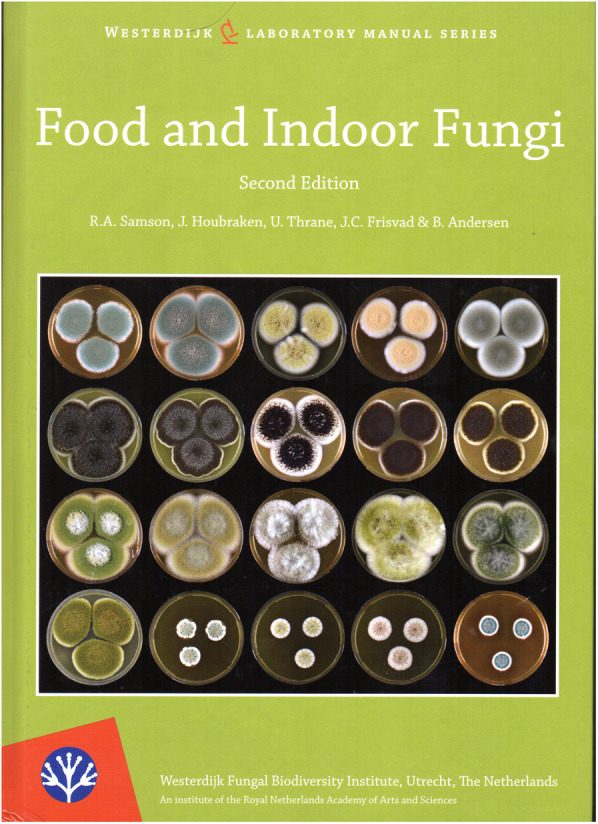
*Food and Indoor Fungi* (2nd edn, 2019)

The final series of relatively short chapters introduce methods for identification (microscopic, molecular and chemical) and the detection and isolation of, separately, indoor fungi and food spoilage fungi. These are all practically focussed, and based on the authors’ decades of experience assessing and addressing actual fungal contamination issues, and with key processes illustrated in colour step-by-step and hands-on. A series of short appendices then summarize the fungi most frequently causing issues with particular food products and in buildings, compounds (including mycotoxins) produced by particular species, provide a glossary of terms, and media recipes. These are followed by 13 pages of references and an index to the species.

This is an unparalleled resource for all concerned with food spoilage and indoor mould issues, and the authors cannot be praised more highly for sharing their unrivalled expertise to produce this new edition. This book must be considered the indispensable *vade-macum* for all practitioners of these areas of applied mycology – although those concerned with food spoilage would benefit from also having Pitt & Hocking (2009) to hand for supplemental information on particular food products and also the species found on them.

### Fungarium

#### Curated by Katie Scott and Ester Gaya. 2019. London: Big Picture Press. Pp. ix + 64, illustr. (colour). ISBN 978-1-78741-512-6. Price: £ 20.00, US$ 47.00 (Fig. 37)

When I first received my copy of *Fungarium* I was not sure that I would enjoy reading it. I thought it looked a bit simplistic and lacked the colour plates usually associated with popular fungal books. Having read it from cover to cover, I realized I was entirely wrong and found the different galleries provided a clear and interesting insight into the fungal kingdom. The Preface highlights their different life-styles and diversity of form and, importantly, points out their essential activities in the service of mankind. We could not survive without them!

**Fig. 37:**
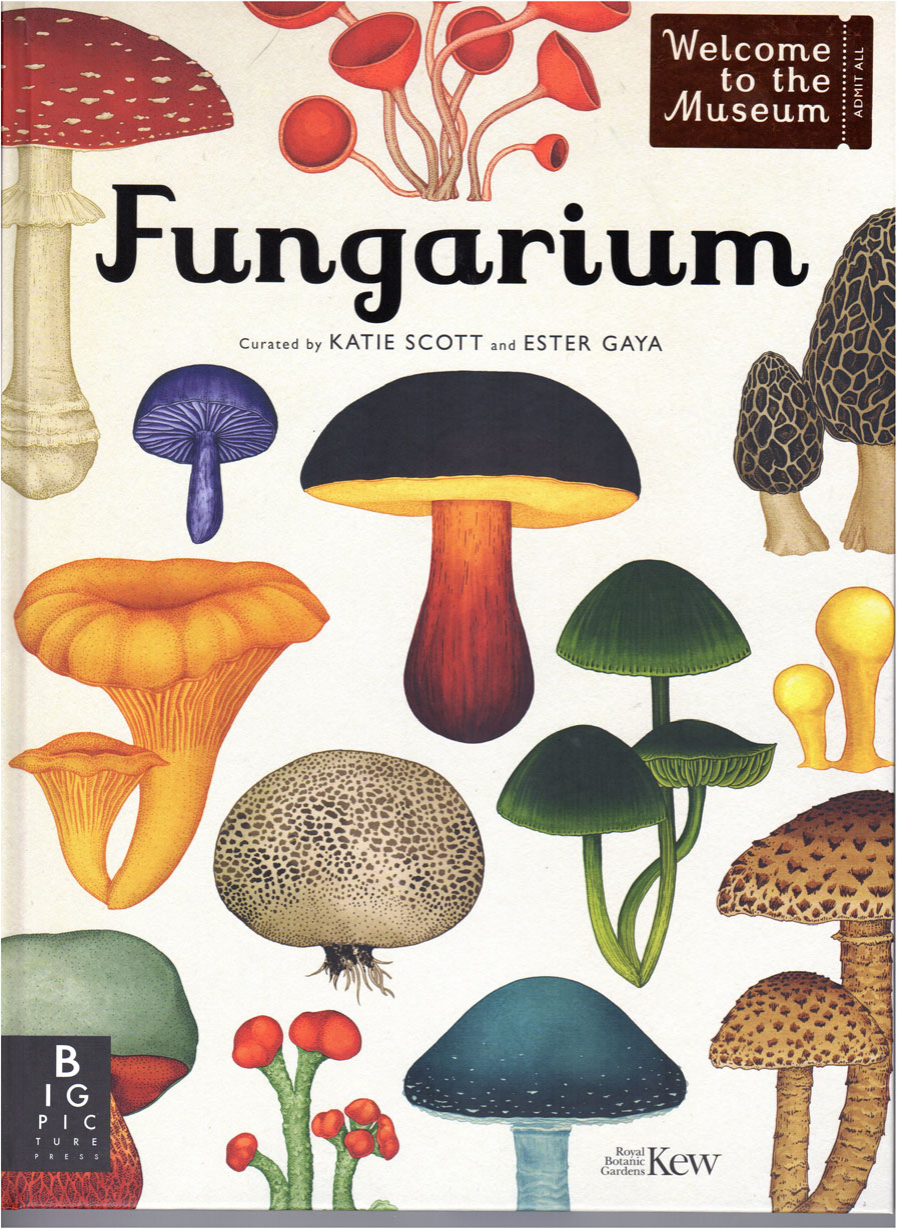
*Fungarium* (2019)

The Museum is then open to a series of galleries and the very varied attributes and activities of fungi. As with any good story there is a beginning and the tree of life explains in clear terms the evolutionary biology of fungi and how DNA studies have transformed our understanding. This is immediately followed by ‘what is a fungus’; not an easy exercise but suitably outlined here. The galleries then take us through the processes of sexual and asexual reproduction, followed by the range of spores produced and useful information on their growth characteristics. All very well illustrated to highlight their special features.

As you might expect in a publication from RBG Kew there is a strong commitment to ecosystems and fungal diversity. These include Mountains, Temperate Forests, and Tropical Forests linked together with familiar major fungal types such as Cup Fungi, Mushrooms and Toadstools, Bracket Fungi, Gasteromycetes and perhaps the less recognized Foliicolous Fungi which are of considerable interest and importance in tropical plants. I am, however, not sure why Tropical Forests are placed in the gallery with Fungi and Humans and not with Temperate Forests under ecosystems which would seem more appropriate.

Having set the scene, Gallery 3 Fungal Interactions investigates fungal relationships in the natural world. These include mycorrhizas and their networking and the massive importance of different mycorrhizal associations in all plant communities is stressed. In relation to our forests the phrase ‘No fungus no forest: no forest no future’ is more relevant than ever. Then there are the lichens with their fungal and photosynthetic partners,which are either algae or cyanobacteria and sometimes both. Lichens are extremely long living, can act as indicators of pollution and some are widely distributed, often occurring in nutrient poor environments. Fungi also form interesting interactions with many insects and the galleries Entomogenous Fungi and Ants and Termites introduce their fascinating associations and activities. There is a brief note and illustration of the Chinese caterpillar fungus, *Ophiocordyceps sinensis*, which is the most expensive fungus known and the most famous of the ones used in traditional Chinese medicines.

The final section of the book provides a brief insight into early mycologists and their contributions, fungi causing diseases of plants, and the danger of poisonous fungi which are fortunately in the minority. Many fungi are also edible and are widely cultivated as a valuable food resource. A number of wild mushrooms are expensive gourmet delights. However, a section on fungal pathogens of man might have been of interest since they are of major significance in disease and mortality.

The gallery on wonder drugs from fungi stresses their major medical contributions as antibiotics, immunosuppressants, and statins. There is no question that these fungal products are essential to life today, but it would have made a stronger statement to bring together all major commercial contributions made by fungi. References to many are scattered throughout the galleries which dilutes their impact. Some examples are ergotamine, widely used to treat migraine, lichen extracts used in perfumes, toothpaste and some as medicines. The bracket fungus *Ganoderma* “*lucidum*” or Lingzhi, an important traditional medicine in China and other Asian countries, is taken to stimulate the immune system in the treatment of a range of conditions including some cancers. There are also fungal enzymes, alcoholic fermentations, baking, gasohol, and the warfarin story – all major contributors. As the preface stresses, there are perhaps as many 3.8 million species of fungi on Earth and without them our environment would not be as we know it today. We should regard the fungi as a major living resource of vital importance to the world we live in.

It is easy to be critical, but the sheer number of fungal species and their activities inevitably means some selection is necessary but without doubt *Fungarium* will open the eyes of many readers to the fascination and significance of fungi. It will make an excellent gift and a perfect addition to join the literature in libraries and as a coffee table talking-point.

**Anthony A. J. Whalley**

(A.J.Whalley@ljmu.ac.uk)

### Entangled life: how fungi make our worlds, change our minds, and shape our futures

#### By Merlin Sheldrake. 2020. London: The Bodley Head. Pp. 358, illustr. (16 pp. col.). ISBN 978-1-847-92519-0. Price: £ 20.00 (Fig. 38)

I received excited and effusive messages about this book from several colleagues in Central Europe and North America before a copy had even landed on my desk. The author undertook his doctoral research on mycorrhizal communities on Barro Colorado Island (Panama) and became fascinated by the diversity and interactions of the diverse fungi he encountered. I can really appreciate that fascination as my memories of those same forests from a visit in 1995 remain imprinted in my visual memory – including arrays of *Cookeina* and *Xylaria* species and watching leaf-cutter ants hard at work. Merlin has put a truly extraordinary amount of research into this, and facts are all carefully referenced with detailed endnotes collected at the back of the book with supporting details and references; together the notes and references, all in 9 pt type, occupy 83 pages, almost 25% of the book! But he did not stop there, he also looked out and interviewed a wide range of really key players and original thinkers in mycology or organismal interactions; 63 of them are listed in the Acknowledgements, and include many familiar names, such as Martin Bitartondo, Lynn Boddy, Keith Clay, Mark Fricker, Trevor Goward, David Hibbett, Peter McCoy, David Read, Jan Sapp, Marc-André Selosse, Toby Spribille, and Paul Stamets. Some of these interviews provide intriguing glimpses of the personal attitudes and broader thinking of some researchers that have never appeared in print. He skilfully interweaves what he has learnt from his extensive researches with his personal experiences. These range from childhood experiments on compost with his father, tropical fieldwork, and a fascination with mycoheterotrophic plants, to participation in experiments with hallucinogenic drugs in a clinical drug testing unit.

**Fig. 38:**
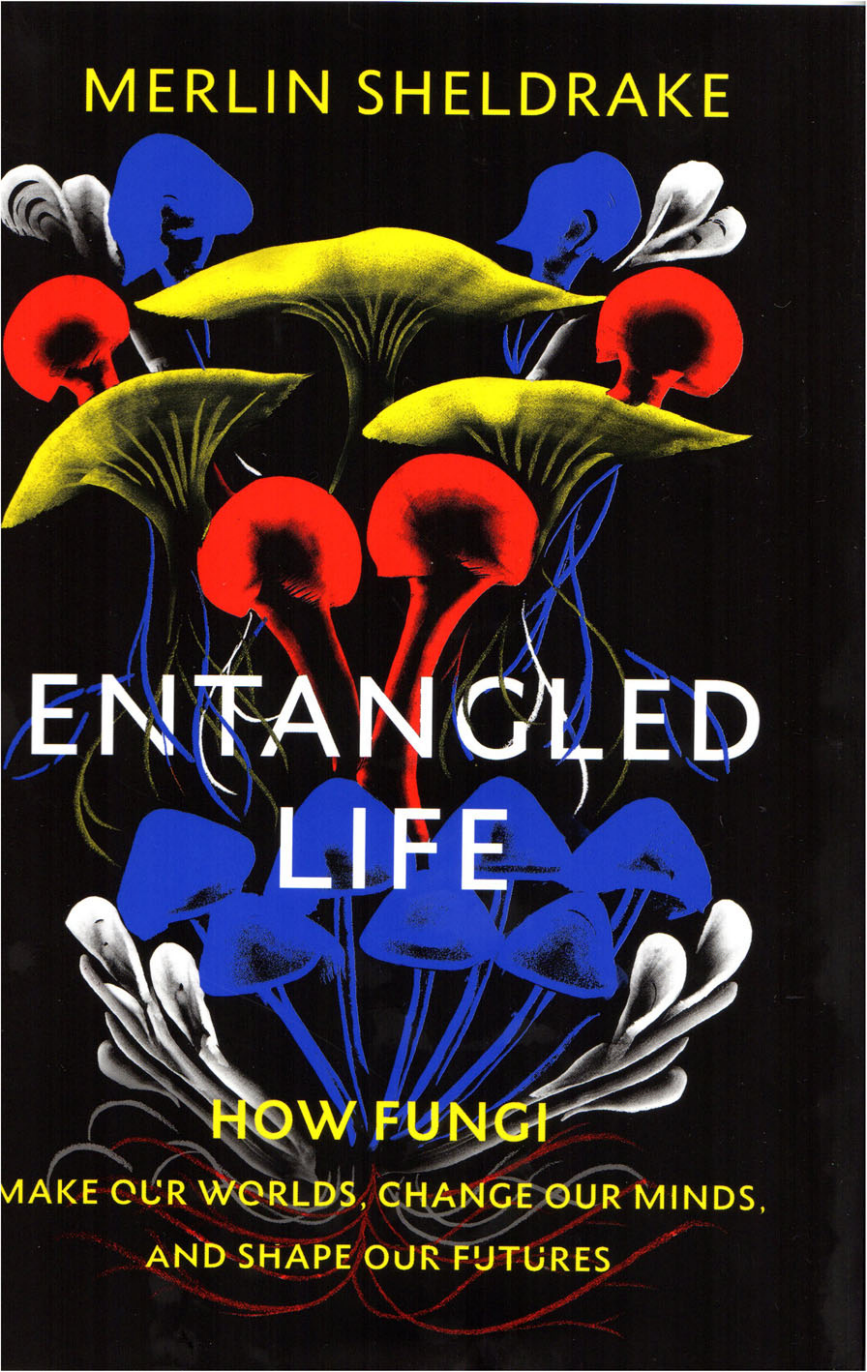
*Entangled Life* (2020)

His exposé is organized into intriguing and imagination-catching titles: What is it like to be a Fungus, A Lure Living Labyrinths, The Intimacy of Strangers, Mycelial Minds, Before Roots, Wood Wide Webs, Radical Mycology, Making Sense of Fungi, and This Compost. There are neat little sketches throughout, which he prepared using *Coprinus* ink, some more accurate than others, and a super central tipped-in 16 pages of coloured illustrations; these include original some from his own mycorrhizal work as well as some taken from the literature which make particularly spectacular points, such as the foraging behaviour of mycelia.

As commented in quotations on the back cover, this is “A dazzling, vibrant, vision-changing book” and “One of those rare books that can truly change the way you see the world around you”. There is much here that I am sure will benefit any mycologist. If it can be circulated and read widely, however, it has the potential to positively contribute to a much wider understanding of the key role fungi have in nature and in our lives amongst not only ecologists and other biologists, but funding bodies and the public at large. It is a book that I, and I am sure many other mycologists, would love to have written. Merlin deserves all our congratulations on realising such a tour-de-force, and I encourage all readers of *IMA Fungus* not only to secure a personal copy, but ensure it gets into their public as well as institution’s libraries.

### Endolichenic fungi: present and future trends

#### By Manish Tripathi and Yogesh Joshi. 2019. Singapore: Springer Nature Singapore. Pp. xv + 180, illustr. (some col.). ISBN 978-981-13-7267-4 (hdbk), 978-13-7270-4 (pbk), 978-981-13-7268-1 (ebk). Price: £ 129.99 (hbk), £ 99.99 (pbk), or £ 103.50 (ebk) (Fig. 39)

This the first book of which I am aware devoted entirely to endolichenic fungi, fungi that are presumed to live asymptomatically inside lichen thalli which are recovered by culturing or indicated to be present by molecular techniques. These fungi are generally related to saprobic and plant pathogenic fungi rather than to either lichen-forming or obligately lichenicolous fungi. The co-authors, both from Kumaum University in Uttarakhand, India, start with chapters discussing first the nature of lichens and then summarizing research on endolichenic fungi to date; both are extensively referenced and provide an excellent starting point for anyone wishing to start investigating endolichenic fungi, with references running into 2017. A short practical chapter then describes the collection, surface sterilization, culture, and isolation of natural products; this would have benefited from expansion to include more hands-on instructions and the inclusion of references to some key general mycological texts on culture techniques, such as Crous et al. (2019; see *IMA Fungus* **10**(23): 14–15, 2019). The same concerns are pertinent to the next chapter on identification of the fungi which focusses on molecular approaches; for references to major works of value in identifying the fungi likely to be isolated it is necessary to refer to the penultimate chapter. I was also surprised to see the phrase “conidial fungi (*Deuteromycotina*)” (p. 62) used in a work published last year and find that rather worrying.

**Fig. 39:**
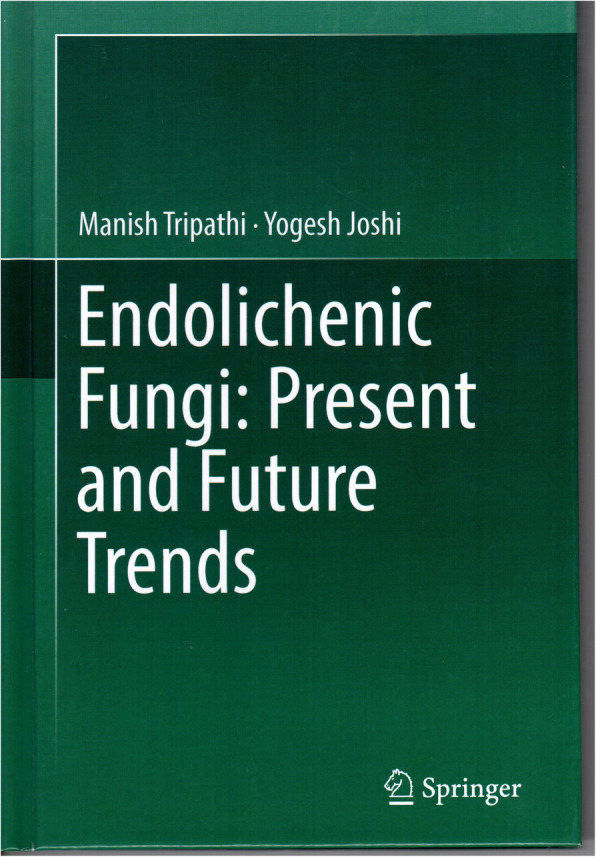
*Endolichenic Fungi* (2019)

The chapter I found of most value in the book was a tabular compilation of the fungi isolated from particular hosts, the compounds identified, and any bioactive properties recorded – all with references to the source literature. Also provided are the structural formulae of an amazing 351 compounds recovered. The authors also describe in detail their own studies in Uttarakhand, during which they isolated 42 species of fungi from 22 macrolichens, tested some for antibacterial activity, and analyzed the major constituents of several species. The penultimate chapter aims to provide “taxonomic descriptions of endolichenic fungi”. It provides a somewhat idiosyncratic selection of works of value in identification, several of which are obsolete and while other key more recent ones are missing, before proceeding to textual descriptions of 70 genera. These accounts unnecessarily include places of publication of the generic names, several of which are sadly incorrect, but does not take the opportunity to reference works of value for species-level identification. All the genera apart from one comprise species including common moulds, saprobes, and plant pathogens; only one is an obligate lichenicolous fungus.

The final chapter looks to future perspectives and challenges, many of which are shared with similar fungi isolated as endophytes from plant material. Indeed, there is still uncertainty as to what extent the isolates represent taxa unknown from other habitats, and whether the cultures resulting from such studies arise from fungi actually living inside the lichen thallus or from spores trapped in the tissues not removed by surface sterilization. Notwithstanding these fundamental issues, this book provides a valuable synthesis of the current state of research on endolichenic fungi and the authors deserve to be congratulated on that achievement.

### Fungi in polar regions

#### Edited by Masaharu Tauji and Tamotsu Hoshino. 2019. CRC Press, Boca Raton, FL. Pp. v + 138, illustr. (col. pl. 5). ISBN 978-1-138-08970-9 (hbk), 978-1-315-10908-4 (ebk). Price: US$ 189.95 or £ 121.00 (hdbk), US$ 52.16 or £ 37.79 (ebk) (Fig. 40)

This little book was conceived primarily to mark anniversaries of the establishment of the Japanese Antarctic Research Expedition (JARE) in 1957 and of an arctic base station in Svalbard (Spitsbergen) in 1991. It presents results obtained primarily from the isolation of cultures and next generation sequencing (NGS), and also work on ecology and biotechnological applications. It opens with an annotated checklist of the non-lichenized fungi recorded around the Syowa station in Antarctica; most are from soil and lake sediments, and with identifications checked by DNA sequence data when available. Other chapters compare the fungi isolated from decaying vascular plant and bryophyte remains at a site in the arctic and one from the Antarctic, considering succession and also the lengths of hyphae in *Salix arctica* in the arctic site; species associated with snow and their ecology; *Rhytisma polare* on *Salix polaris*, its growth rate and its role in carbon cycling, including a list of plant pathogenic fungi on Svalbard; a review of metabarcoding studies in polar regions with discussion of the methodology and issues over interpretation; *Pythium polare* infecting moss tissues, including information on growth rate and ascospore discharge in Spitsbergen, and none on other oomycetes in polar regions; biotechnological potentials of arctic fungi, with an extensive list of enzyme-producers and observations of antioxidant, anti-freeze and polyunsaturated fatty acid production potentials; low temperature waste-water treatment to remove milk fats by *Mirakia* basidiomycete yeasts in Antarctica; and ethanol fermentation by *M. blollopis* at low temperatures. This somewhat eclectic mix of topics provides a flavour of the achievements of the Japanese teams working at and with cultures isolated from the two polar regions, though I fear some may have expected more from a volume with such a broad title.

**Fig. 40:**
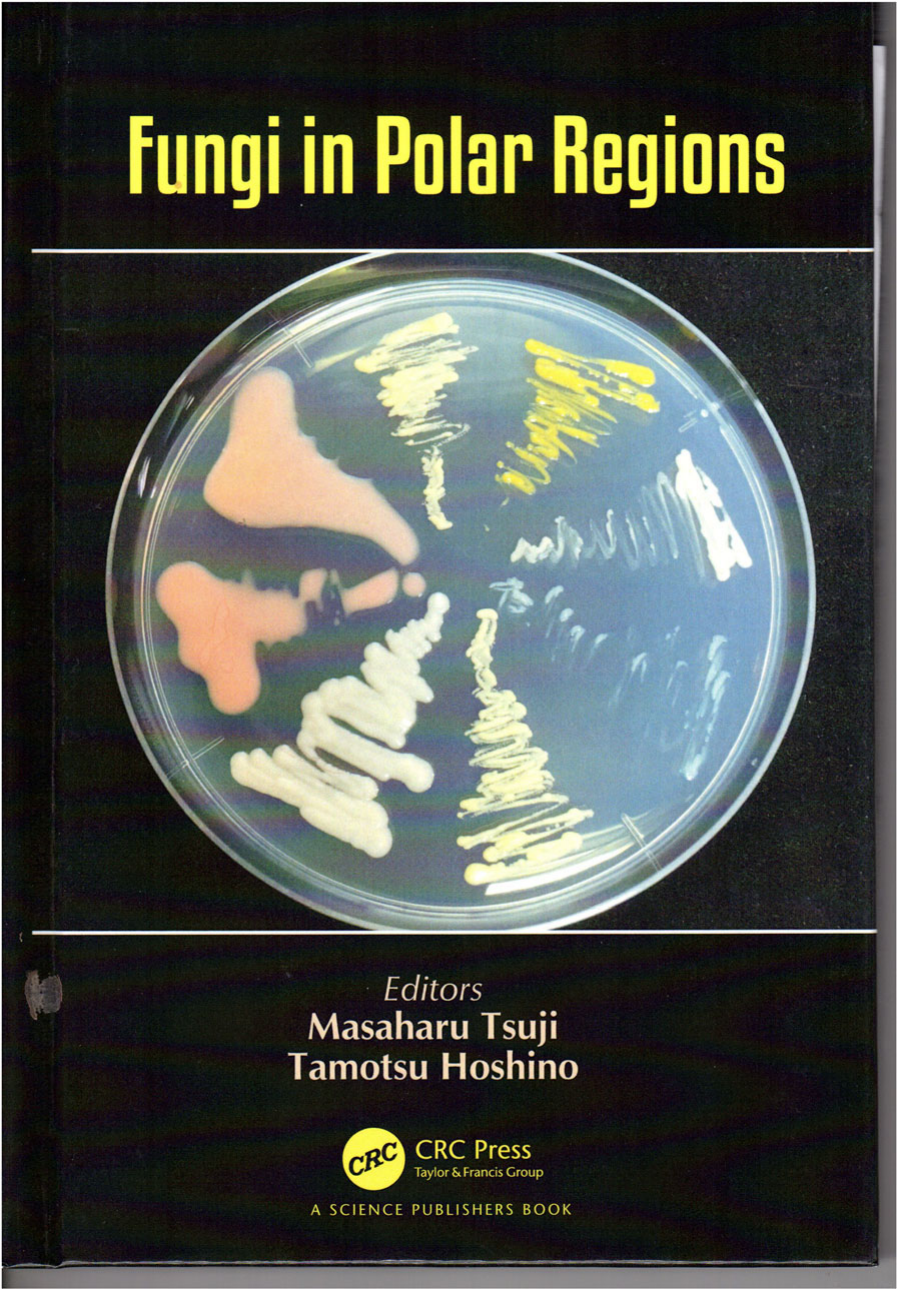
*Fungi in Polar Regions* (2019)

### Illustrated generic names of fungi: etymology, descriptions, classifications, and references

#### By Miguel Ulloa and Elvira Aguirre-Acosta. 2020. American Phytopathological Society Press, St Paul, MN. Pp. xiii + 451, 1052 illustr. (col). ISBN 978-0-89054-618-5 (hbk), 978-0-89054-619-2 (ebk). Price: US$ 199.00 (hbk) (Fig. 41)

Mexican mycologist Miguel Ulloa is well-known for his previous illustrated works on fungi, not least the illustrated dictionary (Ulloa & Hanlin 2000, 2006, 2012). This new book is essentially an updated and much expanded version of his *Etimologia e Iconografia de Géneros de Hongos* (Ullloa & Herrera 1994). That work included 807 genera, was arranged systematically, illustrated in black-and-white, and in Spanish; this replacement volume has a staggering 1592 genera, is arranged alphabetically, illustrated by original watercolours, and in English. It is undeniably the most comprehensive collection of generic descriptions available, though it has to recognized that there are now over 6000 generic names currently in use. The authors have had a difficult task of selecting just what to include, and the selection is inevitably eclectic. They have, however, not restricted themselves to kingdom *Fungi*, but also included some examples from *Chromista* and *Protozoa* (at the end and out of the main A–Z sequence)*,* and also endeavoured to have a good spread of systematic and ecological groups, not only including lichen fungi but even some relatively obscure lichenicolous fungi. For each generic entry, author citations are included, and information is then presented on the hierarchical classification, original place of publication of the name, and etymology – which sometimes extends into brief descriptions and notes on ecology and distribution. In some cases synonyms are also indicated or cross-referenced, and the taxonomy is generally up-to-date and includes quite a few recently introduced names. In addition, recommendations of working groups as to which of competing names to adopt under the 1 *N* = 1F system are followed; for example, *Pleospora* is indicated as a synonym of *Stemphylium*.

**Fig. 41:**
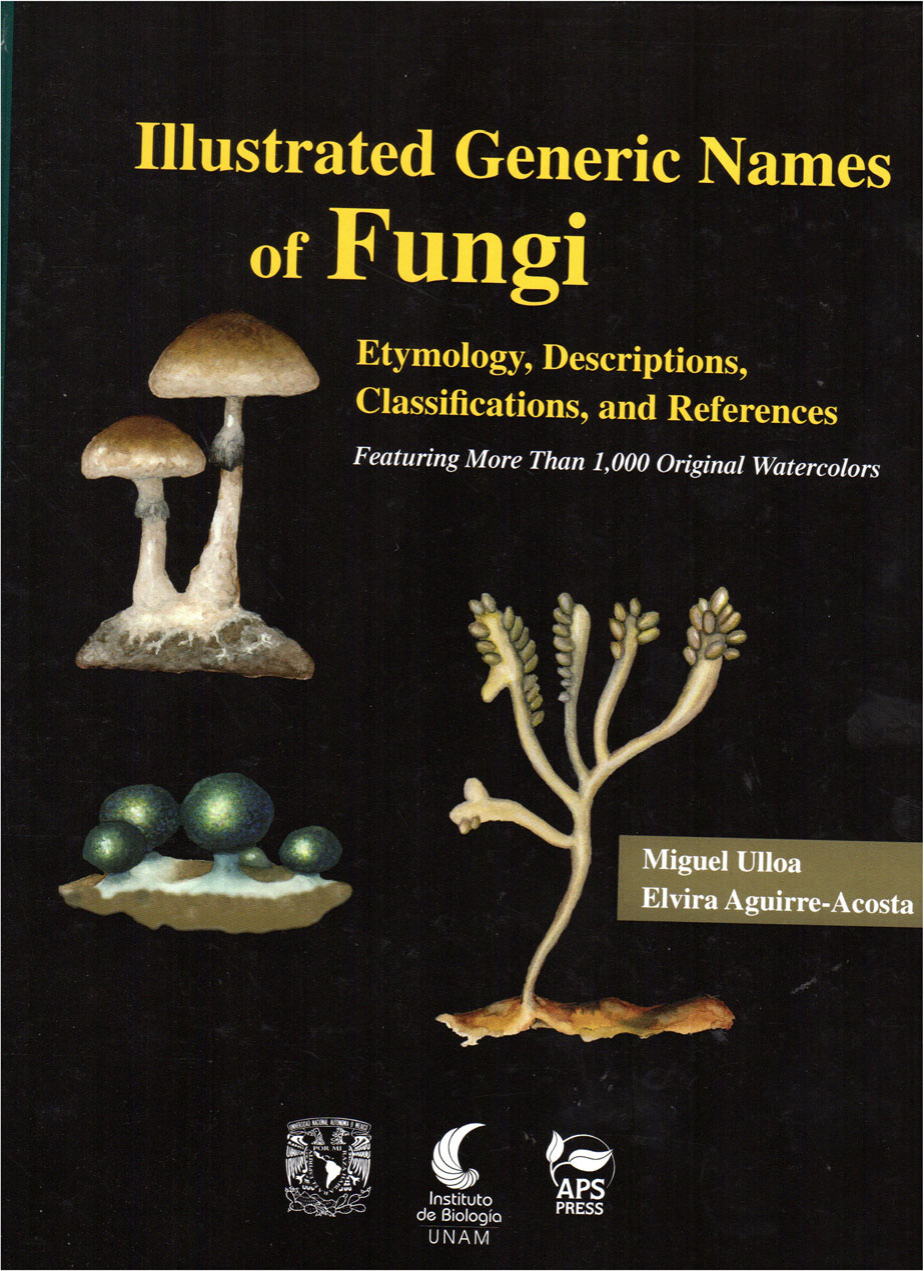
*Illustrated Generic Names of Fungi* (2020)

Illustrations are provided for 1052 genera, about two-thirds of those included, and have clearly been painstakingly prepared largely by Miguel as watercolours; a truly enormous achievement! Each is annotated giving the name of the species selected, notes on its habitat, key features or uses, and magnification. Some depict the overall habit, others microscopic details, and in some instances even single spores as seen in the SEM. There are not unexpectedly some mistakes, and there could perhaps have been a “health warning” as users need to be aware that such large works will always have mistakes; in the last *Dictionary* in which I was involved our aspiration was to have produced a “marvellously imperfect work needed by all” (Hawksworth et al. 1995: vii)! Just a couple of examples amongst illustrations in the “As”: the terricolous *Alectoria ochroleuca* is figured “on a conifer twig” not on the ground, is not yellow, and has a different branching habit; and that of *Anaptychia ciliaris* appears rosette-like, not laciniate and pendent, is olive not pale grey, and is shown with a “ciliate border” not with hair-like extensions at the lobe ends. I suspect that such issues may result from working from inadequate or wrongly named illustrations rather than actual specimens; it would be unrealistic to expect two authors to be personally familiar with such a wide range of organisms.

I would have preferred to see space given to references to recent treatments of the selected genera rather than to the places of original publication, but such information is provided at the generic level in editions of *Ainsworth & Bisby’s Dictionary,* though the latest is now rather dated (Kirk et al. 2008). The use of this new generic treatment, and that *Dictionary*, supplemented by the superbly largely photographically illustrated guide to the then accepted families of Cannon & Kirk (2007), together provide an excellent basis for fungal systematics. It would have been lovely to have had all those to hand when I was starting out in mycology! Producing this new work has clearly been an enormous labour, and demonstrates both the authors’ love of fungi and their commitment to communicating that knowledge worldwide. They are to be congratulated on putting together something that no one has previously done; and perhaps they should be encouraged to include all accepted genera in some future edition. The down-side is that the price is, understandably, rather high for the aspiring individual mycologists that might find it of the greatest value (though there is a 10% discount for APS members), but this is certainly something that all mycological libraries should obtain and make as accessible as they can.

### The secret life of fungi: discoveries from a hidden world

#### By Aliya Whiteley. 2020. Elliott & Thompson, London. Pp. xii + 195. ISBN 978-1-78396-530-4. Price £ 11.99 (Fig. 42)

I do not think there can be too many books to publicise the importance of fungi on Earth and in our lives. The author lives and walks around the fields and woods in West Sussex in south-eastern England, and first became fascinated in the fungi she encountered when living in north Devon. She has done a commendable amount of reading and online research, resulting in an eight-page bibliography. As an already experienced author, she chose to present her discoveries in 30 soundbite-like chapters each of 4–6 pages, which make this handy-sized hard-back easy to dip into in spare moments or on stop-start journeys. These are organized into three groups, erupt, spread, and decay, and have fascinating titles including: Fruiting cities, Slow dancers, thrown high, Spire, Under Alice, Stowaways of the space age, Killer club, and Grasp. In a popularist and engrossing style, she educates readers about the diversity, ecology, roles, and threats posed by fungi of all kinds (including I was pleased to see lichen fungi). Illustrations are confined to a half-tone drawing at the start of each chapter. A reading list of fungal fiction of 15 works is also provided (p. 185), including one of her own I have now obtained but not yet had time to read (Whiteley 2014), but lacking several rather delightful to somewhat scary ones (e.g. Cameron 1954, Lumley 1993, Thomson 1973). This is not for the specialist, has the inevitably odd factual error, and could have benefitted from a checking over by a professional mycologist (e.g. “the *Ascomycota* species”, *Penicllium notatum* not *P. rubrum* for Fleming’s fungus). The book could, however, serve well as a Christmas gift for someone you wanted to either get fascinated by fungi themselves or understand why you devote so much time to them. In an Afterword she aptly comments: “that fungi are all over us, around us, and in us, so this is not a world we can choose to ignore, or escape, because it’s their space just as much as it’s ours” (p. 174). This book promises help to broadcast that message, and it deserves a place on the natural history shelves of high-street bookstores. Any member of the public that buys it is unlikely to be disappointed, and will never look at fungi in quite the same way again – and if they want to enquire further, *Entangled Life* (see above) can then be commended.

**Fig. 42:**
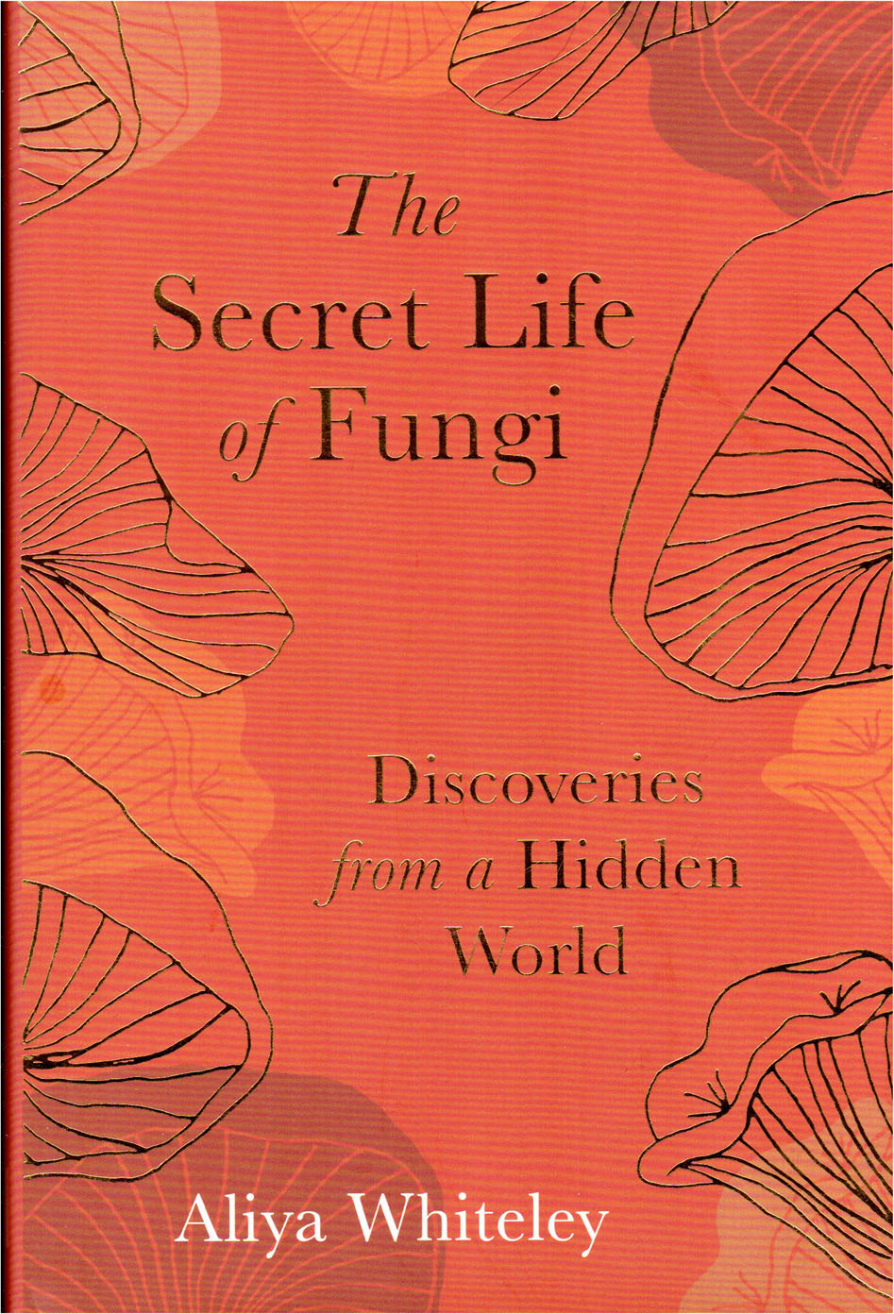
*Secret Life of Fungi* (2020)

## CORRESPONDENCE

### The term funga: *pro et contra*

This letter is intended to continue a discussion started in *IMA Fungus* pages, with contributions by Hawksworth (2010) and Kuhar et al. (2018), who argued for a wider use of the term ‘funga’. They did this to provide a substitute term for ‘mycobiota’ in fields where the term ‘flora’ (or ‘mycoflora’) was previously used, particularly in biodiversity inventory and its biogeographic analysis, as well as applied fields. This problem is especially acute for fundamental works relevant in the long-term, notably ‘multi-volume ‘Flora’ series. This new direction was initiated by Knudsen & Vesterholt (2008), who used the term in the title of their book, and this was enthusiastically followed by some authors. Wide implementation of this proposal would, however, face difficulties due to avoidance of this term by many mycologists for a variety of various reasons. The main arguments for and against the term may be summarized as:

#### Pro

1. *Fungi* are now accepted as a separate kingdom of eukaryotes, and so require an independent terminology.
2. Fungi, plants, and animals are the most diverse groups of multicellular organisms, and where the terms ‘Flora’ and ‘Fauna’ are traditionally used in publications, ‘Flora’ is used to include the fungi so there is a need for an equally short term that provides the conceptual significance of their separateness.
3. The term ‘Funga’ is concordant with the brevity, genus, and terminations of the terms ‘Flora’ and ‘Fauna’, and together the three form a convenient conceptual abbreviation (FF&F).

#### Contra

1. Funga is a polysemantic word, not purely Latin, and a composite with the ending modified (as for ‘Flora’ and ‘Fauna’).
2. Unlike ‘Flora’ and Fauna’, associated with a centuries-old tradition and referring to corresponding ancient deities, ‘Funga’ is not rooted in ancient mythology, even though Kuhar et al. (2018) associated it with the goddess Diana. The term ‘Diana’ would therefore be more consistent with this logic, although it also does not refer to any tradition.
3. A consequence of the rich history of usage of the terms ‘Flora’ and ‘Fauna’ was the emergence of two main derivative terms, ‘floristic’ and ‘faunistic’ (including such subvariants as ‘algofloristic’, ‘mycofloristic’, and ‘prostistofaunistic’). Taking the term ‘Mycobiota’ as an example, for the almost 30 years of its existence, researchers have been reluctant to coin derivatives (‘mycobiotic’ and ‘mycobiotistics’) and a similar fate is likely for potential derivatives from the term ‘Funga’.
4. The tradition of associating the plant world with ‘Flora’, and the animal world with ‘Fauna’ was a consequence of empirical evidence: multicellular animals were characterized by activity, whereas subjects of the plant world were characterized by their inability to change location. A review of the “plant world” polyphyly hasn’t yet led to development of special biodiversity-analytical and biogeographic terminology for each lineage, although many “plant” lineages are diverged from each other deeper than fungi from animals.
5. If ‘Fungi’ were to be limited to use as the name of a kingdom, then other organisms traditionally studied by mycologists and treated with them for the purposes of nomenclature, so-called fungal analogues and pseudofungi, should logically also have separate floristic analytical terms; i.e. *Acrasiomycota* (*Discoba* supergroup), *Myxomycota* (*Amoebozoa*), *Plasmodiophoromycota* (*Rhizaria*), *Labyrinthulomycota*, and *Oomycota* (*Straminipila*). Discussion on the introduction of ‘Funga’, should consequently consider terminological decisions for these lonely remnants of the historical plant kingdom in the overall phylogenetic system of eukaryotes.

If we admit that the burden of the *contra* arguments is too great, then there are two alternatives for mycologists: either continue to use terms derived from Flora, or adhere to a faunistic terminology. The first, using Flora-related terms applied to fungi, would correspond most with the evidence: fungi are static rather than mobile. Further, historically, the majority of authors of the 17th–20th centuries have associated fungi and fungus-like organisms with plants. The second, using fauna-related terms, seems not to have been implemented (a Google query for ‘Mycofauna’ yielded only 203 results), although in some very old systems the fungi were interpreted as closest relatives of the sponges (the term Funga is etymologically related to Spongia) or other ‘phytozoans’. Phylogenetically, there is a sound basis for such juxtaposition: the opisthokont clade (holozoa + holomycota) together with the clades of apusomonads form a single monophyletic group (*Obazoa*), all the basal lineages of which are represented by amoeboid protozoans. In such a hypothetical solution, the terms ‘Fauna of fungi’, ‘Fungal fauna’, or ‘Mycofauna’ would be understood by analogy with a real-life concept of ‘sporozoan fauna’. In this connection, the ascomycete genus *Pneumocystis* represents an interesting example, as it was long-studied by parasitologists and protozoologists but not mycologists.

Also important is the issue of derivative terms for use in floristic analytical studies. The following floristic analytical terms are still not addressed by proponents of the term ‘Funga’.

- Floristics, e.g. methods of floristics, comparative floristics.
- Floristic, e.g. floristic analysis, ecological floristic elements.
- Flora-analytical, e.g. flora-analytical methods.
- Flora-coenotic, e.g. flora-coenotic complexes.
- Floroid (an incomplete flora), e.g. list obtained from a foray.
- Palaeoflora, e.g. Eocene palaeofloras.

If we create Funga-based terms for all described cases, such a wide range of new terms has the prospect of creating more barriers to communication with the wider community so should not be encouraged (D. L. Hawksworth, *in litt*.).

A rather long transitional period awaits, during which Funga, Mycobiota, Mycoflora and perhaps terms based on them, will compete. One day, however, ‘Funga’ may emerge as the basic term covering fungi and fungal analogues, rather than kingdom *Fungi*, for the broader historically rooted union. I would, however, like to see ‘Funga’ increasingly adopted by mycologists for treatments of the fungi in particular regions, especially for major books (cf. Knudsen & Vesterholt 2008) and any new multi-volume series, where ‘Flora’ would otherwise have been used.

I am grateful to David L. Hawksworth for his kind valuable comments and improvements. This work was supported by State Research Task N AAAA-A19-119020890079-6.

**Ivan V. Zmitrovich**

Laboratory of Systematics and Geography of Fungi, Komarov Botanical Institute of the Russian Academy of Sciences, 2 Professor Popov Street, St Petersburg 197376, Russia

(iv_zmitrovich@mail.ru)

## NOTICES

1. *MycoNews* is compiled by David L. Hawksworth as Editor-in-Chief, and to whom material for consideration for inclusion in *MycoNews* should be sent directly by e-mail
2. Books for possible coverage in the Book News section should be mailed to David L. Hawksworth at Milford House, 10 The Mead, Ashtead, Surrey KT21 2LZ, UK; works issued only as e-books are not normally included, but reviews prepared by others will also be considered if sent to him.
3. Reports of new genome sequences intended for inclusion in the *Fungal Genomes* compilation should be sent directly to Senior Editor Brenda Wingfield as e-mail attachments and not submitted through Editorial Manager.
4. All unsigned items in *MycoNews* can be attributed to the compiler, David L. Hawksworth.

## References

[CR1] Baldini RM (2020). The impact of Covid-19 crisis on plant taxonomy: will we be able to approach to plant taxonomy as in the past?. Webbia.

[CR2] Biesmeijer K, van Wezel A, Crous P, Kok J (2020) ARISE: op zoek naar de onzichtbare biodiversiteit. De Levender Natuur 121: 177–180.

[CR3] Blencowe C (2020). Response to Coronavirus: #MycoBookClub. Br Mycol Soc Newsl.

[CR4] Borges LM, Reis VC, Izbicki R (2020) Schrödinger’s phenotypes: Herbarium specimens show two-dimensional images are both good and (not so) bad sources of morphological data. Methods Ecol Evol 11:1296–1308

[CR5] Buck WR (2009) Biographical sketch: Richard Clinton Harris, the quintessential North American lichenologist. Opusc Philolichenum 7:viii–xxvi

[CR6] Buck WR (2016). The Tuckerman Lichen Workshop and the Crum Bryophyte Workshop: a brief history. Evansia.

[CR7] Cameron E (1954). The Wonderful Flight to the Mushroom Planet.

[CR8] Cannon PF, Kirk PM (2007). Fungal Families of the World.

[CR9] Cota-Sánchez JH (2020). The value of virtual natural history collections for botanical instruction in these times of the COVID-19 pandemic. Rev Bras Bot.

[CR10] Coyle D, Brosius T, DeVries Z, Schmidt-Jeffris R, Gott RC (2020). COVID-19: Reflections from entomologists who rose to the occasion. Am Entomol.

[CR11] Crous PW, Verkley GJM, Groenewald JZ, Houbraken J (2019). Fungal Biodiversity. [Westerdijk Laboratory Manual Series no. 1.].

[CR12] Diekonigin C (2020). Response to Coronavirus: a brief history of mycological miscellany. Br Mycol Soc Newsl.

[CR13] Flaherty C (2020) No room of one’s own. Inside Higher Education https://www.insidehighered.com/news/2020/04/21/early-journal-submission-data-suggest-covid-19-tanking-womens-research-productivity

[CR14] Gabster BP, van Daalen K, Dhatt R, Barry M (2020). Challenges for the female academic during the COVID-19 pandemic. Lancet.

[CR15] Harries D (2020). Response to Coronavirus: GLM 2020. Br Mycol Soc Newsl.

[CR16] Hawksworth DL (2010). Funga and fungarium. IMA Fungus.

[CR17] Hawksworth DL, Kirk PM, Sutton BC, Pegler DN (1995). Ainsworth & Bisby’s Dictionary of the Fungi.

[CR18] Kirk PM, Cannon PF, Minter DW, Stalpers JA (2008). Ainsworth & Bisby’s Dictionary of the Fungi.

[CR19] Knudsen H, Vesterholt J (2008). Funga Nordica: agaricoid, boletoid and cypheloid genera.

[CR20] Kuhar F, Furci G, Drechsler-Santos ER, Pfister DH (2018). Delimitation of Funga as a valid term for the diversity of fungal communities: the Fauna, Flora and Funga proposal (FF&F). IMA Fungus.

[CR21] Lendemer JC, Harris RC (2016). The New York Botanical Garden Lichen herbarium: a unique resource for fungal biodiversity research and education. Brittonia.

[CR22] Lumley B (1993). Fruiting Bodies and other Fungi: a witch’s dozen of terror-tales.

[CR23] Manenti R, Mori E, Di Canio V, Mertcurio S, Picone M (2020). The good, the bad and the ugly of COVID-19 lockdown effects on wildlife conservation: insights from the first European locked down country. Biol Conserv.

[CR24] Mułenko W, Ruszkiewicz-Michalska M (2010). Professor Tomasz Majewski. Pol Bot J.

[CR25] Onofri S, Zucconi L, Tosi S (2007). Continental Antarctic Fungi.

[CR26] Pegler DN, Spooner BM, Smith RIL (1980). Higher fungi of Antarctica, the subantarctic zone and Falkland Islands. Kew Bull.

[CR27] Pinnington S, Mynett Y (2020). Response to Coronavirus: Norfolk Fungus Study Group. Br Mycol Soc Newsl.

[CR28] Pitt JI, Hocking AD (2009). Fungi and Food Spoilage.

[CR29] Rose S, Suri J, Brooks M, Ryan PG (2020). COVID-19 and citizen science: lessons learned from southern Africa. Ostrich.

[CR30] Sheard JW (1967). A revision of the lichen genus Rinodina (Ach.)Gray in the British isles. Lichenologist.

[CR31] Sheard JW (2010). The Lichen Genus Rinodina (Lecanoromycetes, Physciaceae) in North America, north of Mexico.

[CR32] Thomson J (1973). Death Cap.

[CR33] Ulloa M, Herrera T (1994) Etimología e Iconografia de Géneros de Hongos. Universidad Nacional Autónoma de México

[CR34] Uolla M, Hanlin RT (2000). Illustrated Dictionary of Mycology.

[CR35] Uolla M, Hanlin RT (2006). Nuevo Diccionario Illustrado de Micología.

[CR36] Uolla M, Hanlin RT (2012). Illustrated Dictionary of Mycology.

[CR37] Walker J (2018) The History of the N.S.W. Plant Pathology and Mycology Herbarium (Herb. DAR) and of taxonomic studies on fungi in the New South Wales Department of Agriculture from 1890 to 2014*.* Privately printed, Rydalmere, NSW

[CR38] Whiteley A (2014). The Beauty.

